# Electrical Conductance and Thermopower of β-Substituted Porphyrin Molecular Junctions—Synthesis and Transport

**DOI:** 10.1021/jacs.3c07258

**Published:** 2023-10-24

**Authors:** Hailiang Xu, Hao Fan, Yuxuan Luan, Shen Yan, León Martin, Ruijiao Miao, Fabian Pauly, Edgar Meyhofer, Pramod Reddy, Heiner Linke, Kenneth Wärnmark

**Affiliations:** † NanoLund, Lund University, Box 118, 22100 Lund, Sweden; ‡ Department of Chemistry, Centre of Analysis and Synthesis, Lund University, Box 121, 22100 Lund, Sweden; § Department of Mechanical Engineering, University of Michigan, Ann Arbor, Michigan 48109, United States; ∥ Institute of Physics and Centre for Advanced Analytics and Predictive Sciences, University of Augsburg, 86159 Augsburg, Germany; ⊥ Department of Materials Science and Engineering, University of Michigan, Ann Arbor, Michigan 48109, United States; # Solid State Physics, Lund University, Box 118, 22100 Lund, Sweden

## Abstract

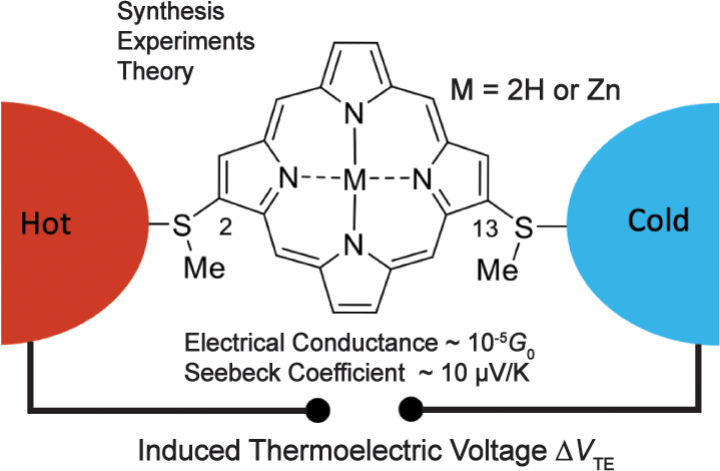

Molecular junctions offer significant potential for enhancing thermoelectric power generation. Quantum interference effects and associated sharp features in electron transmission are expected to enable the tuning and enhancement of thermoelectric properties in molecular junctions. To systematically explore the effect of quantum interferences, we designed and synthesized two new classes of porphyrins, **P1** and **P2**, with two methylthio anchoring groups in the 2,13- and 2,12-positions, respectively, and their Zn complexes, **Zn–P1** and **Zn–P2**. Past theory suggests that **P1** and **Zn–P1** feature destructive quantum interference in single-molecule junctions with gold electrodes and may thus show high thermopower, while **P2** and **Zn–P2** do not. Our detailed experimental single-molecule break-junction studies of conductance and thermopower, the latter being the first ever performed on porphyrin molecular junctions, revealed that the electrical conductance of the **P1** and **Zn–P1** junctions is relatively close, and the same holds for **P2** and **Zn–P2**, while there is a 6 times reduction in the electrical conductance between **P1** and **P2** type junctions. Further, we observed that the thermopower of **P1** junctions is slightly larger than for **P2** junctions, while **Zn–P1** junctions show the largest thermopower and **Zn–P2** junctions show the lowest. We relate the experimental results to quantum transport theory using first-principles approaches. While the conductance of **P1** and **Zn–P1** junctions is robustly predicted to be larger than those of **P2** and **Zn–P2**, computed thermopowers depend sensitively on the level of theory and the single-molecule junction geometry. However, the predicted large difference in conductance and thermopower values between **Zn–P1** and **Zn–P2** derivatives, suggested in previous model calculations, is not supported by our experimental and theoretical findings.

## Introduction

Study of charge transport in molecular junctions (MJs) is of great current interest, with potential applications in molecular electronics^1^ and molecular thermoelectrics.^2^ Over the past decade, many studies were performed to investigate thermoelectric properties at the molecular scale, including those of Au-1,4-benzenedithiol-Au,^1^ Au-4,4′-(ethyne-1,2-diyl)dianiline-Au,^3^ and Au–C_60_–Au junctions.^4^ In recent years, the use of metalloporphyrins in single-molecule junctions (SMJs) has received increasing attention.^5,6^ Porphyrins offer excellent geometrical flexibility and possibilities for electronic tuning,^5^ and well-developed synthetic chemistry allows tailoring their physical and chemical properties by the attachment of substituents on the macrocyclic framework and the choice of the central metal ion.^6^ Strachan et al.^7^ and Yang et al.^8^ studied excited energy transfer rates using porphyrin dimers, which consisted of a free-base porphyrin and a Zn porphyrin. They proposed that the rate of electron transport is determined by the ordering of the occupied frontier orbitals. Tsai and Simpson^9^ further studied theoretically the excited-state energy transfer in donor–acceptor arrays of porphyrins. The study showed a clear correlation between the excited-state configuration and the frontier orbitals and demonstrated that the highest occupied molecular orbitals (HOMOs) dominate the excited energy transfer rates.

Porphyrins have extensively been used to investigate charge transport in molecular junctions.^10^ Most studies of metalloporphyrin in single-molecule junctions to date have focused on the *meso*-substituted systems, involving Ru-, Zn-, Ni-, Co- and Cu-porphyrins.^5,6,10^ Perrin et al.^5^ demonstrated that Ru-TPPdT [5,15-di(*p*-thiophenyl)-10,20-diphenylporphinato]ruthenium(II)(py)_2_, which had two thiophenyl groups at *meso*-positions and two pyridine groups at the axial positions of the Ru(II) porphyrin complex, yielded a reliable configuration between metal electrodes. The introduction of thiol groups and pyridine axial groups resulted in stable molecular porphyrin junctions and an increased spread in conductance compared to the parent porphyrin. Later, Perrin et al.^6^ studied the thiol-terminated zinc porphyrin [5,15-di(*p*-thiophenyl)-10,20-di(p-tolyl)porphinato]zinc(II) in single-molecule junctions, and found that reducing the distance between metal electrodes had an influence on the alignment of molecular orbital levels (both occupied and unoccupied) with respect to the Fermi level. These results showed that the spread in conductance values is caused mainly by image-charge effects in single-molecule junctions. Liu et al.^11^ studied charge transport in different types of metal-porphyrin molecular junctions connected to gold electrodes under low bias. They found that the conductance of single-molecule junctions could change up to a factor of 2, depending on which metal cation the porphyrin contained, and concluded that the metal-porphyrin complexes have a high sensitivity to what metal cation is inserted in the porphyrin core, resulting in single-molecule junctions with tunable conductance and providing important insights into charge transport through complex junctions.

**Figure 1:**
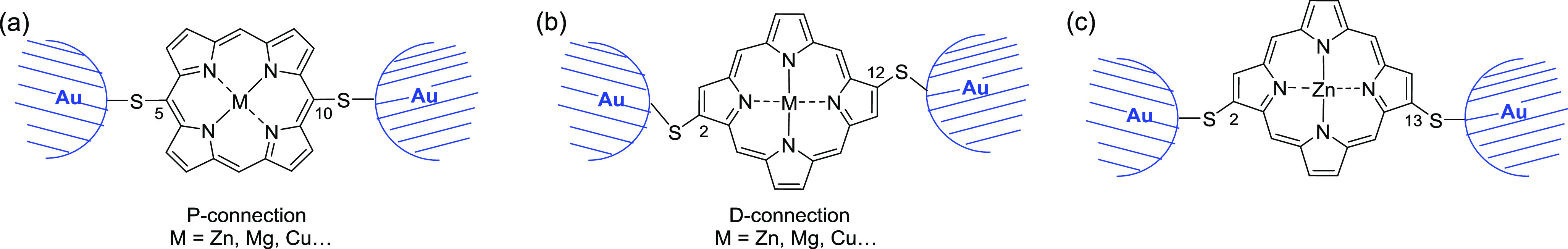
Examples of the different *meso*- and β-substituted metalloporphyrins used to date in the theoretical studies of single-molecule junctions. (a) 5,10-substituted metalloporphyrin of *D*_2h_ symmetry (resulting in a P-connection junction),^12^ (b) 2,12-disubstituted metalloporphyrin of *C*_2h_ symmetry (resulting in a D-connection junction),^14^ and (c) 2,13-disubstituted Zn porphyrin of *C*_2v_ symmetry.^16^ Diagrams adapted with permission from ref (12) Copyright 2009 American Chemical Society, and from ref (16). Copyright 2011 American Physical Society.

Compared to the well-developed experimental and theoretical investigations of *meso*-substituted porphyrin systems, the studies of β-substituted metalloporphyrins in molecular junctions are, to date, only based on theoretical calculations.^12−16^ For example, Wang et al.^12^ used different metalloporphyrins, which were terminated with thiol groups in *meso*-positions (5,15-substitutions; parallel connection, a P-connection; P = parallel) and β-positions (2,12-substitutions; diagonal connection, a D-connection; D = diagonal),^13^ to study the pathway of charge transport in porphyrin-based molecular junctions (Figure 1a,b). According to the calculated *I–V* curves in the theoretical simulations, all models in the P-connection mode were similar, indicating that electron transport is not much influenced by the central metal ion in this configuration. In contrast, the different curves of all models in D-connection indicated that charge transport could be controlled by the central metal ion to tune the conductance of molecular junctions. Karlström et al.^16^ proposed that quantum interference effects in Zn porphyrin-based (2,13-dithioporphinato)zinc(II) molecular junctions (Figure 1c) could selectively filter carriers around the Fermi energy, resulting in high-efficiency thermoelectric energy conversion along with high power output. This theoretical proposition was supported by using a coherent two-level transport toy model.

**Figure 2:**
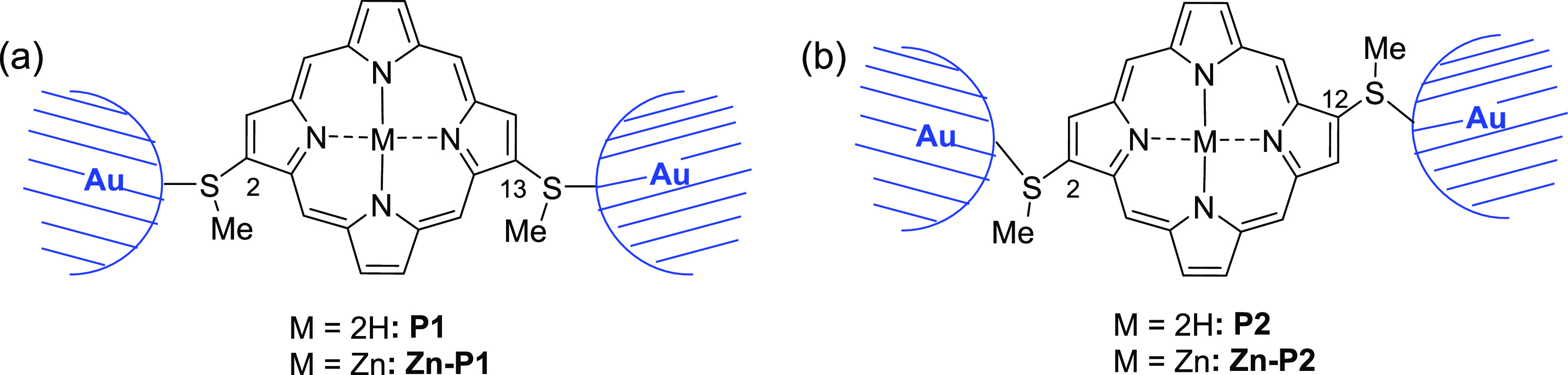
Porphyrins and complexes used in single-molecule junctions in the present study. (a) **P1** and **Zn–P1**, 2,13-bis(thiomethyl)-substituted (*C*_2v_ symmetry). (b) **P2** and **Zn–P2**, 2,12-bis(thiomethyl)-substituted (*C*_2h_ symmetry).

The work by Karlström et al.^16^ inspired us to begin a systematic analysis of the effect of quantum interference on the conductance and thermoelectric properties of β-substituted porphyrin junctions, which have to date not been evaluated experimentally. We therefore designed and synthesized two new classes of porphyrins, studied their electrical conductance and thermopower in single-molecule experiments, and modeled the transport properties via density functional theory. Each of these new porphyrins contains two methylthio groups in the β-position, and their corresponding Zn complexes, namely, 2,13-bis(methylthio)porphyrin (**P1**) and [2,13-bis(methylthio)porphinato]zinc(II) (**Zn–P1**) of *C*_2v_ symmetry and 2,12-bis(methylthio)porphyrin (**P2**) and [2,12-bis(methylthio)porphinato]zinc(II) (**Zn–P2**) of *C*_2h_ symmetry (Figure 2). According to a simple model proposed by Karlström et al., destructive quantum interference should determine the charge transport through single-molecule junctions containing **P1** and **Zn–P1**, while **P2** and **Zn–P2** serve as reference compounds. Working with gold electrodes, the two methylthio groups were chosen because they could establish a strong electronic coupling to gold through covalent binding^17^ and connect selectively to single gold atoms, forming a well-defined metal-molecule interface. Finally, they are robust in multistep synthesis ranging from the starting material to the final porphyrin. Single-molecule quantum transport studies with different positions of the methylthio groups in the porphyrin framework can provide a better understanding of the electronic pathway and the influence on the thermoelectric properties. Likewise, the presence or absence of the Zn^2+^ ion coordination to the four nitrogen atoms in the core of the porphyrin is expected to influence the energetic alignment of the HOMO and lowest unoccupied molecular orbital (LUMO) frontier orbitals, as observed for TPP and ZnTPP (TPP = 5,10,15,20-tetraphenylporphyrin) through quantum chemical (QC) calculations,^18^ which in turn is expected to influence the electric and thermoelectric transport properties.

## Results and Discussion

### Synthesis of Free-Base Porphyrins and Zinc Porphyrin Complexes

Porphyrin synthesis has progressed rapidly over the last century.^19−27^ An increasing number of methods have been reported for the synthesis of porphyrins substituted in β- or *meso*-positions with the same or different functional groups.^28−50^ Depending on the symmetry of the targeted porphyrins, the choice of the synthetic route focuses on three typical methods: monopyrrole tetramerization,^28−38^ MacDonald [2 + 2],^39−42^ and [3 + 1]^43,45−50^ routes.

#### Retrosynthesis of Target Porphyrins

As discussed above, we desired porphyrins without substituents in the *meso*-positions, i.e., that are substituted in the 2,13- (**P1**) and 2,12-positions (**P2**), respectively, and these classes of porphyrins are to date unknown, except for one example.^51^ Hence, a reliable methodology to synthesize both **P1** and **P2** had to be developed, and we reasoned that, most probably, a similar procedure could be used for the synthesis of porphyrins substituted in the 2,13- and 2,12-positions, respectively.

**Scheme 1:**
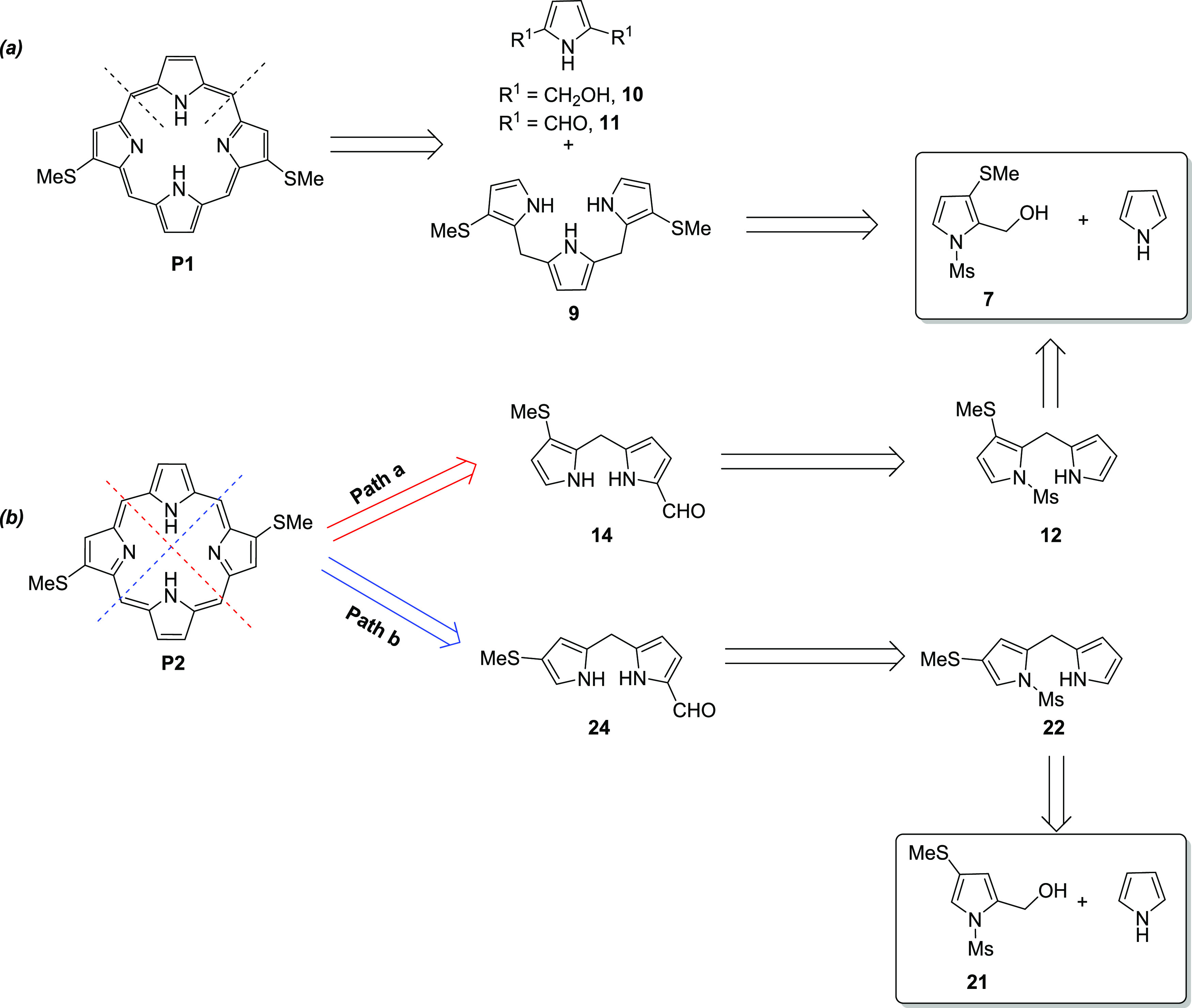
Retrosynthesis of the Targeted Porphyrins (a) [3 + 1] Approach to **P1**. (b) Two Possible Ways for the Synthesis of **P2** through the MacDonald [2 + 2] Method

In porphyrin synthesis, the efficiency of the ring-closing step is the key to obtaining a high overall yield. According to the symmetry and substitution pattern of the targeted porphyrins **P1** and **Zn–P1**, the [3 + 1] route, using the condensation of tripyrrane (**9**) and 1*H*-pyrrole-2,5-dimethanol (**10**) or 1*H*-pyrrole-2,5-dicarbaldehyde (**11**), appears to be the best approach to obtain porphyrin **P1**, Scheme 1a. The reagent used for the ring-closing step can be a Lewis acid or a Brønsted acid, such as the trifluoroborane (BF_3_)-MeOH complex^49^ and TFA,^50^ respectively. Tripyrranes are not stable under air and acidic conditions,^50,52^ and tripyrranes without substitution in the 2-position are easily oxidized by air. Tripyrrane **9** is synthesized from the condensation of *N*-methylsulfonylpyrrole carbinol (**7**) with pyrrole, which can then be deprotected under mild conditions. The methylsulfonyl (Ms) group is chosen as a protecting group, because of the following three reasons:^53^ (1) *N*-Ms substituted tripyrranes are very stable under acidic conditions; (2) the Ms group is easily removed under basic conditions; and (3) the electron-withdrawing effect of the Ms group can slow down the unwanted self-condensation of pyrrole carbinols.

Based on the symmetry and substitution pattern of the targeted porphyrins **P2** and **Zn–P2**, two possible pathways of self-condensation using dipyrromethanes (**14** or **24**) can provide **P2**, as shown in Scheme 1b. The only difference between these two dipyrromethanes is the position of the thiomethyl (SMe) group. In pathway **a**, **14** can be obtained from dipyrromethane **12** in two steps: Ms deprotection and formylation. The condensation between **7** and pyrrole affords **12**. In pathway **b**, compound **24** can be synthesized in the same way from dipyrromethane **22**.

### Synthesis of *C*_2v_-Symmetric **P1** and **Zn–P1**

**Scheme 2:**
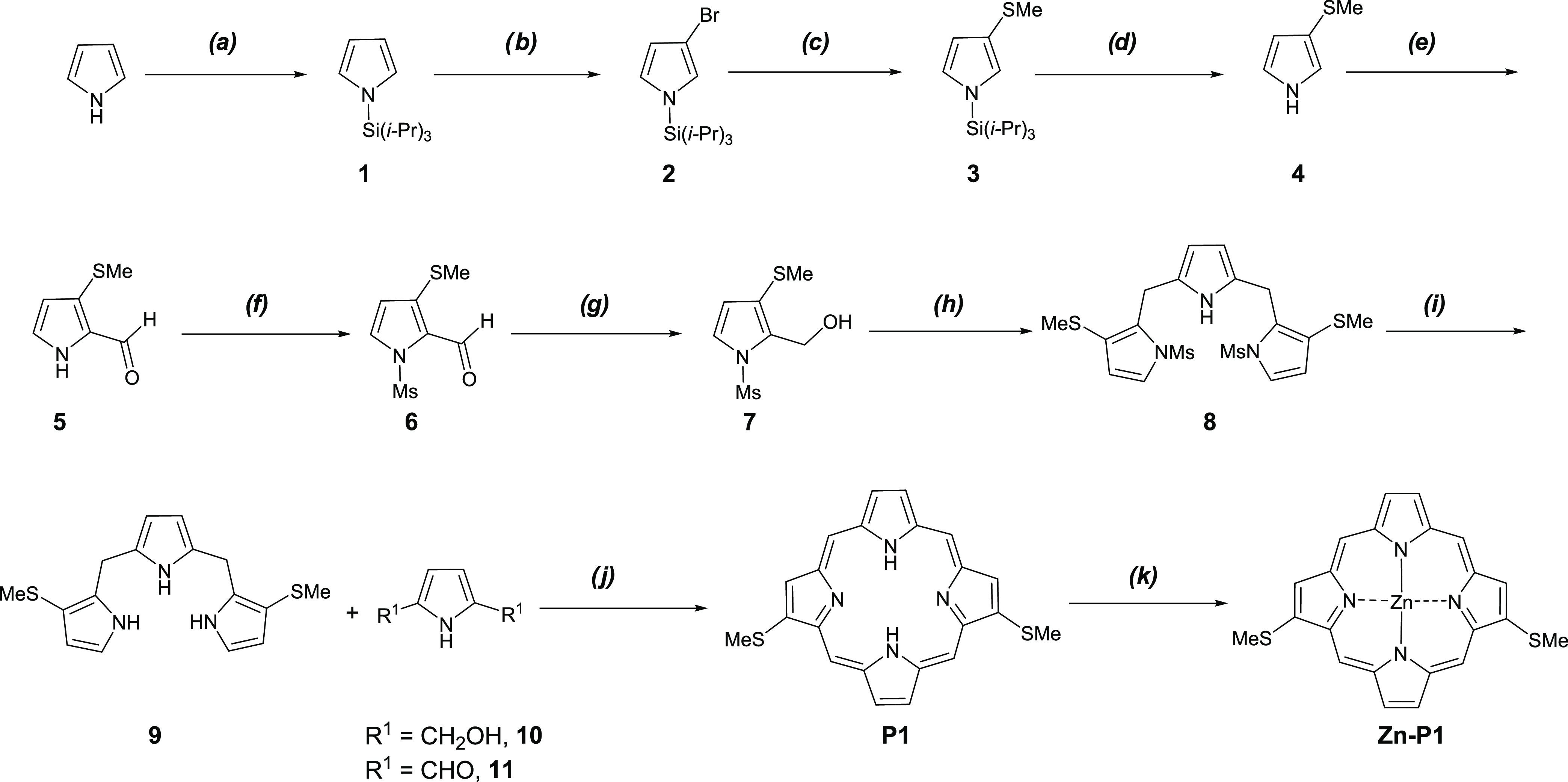
Synthesis of Free-Base Porphyrin **P1** and Complex **Zn–P1** (a) (1) *n*-BuLi, THF, −78 °C, 30 min; (2) TIPSCl, −78 °C to rt, overnight, 99%; (b) NBS, THF, −78 °C, 5 h, 97%; (c) (1) *n*-BuLi, THF, −78 °C, 8 h; (2) MeSSMe, −78 °C to rt, overnight, 90%; (d) TBAF, THF, rt, 20 min, 100%; (e) (1) POCl_3_, DMF, 1,2-dichloroethane, reflux, 2 h; (2) NaOAc (aq.), 1 h, 86%; (f) MsCl, NaH, THF, rt, overnight, 83%; (g) NaBH_4_, MeOH/THF, rt, 2 h, 94%; (h) pyrrole, TFA, CH_2_Cl_2_, reflux, 4 h, 42%; (i) KOH, MeOH, N_2_, rt, overnight, 96%; (j) (1) 11, BF_3_·MeOH, MeOH/CHCl_3_, rt, 1 h; DDQ, rt, 30 min, 4%; (2) 12, TFA, CHCl_3_, rt, 20 min; DDQ/toluene, 10 min, 9%; (k) Zn(OAc)_2_·2H_2_O, MeOH/CH_2_Cl_2_, rt, overnight, 90%.

Based on the above retrosynthesis, see Scheme 1a, we designed a synthetic route to **P1** and **Zn–P1**, see Scheme 2. Generally, direct C–H bromination of pyrrole results in 2-bromo- and/or 2,5-dibromopyrrole, depending on the amount of the bromination reagent.^54^ Since in pyrrole, positions 2 and 5 are more reactive to electrophilic halogenation than positions 3 and 4, 3-bromopyrrole can be obtained by direct C–H bromination of pyrroles containing bulky *N*-protection, such as *N*-triisopropylsilyl pyrrole (*N*-TIPS pyrrole, **1**). The steric bulk of the TIPS group shields the positions 2 and 5, affording selective bromination at positions 3 or 4, which are further away from the protected nitrogen.^55^ The protection of pyrrole with the TIPS group^56^ afforded **1** in 99% yield. *N*-TIPS pyrrole **1** was treated with 1 mol of *N*-bromosuccinimide (NBS) in tetrahydrofuran (THF) at −78 °C^57^ to give the 3-bromo-substituted pyrrole **2** in 97% yield. The amount of NBS should be accurate; otherwise, a mixture of 3-bromo- and 3,4-diboromo-substituted pyrroles would be formed, and the mixture is problematic to separate.

Pyrrole **2** was treated with *n*-butyllithium (*n*-BuLi) at −78 °C, followed by the addition of dimethyl disulfide (MeSSMe) to afford methylthiopyrrole **3** in 90% yield. The deprotection of methylthiopyrrole **3** was carried out by treatment with tetrabutyl ammonium fluoride (TBAF)/THF at room temperature to provide an unstable compound **4** in quantitative yield. Pyrrole **5** was prepared in 86% yield by formylation of **4** using the Vilsmeier–Haack reaction (phosphoroxychloride/*N*,*N*-dimethylformamide (POCl_3_/DMF)). The formylation was highly regioselective for the 2-position of **4**, since the SMe group is an electron-donating group (EDG), and the unshared pairs of electrons on sulfur activate the 2-position more than the 5-position by resonance. The formylated pyrrole **5** was treated with sodium hydride (NaH) in THF, followed by the addition of methanesulfonyl chloride (MsCl), to afford compound **6** in 83% yield. *N*-mesylpyrrole carbinol **7** was obtained in 94% yield by the reduction of **6** using sodium borohydride (NaBH_4_) in a mixture of methanol (MeOH) and THF at room temperature for 2 h.

We investigated whether a synthetic route to tripyrrane **8** was viable by optimizing the condensation between pyrrole and **7** (Table S1). The best reaction condition for the synthesis of **8** was trifluoroacetic acid (TFA) (0.5 equiv), methylene chloride (CH_2_Cl_2_), reflux, 4 h, resulting in a 42% yield of **8** (Table S1, entry 9). The following deprotection of **8** under basic conditions (potassium hydroxide (KOH), methanol (MeOH), N_2_, room temperature, overnight) gave rise to tripyrrane **9** in 96% yield. Due to instability, tripyrrane **9** should be used in the following [3 + 1] route for the ring-closing step with little delay (Scheme 2). Tripyrrane **9** was condensed with 2,5-bis(hydroxymethyl)pyrrole **10** using BF_3_·MeOH in a mixed solvent (MeOH and CHCl_3_) at room temperature for 1 h, followed by oxidation accomplished by the addition of DDQ to afford the targeted free-base porphyrin **P1** in less than 4% yield. Compared to **10**, *1H*-pyrrole-2,5-dicarbaldehyde **11** worked better in the condensation. Hence, to the solution of TFA in CHCl_3_, **9** and **11** in CHCl_3_ were added dropwise simultaneously at room temperature for 15 min, followed by oxidation by DDQ and neutralization with triethanol amine to afford free-base porphyrin **P1** in 9% yield. The reaction was conducted on a small scale in diluted CDCl_3_ (<1.0 mmol) in the dark each time. The corresponding metalloporphyrin **Zn–P1** was obtained with 90% yield using **P1** and zinc acetate dihydrate in a mixed solvent (MeOH and CH_2_Cl_2_) by stirring at room temperature overnight. In total, **Zn–P1** was synthesized with a 1.9% yield over 11 steps from pyrrole.

### Synthesis of *C*_2h_-Symmetric **P2** and **Zn–P2**

**Figure 3:**
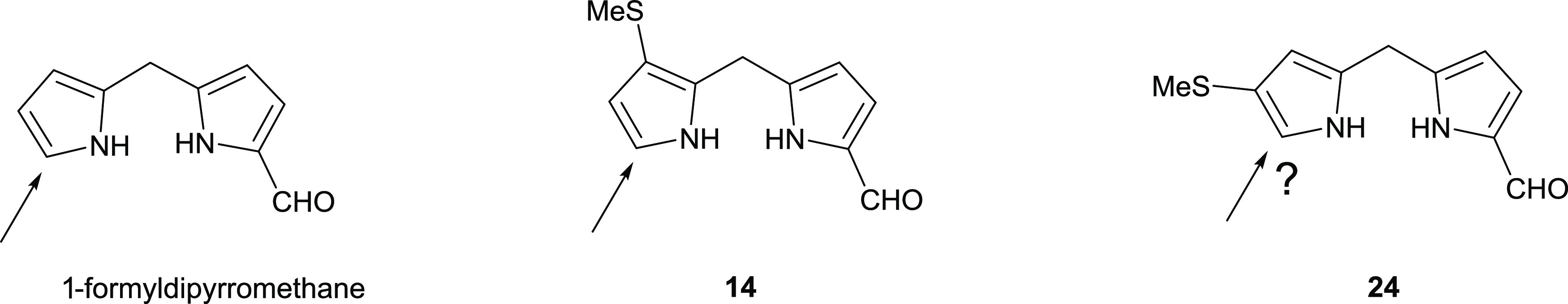
The expected position of electrophilic attack on the differently methylthio-substituted 1-formyldipyrromethanes in this work.

**Scheme 3:**
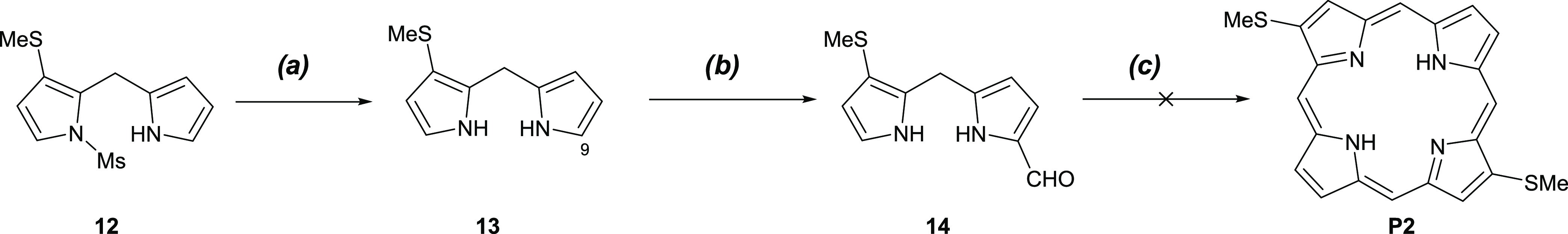
Attempted Synthesis of Free-Base Porphyrin **P2** According to Path **a** of Scheme 1b (a) KOH, MeOH, N_2_, rt, overnight, 91%; (b) (1) POCl_3_, DMF, 1,2-dichloroethane, reflux, 2 h; (2) NaOAc (aq.), 30 min, 64%; (c) (1) MgBr_2_, DBU, toluene, air, 115 °C, 19 h; (2) TFA, DCM, rt, 30 min, then Et_3_N.

The first attempted synthetic pathway started from dipyrromethane **12**. The starting material was obtained following path **a** in the retrosynthesis, Scheme 1b, by the condensation of **7** and pyrrole (1:2) in HCl/H_2_O to afford **12** in 84% yield (Table S1, entry 2). The following Ms deprotection under basic conditions produced **13** in 91% yield, as shown in Scheme 3. The formylation of **13**, using Vilsmeier–Haack conditions (POCl_3_/DMF), led to the formation of **14** in 64% yield. The ^1^H–^1^H NOESY showed that formylation took place in the 9-position of **13**. The self-condensation of **14**, using the method in Lindsey’s report,^40^ failed to provide **P2**. Compared to 1-formyldipyrromethane, the 9-position in **14** is obviously less reactive to condensation in the presence of an SMe group attached in the 8-position (compound **24**, Figure 3). The alternative route is as indicated in the retrosynthesis, Scheme 1b, where the SMe group has been moved from position **7** in 14 to the 8-position instead, resulting in dipyrromethane **24**, as the starting material for **P2** and **Zn−P2**.

**Scheme 4:**
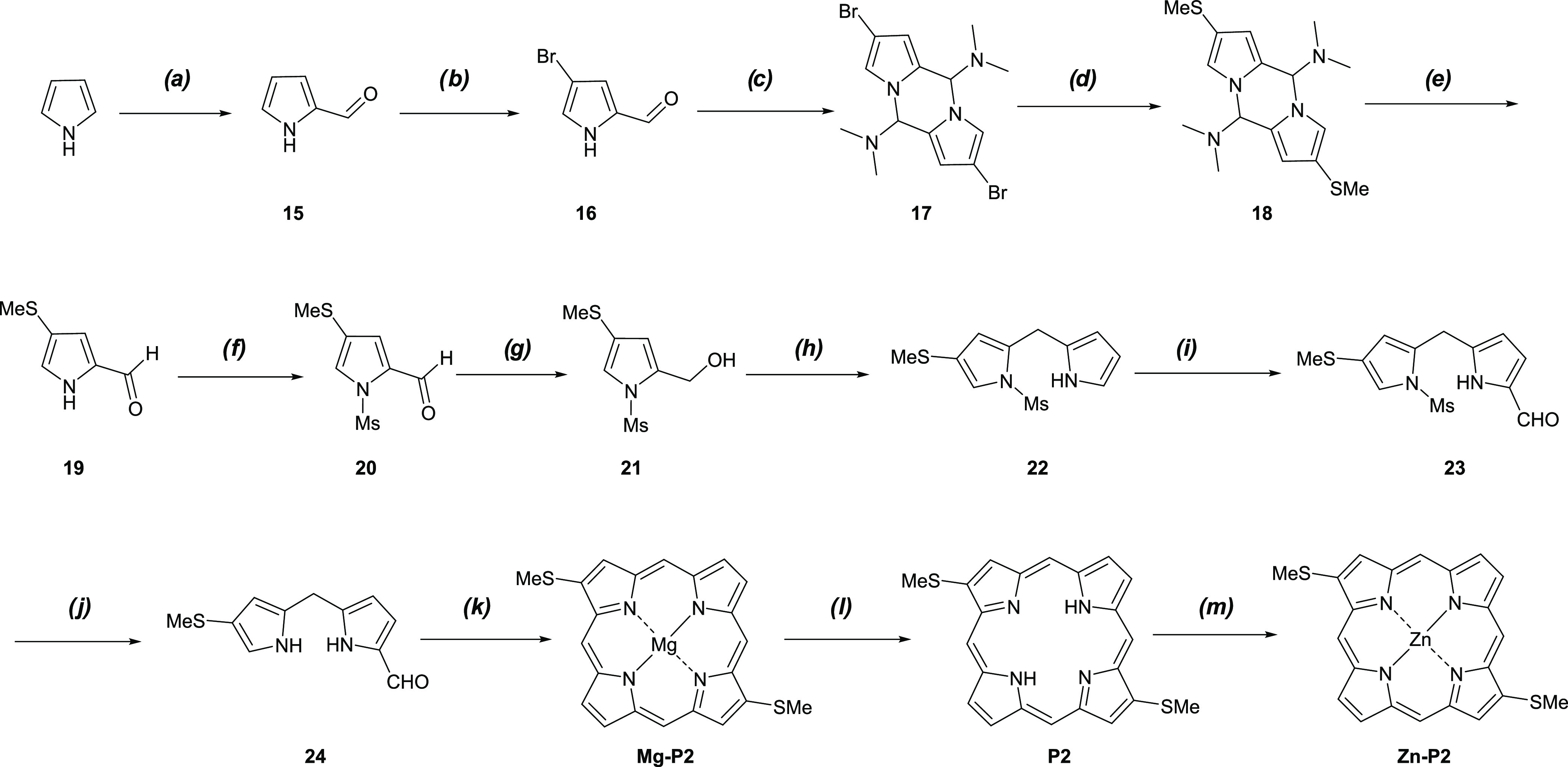
Synthesis of Free-Base Porphyrin **P2** and Complex **Zn–P2** (a) (1) POCl_3_, DMF, 1,2-dichloroethane, reflux, 2 h; (2) NaOAc (aq.), 30 min, 90%; (b) NBS, THF, −78 °C, 7 h; (c) Me_2_NH, H_2_O, rt, 3.5 h, 70% (2 steps); (d) (1) *t*-BuLi, THF, −78 °C, 1 h; (2) MeSSMe, −78 °C to rt, overnight, 73%; (e) NaHCO_3_ (aq.), reflux, 15 h, 99%; (f) MsCl, NaH, THF, rt, overnight, 76%; (g) NaBH_4_, MeOH/THF, rt, 1 h, 98%; (h) pyrrole, BF_3_·CH_3_OH, MeOH/CHCl_3_, 50 °C, 16 h, 70%; (i) (1) POCl_3_, DMF, 0 °C, 2 h; (2) NaOAc (aq.), 1,2-dichloroethane, 1 h, 83%; (j) NaOH (5M), MeOH/H_2_O, reflux, 3 h, 89%; (k) MgBr_2_, DBU, toluene, air, 115 °C, 19 h, 70%; (l) (1) TFA, CH_2_Cl_2_, 0 °C, 20 min; (2) Et_3_N, 50%; (m) Zn(OAc)_2_·2H_2_O, THF, reflux, 2 h, 35%.

According to path **b** in the retrosynthesis, Scheme 1b, a route was designed for the synthesis of **P2** and **Zn–P2** using **24** as a key synthetic intermediate, as shown in Scheme 4. The synthetic route started by the formylation of pyrrole with POCl_3_/DMF in 1,2-dichloroethane^58^ to yield *1H*-pyrrole-2-carbaldehyde (**15**) in 90% yield. Pyrrole **15** was treated with NBS in THF at −78 °C to afford **16**.^59^ Compound **16** was subsequently treated with dimethylamine (Me_2_NH) (40 wt % in H_2_O) at room temperature for 3.5 h^60^ to give the pyrrole dimer **17** with a 70% yield over 2 steps from **15**. The pyrrole dimer **17** was treated with tertiary-butyllithium (*t*-BuLi) at −78 °C to form the dilithiated species, which was reacted with MeSSMe to generate the pyrrole dimer **18** in 73% yield. Hydrolysis of **18** at reflux in a basic solution (sodium bicarbonate (NaHCO_3_) (aq.)) resulted in **19** in 99% yield. Treatment of **19** with NaH in THF at 0 °C for 1 h, followed by the addition of MsCl, resulted in the protected pyrrole **20** in 76% yield. Compound **20** was reduced by NaBH_4_ in mixed solvents (MeOH/THF) to produce pyrrole carbinol **21** in 98% yield.

The synthesis of dipyrromethane **22** was optimized by employing different kinds of acids in the coupling of **21** and pyrrole (Table S2). The results of the optimization indicated that the reactivity of **21** was totally different from **7** due to the difference in the position of the SMe group. Obviously, the SMe group had a large influence on the reactivity of the *N*-mesylpyrrole carbinols in this condensation reaction. Rewardingly, we found a 70% yield of dipyrromethane **22** when BF_3_·MeOH was employed as a Lewis acid in the condensation (Table S2, entry 4).

**Scheme 5:**
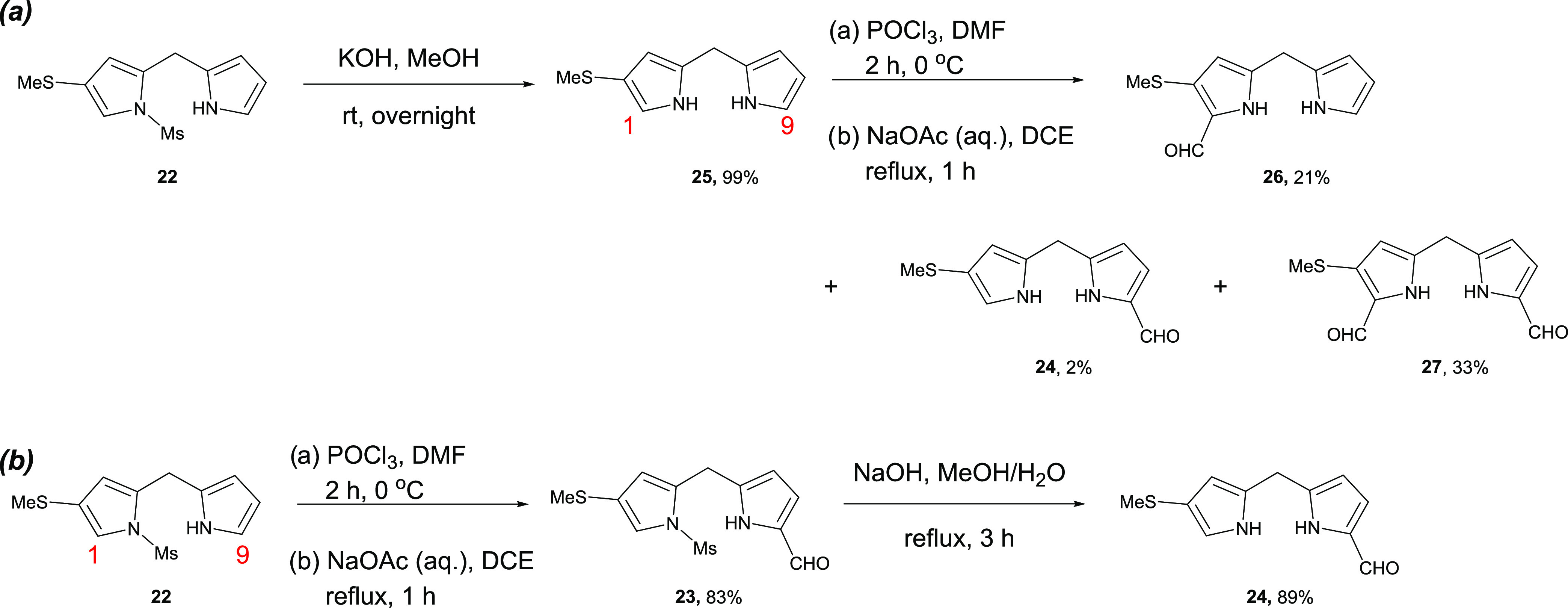
Two Pathways for the Synthesis of the Dipyrromethane **24**

We next attempted to synthesize dipyrromethane **24** using two pathways; see Scheme 5. The Ms deprotection of **22** afforded **25** in 99% yield; see Scheme 5a. The following formylation produced **24** (Scheme 1b) in 2% yield, along with two side products **26** and **27**, in 21% and 33% yields, respectively. This result indicates that the 1-position of **25** is relatively more nucleophilic than the 9-position, in accordance with the theory in which the EDG SMe makes the adjacent 1-position more nucleophilic toward the Vilsmeier–Haack reagent. To improve the yield of **24**, the aldehyde group was first introduced in **22** to form **23** in 83% yield, since the electron-withdrawing effect of the Ms group was supposed to decrease the reactivity of the 1-position in dipyrromethane **22** (Scheme 5b). Instead of using KOH/MeOH in the deprotection of **22**, dipyrromethane **23** was treated with NaOH (5M) in MeOH/H_2_O to obtain **24** in 89% yield.

For the formation of the porphyrin framework, dipyrromethane **24** was treated with MgBr_2_/DBU in toluene at 115 °C for 19 h to afford magnesium porphyrin complex **Mg–P2** in 70% yield (Scheme 4). The tetrapyrrole intermediate was oxidized in situ under aerobic conditions. In this reaction, in order to activate the carbonyl group of **24**, MgBr_2_ was employed to activate the oxygen of the formyl group of **24** due to the high affinity of magnesium(II) for oxygen.^32^ Mg (II) also helped preorganize the dipyrromethane for its self-condensation. The high yield of the formed **Mg–P2** indicates that the 9-position of **24** is more reactive than the same position of **14**. **Mg–P2** was treated with TFA in CH_2_Cl_2_ at 0 °C for 20 min, then followed neutralization by NaHCO_3_ (sat. aq.) to afford free-base porphyrin **P2** in 50% yield. Metalloporphyrin **Zn–P2** was obtained using **P2** and zinc acetate dihydrate in THF under reflux for 2 h in a 35% yield. As a result, **Zn–P2** was synthesized in 13 steps from pyrrole in 2.1% yield. The full characterizations of all compounds, including UV–vis spectroscopy, are found in the Supporting Information (SI).

### Measurements of Electrical Conductance and Thermopower of Porphyrin Molecular Junctions

The electrical conductance and thermopower of molecular junctions created from the synthesized free-base porphyrin and zinc porphyrin molecules were experimentally investigated by using a scanning tunneling microscope-based break-junction (STM-BJ) technique.

**Figure 4:**
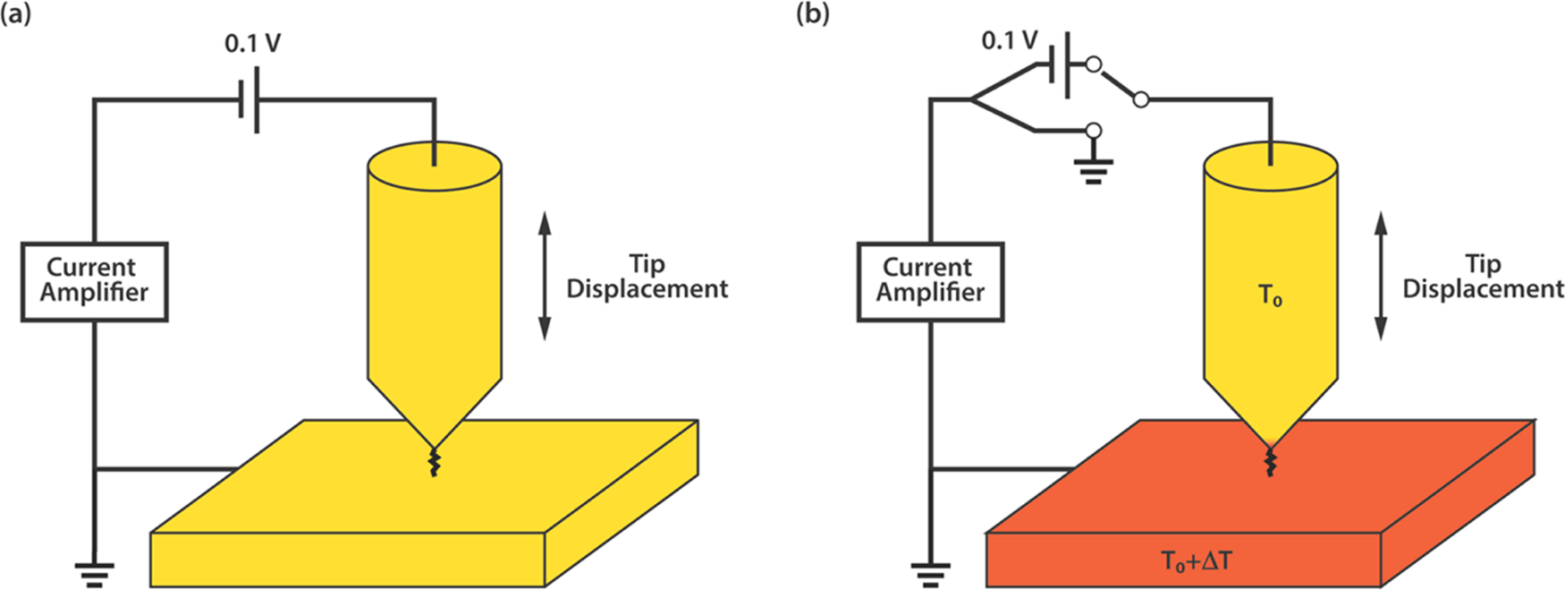
Schematic description of electrical conductance and thermopower measurements. (a) In the electrical conductance measurements, the tip voltage bias was kept at 0.1 V, and the substrate was grounded. (b) In the thermopower measurements, the tip voltage bias was periodically switched between 0.1 and 0 V, while the temperature of the substrate was increased to establish a temperature differential across the junction. In both measurements, the electric current flowing between tip and substrate was recorded by using a current amplifier. All measurements were performed under ambient conditions.

**Figure 5:**
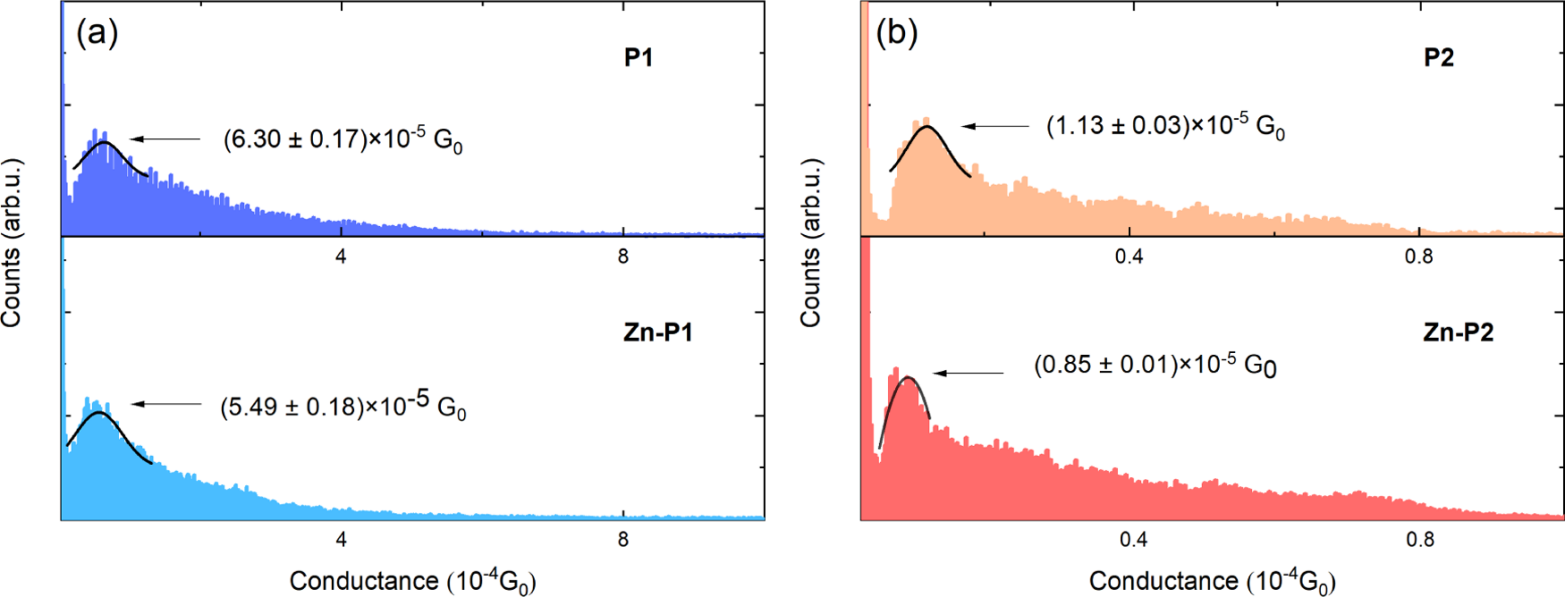
Electrical conductance histograms of (a) **P1** and **Zn–P1**, and (b) **P2** and **Zn–P2** were constructed from over 1000 traces each without any data selection. All of the histograms were fitted using a Gaussian distribution, and each peak represents the most probable electrical conductance of the corresponding single-molecule junction. During the electrical conductance measurements, the Au tip was at 0.1 V bias, and the Au substrate was grounded all the time.

Our STM-BJ measurements are based on forming molecular junctions (MJs) between an electrochemically etched gold tip and a freshly prepared, template-stripped Au substrate (see Figure 4 and the SI for details). First, a molecular layer of the porphyrin molecule under study was formed by the drop-casting method (see the SI for details of the molecular layer formation) on the Au substrate. Then, a 0.1 V bias was applied between the tip and the grounded substrate, as shown schematically in Figure 4a, while the electric current between the tip and gold substrate was simultaneously monitored. To start an actual measurement, the tip was slowly advanced toward the substrate until it contacted the Au surface, as indicated by a rapid current increase that results from the formation of the tip–substrate contact. Then, the tip was retracted from the substrate at a speed of 0.2–0.4 nm/s, while the tunneling current between the tip and the substrate was simultaneously recorded (see the SI for details of measurements) until the tip–substrate junction broke. In this slow retraction process, a few molecules from the assembled molecular layer were stochastically trapped between gold atoms of the tip and those of the substrate, and as the tip was further retracted, the remaining molecular junctions between the gold electrodes broke in a stepwise manner that resulted in transient plateaus in the recorded currents and clear peaks in the corresponding electrical conductance histograms. Such electrical conductance measurements were performed for all four molecules, and conductance histograms were constructed for each molecular species from around 1000 conductance-distance traces, which were collected and concatenated from more than two samples for each molecule. Histograms obtained from such measurements are shown in Figure 5, along with a Gaussian fit to determine the most probable conductance value of these molecules (see the SI for two-dimensional (2D) density plots generated from the raw data of the same conductance traces).

The histogram in Figure 5a shows that the free-base porphyrin Au-**(P1**)-Au SMJ has a Gaussian-fitted peak with the most probable conductance located at around 6.30 × 10^–5^*G*_0_ (where *G*_0_ = 2*e*^2^/*h* is the quantum of conductance). With the introduction of a Zn ion, the conductance peak of Au-(**Zn–P1**)-Au SMJ shifts to a slightly smaller value of 5.49 × 10^–5^*G*_0_. Similarly, the addition of the Zn ion leads to a slightly lower electrical conductance of 0.85 × 10^–5^*G*_0_ (Au-(**Zn–P2**)-Au SMJ) compared to the corresponding free-base porphyrin Au-**P2**-Au SMJ, the electrical conductance of which is around 1.13 × 10^–5^*G*_0_ (see also Table 1). The electrical conductance can thus be tuned substantially by altering the attachment points of the SMe anchoring groups on the porphyrin skeleton, resulting in changing the skeleton’s geometric symmetry from *C*_2v_ (**P1**) to *C*_2h_ (**P2**). In comparison, the introduction of a Zn ion has only a small influence on the electrical conductance, close to the uncertainty of the experiment. For **P2** vs **Zn–P2**, it results in a 25% reduction in electrical conductance, which is more significant than the 13% reduction between **P1** and **Zn–P1**. Overall, these results indicate that altering the relative positions of the anchor groups on the β-core of a porphyrin and adding a centrally located metal ion can tune the electrical conductance of molecular junctions. However, the conductance of the β-core-based single-molecular junction is lower compared to a porphyrin substituted in the *meso*-position (5,15) with a (4-thiophenyl)acetylenyl anchor group to the gold surface, in both the free-base porphyrin and the zinc complex, whose electrical conductance is 2.8 × 10^–4^*G*_0_ and 2.4 × 10^–4^*G*_0_, respectively.^61^

**Table 1:** Experimentally Determined Single-Molecule Electrical Conductance, Thermopower, and Power Factors of Au-**P1**-Au, Au-(**Zn−P1**)-Au, Au-**P2**-Au, and Au-(**Zn–P2**)-Au, Connected to Gold Electrodes at Room Temperature

molecule	conductance, *G* (*G*_0_)	thermopower, *S* (μV/K)	power factor, *S*^2^*G* (W/K^2^)
**P1**	(6.30 ± 0.17) × 10^–5^	(9.02 ± 0.16)	∼3.97 × 10^–19^
**P2**	(1.13 ± 0.03) × 10^–5^	(7.51 ± 0.59)	∼4.94 × 10^–20^
**Zn–P1**	(5.49 ± 0.18) × 10^–5^	(13.54 ± 0.97)	∼7.80 × 10^–19^
**Zn–P2**	(0.85 ± 0.01) × 10^–5^	(2.92 ± 0.12)	∼5.62 × 10^–21^

In order to measure the thermoelectric properties of molecular junctions created from porphyrin molecules, we first established stable temperature differentials (Δ*T*) of 0 K, 5.7 K, 9 K, and 11.4 K between the tip and the substrate (see the SI for details of how the temperature differentials were established). As schematically shown in Figure 4b, the base of the tip was maintained at room temperature (∼300 K), and the substrate was heated to the desired temperature with an integrated film heater. Similar to the approach taken in the electrical conductance measurements, the Au substrate was grounded, and a 0.1 V direct current (DC) bias was applied to the Au tip. Subsequently, molecular junctions were created using a variation of the approach described above. During the tip retraction process, when the measured conductance (*G*) was within ±50% of the most probable conductance, the tip movement was immediately paused, and the bias applied to the tip was periodically switched between 0.1 and 0 V with a periodicity of 0.4 s (see the SI for sample traces from such a measurement). In these experiments, the recorded current at 0.1 V bias served to identify whether the junction was broken or not, while the current flowing through the junction for 0 V bias represented the short-circuit thermoelectric current (*I*_th_) generated by the junction that could be related to the open circuit thermoelectric voltage (Δ*V*_TE_, see the SI for details of short circuit and open circuit for thermoelectric measurement) via
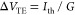
1The process of alternating the applied bias between 0.1 and 0 V was continued until the measured conductance at a bias voltage of 0.1 V dropped to its noise level, indicating that no molecular junctions existed between the tip and the substrate. When this condition was met, suggesting that the molecular junction was broken, the tip was completely withdrawn, and the process of trapping a molecule and executing the measurements was repeated. For each temperature differential Δ*T*, we collected the thermoelectric voltage signal from many independent measurement cycles (around 5000 for each temperature differential), which was used to build histograms.

**Figure 6:**
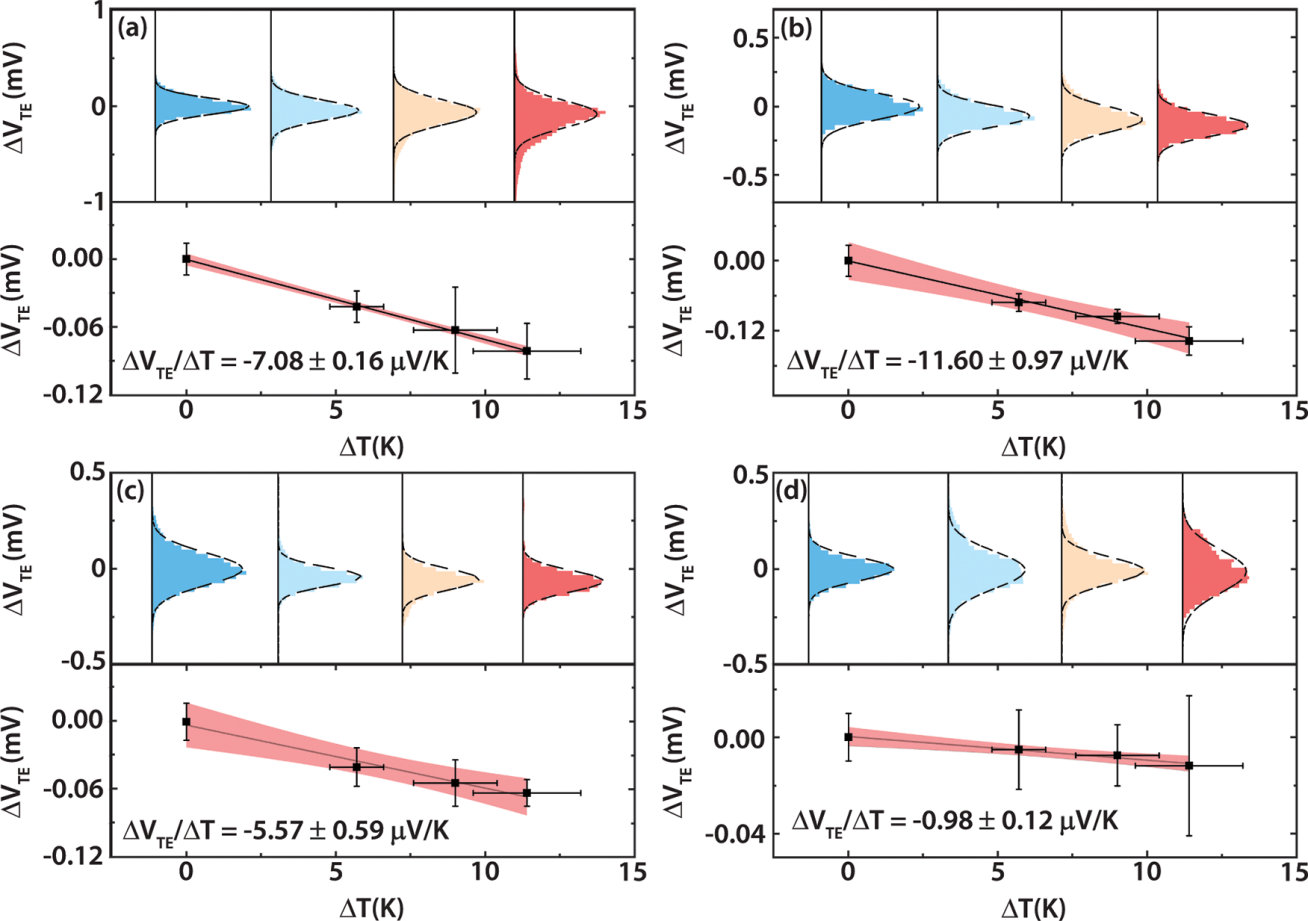
Thermoelectricity measurements of single-molecule junctions with Au-**P1**-Au (a), Au-(**Zn–P1**)-Au (b), Au-**P2**-Au (c), and Au-(**Zn–P2**)-Au (d), respectively: (upper panel) thermoelectric voltage histograms of molecular junctions at temperature differentials of 0, 5.7, 9, and 11.4 K, (lower panel) thermoelectric voltage vs temperature differential, and slope (Δ*V*_TE_/Δ*T)* fitting results. Each histogram was constructed from around 5000 independently measured traces without any data selection and was fitted by a Gaussian distribution. The peak of the Gaussian distribution represents the most probable thermoelectric voltage generated by molecular junctions subjected to the corresponding temperature differential.

The top panels of Figure 6 show the thermoelectric voltage histograms corresponding to **P1**, **Zn–P1**, **P2**, and **Zn–P2** molecular junctions at various temperature differentials. Further, we show Gaussian fits to the measured histograms, whose peaks correspond to the most probable thermoelectric voltage at each temperature differential. In each figure, the lower panel plots the temperature-dependent thermoelectric voltage (as identified from the peak of the histogram) and a corresponding linear fit to the data, whose slope, Δ*V*_TE_/Δ*T*, is related to the thermopower of the molecular junction.

We note that the sign of the thermoelectric voltage is considered positive when current flows from the tip to the substrate through the junction and negative when current flows in the opposite direction (see the SI for details). The thermopower (i.e., Seebeck coefficient) of a molecular junction (*S*_junction_) is obtained from (see the SI for details)
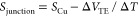
2where *S*_Cu_ = 1.94 μV/K represents the thermopower of bulk copper at room temperature (300 K) and is included to account for a copper wire that is used in the experimental configuration employed in this work (see the SI), and Δ*V*_TE_/Δ*T* is obtained from the linear fitting shown in the lower panels of Figure 6. From this analysis, the room-temperature thermopowers of the four different molecular junctions are calculated by using eq 2 and are reported in Table 2, along with the power factors. The experimental results indicate that the introduction of a central Zn ion can either increase or decrease the thermopower of the porphyrin molecular junctions. For the **P1** system, the introduction of a Zn ion improves the thermopower by ∼50%, while introducing a Zn ion into the **P2** system suppresses the thermopower by 61%. The comparatively smaller electrical conductance suppression (12.8% from **P1** to **Zn–P1** and 24.8% from **P2** to **Zn–P2**) shows that the introduction of a Zn ion into the porphyrin molecular system has a more significant effect on thermopower than on electrical conductance. Interestingly, our experimental observations also suggest that the thermopower is less susceptible to anchor attachment positions than the electrical conductance. Specifically, we observe an electrical conductance reduction of over 500% from **P1** to **P2**, while the thermopower suppression is only changed by 16.7%. However, in the case of the two Zn porphyrins, both the electrical conductance and the thermopower are more sensitive to anchor points. The electrical conductance reduced by more than 6-fold, while the thermopower is suppressed ∼5-fold from **Zn–P1** to **Zn–P2**. The record thermopower measured in this study, 13.54 ± 0.97 μV/K for a Au-**P1**-Au molecular junction, is of the same magnitude as recently reported by us for an Au-OPE-Au molecular junction, ∼20 μV/K (OPE = oligo (phenylene ethynylene)).^62^

**Table 2:** Calculated Values for HOMO–LUMO Gaps of the Isolated Molecules **P1**, **P2**, **Zn–P1**, and **Zn–P2** Using DFT, Δ*S*CF, and *G*_0_*W*_0_^a^

molecule	DFT (eV)	ΔSCF (eV)	*G*_0_*W*_0_ (eV)
**P1 (|)**	1.88	5.22	4.71
**P1 (−)**	1.89	5.19	4.65
**P2 (|)**	1.87	5.08	4.75
**P2 (−)**	1.88	5.16	4.65
**Zn–P1**	2.01	5.31	4.79
**Zn–P2**	2.01	5.25	4.80

a For DFT, we use the PBE exchange–correlation functional.^72^ All electronic structure methods employ the def-TZVP basis set^73^ and the geometries determined with DFT.

### Calculations of the Electrical Conductance and the Thermopower of Porphyrin Molecular Junctions

In order to obtain further insights into the experimental observations, we computationally explored the effect of the SMe anchor positions and the addition of a central Zn ion on the thermoelectric properties of the molecular junctions. For this purpose, we describe the quantum transport properties of molecular junctions in linear response theory based on the Landauer–Büttiker scattering approach.^63^ In this formulation, the electronic transmission τ(*E*) determines both the conductance
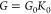
3and the thermopower of the junction
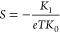
4Here
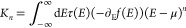
5and we approximate the chemical potential μ by the Fermi energy *E*_F_ = −5 eV and set the temperature to *T* = 300 K. We express the transmission in terms of nonequilibrium Green’s functions and obtain the information on the electronic structure needed to compute the transmission function from density functional theory (DFT).^63,64^ All electronic structure calculations were performed with the program package TURBOMOLE,^65^ and the transmission function was evaluated within custom codes.^64^

**Figure 7:**
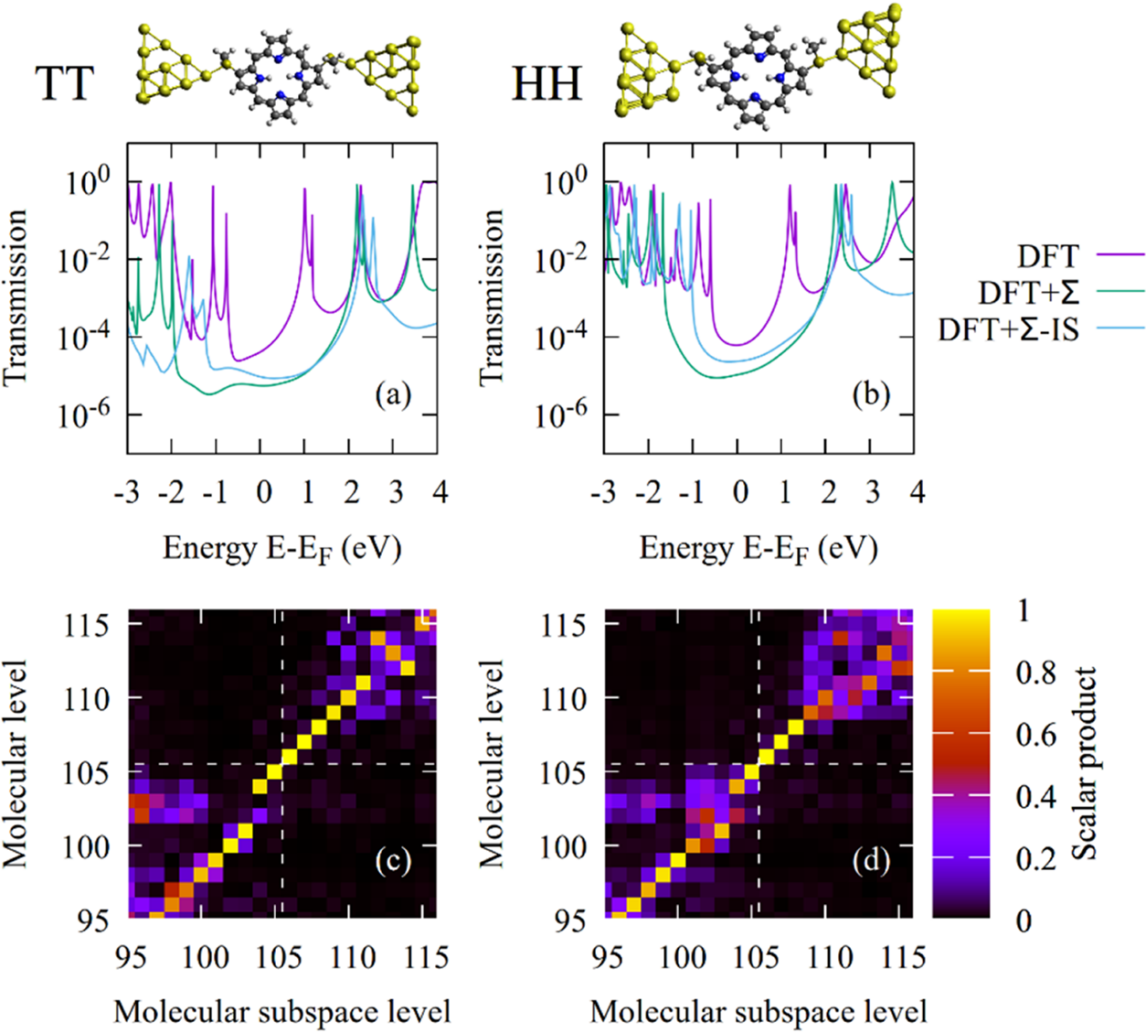
(a, b) Transmission curves for Au-**P1(−)**-Au junctions with the DFT+Σ-IS method in HH and TT junction geometries, using individual shifts that are determined according to a mapping analysis. For comparison, the DFT and DFT+Σ transmission curves are shown as well. Junction geometries are displayed at the top of the figure. (c, d) Mapping between the eigenstates of the molecular subspace in the corresponding single-molecule junction and the molecular orbitals of the isolated molecule **P1(−)** in TT and HH geometries around the HOMO (index 105) and LUMO (index 106), separated by the dashed white line. For TT, the absolute value of the scalar product is above the matching threshold for HOMO – 5 to LUMO + 5. For HH, this condition is fulfilled for HOMO to LUMO + 2.

To analyze charge transport through the four different porphyrins, **P1**, **Zn–P1**, **P2**, and **Zn–P2**, we use two different junction types. In the first one, the tips are atomically sharp, and the sulfur atoms are arranged such that they bind to a single Au tip atom (see Figure 7). We refer to this geometry as top–top (TT). The second junction type, which we call hollow–hollow (HH), is obtained by removing the Au tip atom on each side. The SMe anchors then bind to a single Au atom on the blunt tips (Figure 7). While outer atoms of the extended central cluster, consisting of the metal-molecule-metal junctions, are fixed, the molecule and the atoms next to it are fully optimized in the DFT calculations. Thus, for the metal part in the TT geometry, the Au tip atom and the three Au atoms following it on each side are energetically optimized; for the HH geometry, this is only true for the three Au atoms of the blunt tip of each electrode fragment. The unsubstituted porphyrin molecules **P1** and **P2** show a tautomerism, as they feature two hydrogen atoms inside the porphyrin ring, bound to two opposing nitrogen atoms. With anchoring SMe groups attached, the two opposed nitrogen atoms that carry the hydrogen atoms can be arranged next to the anchoring groups (parallel) or in the center line between the SMe groups (perpendicular). This leads to two different geometries, both in the respective free-base porphyrins and in the molecular junctions, which we indicate by (−) and (|)^66^ (compare Figures 7 and S37–S39 of the SI).

The DFT method is known to underestimate the electronic gaps of isolated molecules. Approaches to overcome this problem are, e.g., the ΔSCF approach^67,68^ that is based on total energy differences between neutral and singly charged molecular species as well as the *GW* approximation, which is founded on many-body perturbation theory (MBPT) and Hedin’s equations.^69^ In Table 2, we show the calculated electronic gaps between the HOMO and the LUMO for the different molecules obtained using DFT, Δ*S*CF, and the nonself-consistent *G*_0_*W*_0._(70,71) Within each method, we find that gap sizes of **P1** and **P2** are very similar. Replacement of the two hydrogen atoms by a Zn atom in the center of the molecules has only a small effect on the HOMO–LUMO gaps in this case, which increase by around 0.1 eV. Further technical details on the calculations and discussions can be found in the SI.

The electronic gap size problem of DFT, discussed above, affects quantum transport calculations. Since the molecules tend to exhibit too small HOMO–LUMO gaps, theoretically determined conductance values are typically overestimated. As is visible from the data presented for DFT in the SI, we indeed find a slight overestimation of theoretical conductance values. While results for the HH and TT junctions generally agree quite well with the experiment, **Zn–P1** in the TT geometry shows a pronouncedly overestimated conductance. In agreement with the experiment, conductances of **P1**-derived junctions are larger than those for **P2**-derived ones. A slight increase of conductance upon replacement of hydrogens by Zn in the center of the porphyrins is predicted, opposite to the experimentally observed moderate reduction. Except for **Zn–P2**, the thermopower values of the molecular junctions tend to be too low and often negative instead of positive, in contrast to the average values in the experimental histograms. These computational results emphasize the need for improving upon the predictions of purely DFT-derived quantum transport properties.

A common way to improve upon the DFT results is the DFT + Σ correction scheme,^67,68^ which consists of two parts, namely, a “molecular” term, correcting energies of the isolated molecule, and an “image-charge” term, accounting for the effect of electrode polarizations. Within this approach, all energy levels of the molecular subspace in the extended central cluster are adjusted before the transmission is calculated. Specifically, the occupied levels are shifted by Σ_occ_ and the virtual (or unoccupied) levels by Σ_vrt_.
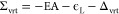
6
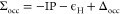
7

In the expressions, EA and IP are the electron affinity and ionization potential, extracted from ΔSCF calculations for the isolated molecule, and ϵ_H_ and ϵ_L_ are the DFT energies of the HOMO and LUMO of the isolated molecule, respectively. The shifts Δ_occ_ and Δ_vrt_ describe the so-called image-charge corrections, which depend on the charge distributions of the corresponding molecular orbitals of the molecules and the location of assumed infinitely extended, ideal electrode metal surfaces. The shifts Σ_occ_ and Σ_vrt_ are typically calculated only for HOMO and LUMO and then applied to all occupied and virtual states. The transmission functions for the molecules **P1**, **P2**, **Zn–P1**, and **Zn–P2** and the resulting values for conductance and thermopower for the DFT+Σ approach are shown in the SI (Figure S36). Except for **Zn–P1** and **Zn–P2** in the TT geometry, the transmission curves of the different studied molecules resemble each other. We find a higher conductance for **P1** compared to **P2** and for **Zn–P1** compared to **Zn–P2** for both binding geometries in agreement with the experimental results shown in Table 1. The small decrease of the conductance of the Zn-substituted molecules **Zn–P1** and **Zn–P2** compared to **P1** and **P2**, respectively, that was found in experiments, is partly reproduced but depends on the studied isomer (**P1(|)** vs **P1(−)** or **P2(|)** vs **P2(−)**) and junction geometry. As compared to experiments, absolute conductance values are underestimated. The thermopower values calculated based on the DFT + Σ method do not agree well with the experimental findings. Similar to DFT, they tend to be negative and too small.

To further improve the results, we present a new correction scheme in the following that refines the DFT + Σ results. As explained above, all occupied and unoccupied orbitals are shifted by the same Σ_occ_ and Σ_vrt_ values in the commonly applied variant of the DFT + Σ scheme. Instead, we aim at calculating individual shifts for each orbital. The determination of the image-charge corrections Δ^*i*^ can easily be extended to individual orbitals, enumerated by the index *i*. For the correction of DFT molecular orbital energies of the isolated molecule, ϵ_GW_^*i*^ – ϵ_DFT_^*i*^, we use *G*_0_*W*_0_ calculations^66,67^ of the gas-phase molecule. Hence, the correction for each individual orbital *i* reads
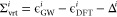
8
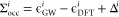
9To apply these shifts, we need a mapping between the orbitals of the isolated molecule and the eigenstates in the molecular subspace of the molecular junction. We accomplish this by computing the scalar product between these two groups of orbitals and by searching for the best agreement (see the SI for further details). Since a unique mapping is essential for the quality of the presented method, the absolute value of the resulting scalar product is shown as a two-dimensional map plot in Figure 7. Figure 7a,b displays the resulting transmissions for DFT, DFT+Σ, and the newly introduced method for TT and HH junctions and molecule **P1(−)**. We will henceforth call the newly developed method DFT + Σ-IS, where IS stands for individual shift of orbitals *i*. Figure 7c,d presents the corresponding 2D orbital mappings. To accept the calculated shift, we require the absolute value of the scalar product to exceed a matching threshold value of 0.9. This matching threshold is chosen as a compromise between the desire to achieve the best possible match of orbitals and to correct individually as many orbitals of the molecular subspace as possible. The orbital index of the HOMO of the molecular subspace for molecule **P1** is 105. In the case of the TT geometry, we find that all orbitals from HOMO to HOMO – 5 fulfill the matching condition, while the HOMO – 6 is below. Thus, we accept the individual shifts for the orbitals HOMO to HOMO – 5 and then assign the shift of the HOMO – 5 to all orbitals below the HOMO – 5. Similarly, we accept the individual shifts for all virtual orbitals from LUMO to LUMO + 5, where the matching threshold is fulfilled, and assign the shift of LUMO + 5 to all other high-lying virtual orbitals starting from LUMO + 6, where the matching condition is first violated. The transmission curves in Figure 7a,b show how the gap is opened when transitioning from DFT to DFT + Σ or DFT + Σ-IS. Due to the more off-resonant transport situation, the conductance drops. The comparison of DFT + Σ and DFT + Σ-IS shows that LUMO resonances are located at similar energies. But the HOMO states are positioned higher in energy for DFT + Σ-IS than for DFT + Σ. As a result, transport will be more strongly hole-dominated, and thermopowers should increase.

**Figure 8:**
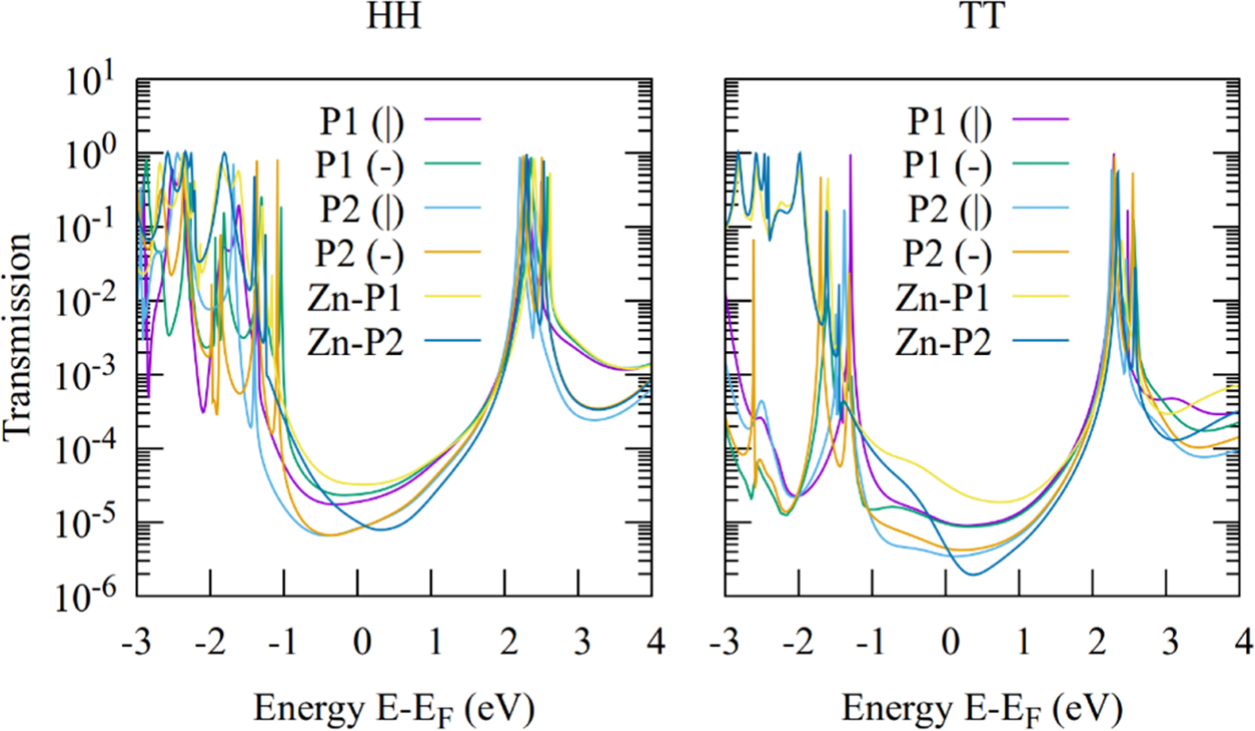
Transmission curves of **P1**, **P2**, **Zn–P1**, and **Zn–P2** in HH and TT geometries using DFT + Σ-IS calculations, which involve individual shifts based on *G*_0_*W*_0_ calculations and image-charge corrections for the molecular subspace.

Resulting transmission curves for all studied molecular junctions with the method DFT + Σ-IS are displayed in Figure 8, and Table 3 lists the computed conductance values and Seebeck coefficients. Similar to DFT + Σ, conductance values calculated with DFT + Σ-IS generally underestimate experimental values but reproduce important trends such as the reduction in conductance when going from **P1** to **P2** and similarly from **Zn–P1** to **Zn–P2**. The slight decrease of conductance upon introduction of the Zn atom is typically not reproduced, but a slight increase is predicted. Due to the more strongly HOMO-dominated transport in the DFT + Σ-IS approximation, the thermopower values are increased as compared to DFT + Σ. While they tend to be too low in the HH geometry, the TT geometry yields positive values in reasonable agreement with the measurements. A drop of the thermopower from **P1** to **P2** is reproduced, and the rather high value is for **Zn–P1**. We note, however, that the thermopower for **Zn–P2** is predicted to be the highest by the newly developed method in both TT and HH junctions, a feature that does not agree with the experiment.

**Table 3:** Calculated Values for Conductance *G* and Thermopower *S* of **P1**, **P2**, **Zn–P1**, and **Zn–P2** Using the Maximum Range of Individual Shifts in the DFT + Σ-IS Method

molecule	*G* (*G*_0_) [HH]	S (μV/K) [HH]	*G* (*G*_0_) [TT]	*S* (μV/K) [TT]
**P1(|)**	1.88 × 10^–5^	–2.78	9.99 × 10^–6^	5.48
**P1(−)**	2.38 × 10^–5^	–1.62	9.57 × 10^–6^	4.59
**P2(|)**	8.23 × 10^–6^	–5.62	3.53 × 10^–6^	2.61
**P2 (−)**	8.12 × 10^–6^	–5.72	4.46 × 10^–6^	4.07
**Zn–P1**	3.25 × 10^–5^	0.70	3.30 × 10^–5^	11.46
**Zn–P2**	1.02 × 10^–5^	10.14	4.78 × 10^–6^	30.21

We conclude that while basic electrical conductance trends for the studied Au-porphyrin-Au single-molecule junctions are reproduced with the applied quantum transport schemes, trends in thermopower measurements appear to be more complicated to predict. This may be related to the rather moderate experimental variations within a factor of at most 4 or the dependence on the slope of the transmission, which is arguably more challenging to describe. Finally, despite our efforts to treat the electronic structure for the quantum transport calculations accurately, it may well be that the theoretically assumed HH and TT junction geometries are not the ones typically realized in the experiment.

Previous theoretical work^16^ predicted highly efficient thermoelectric devices based on two-level coherent transport models. The porphyrin **Zn–P1** was suggested as a possible realization and inspired this work. We thus hoped for a big difference in conductance and thermopower values between the **Zn–P1** and **Zn–P2** derivatives, which is not supported by our experiments or detailed computational analysis. In the isolated porphyrin molecule without SMe anchors, HOMO – 1 and HOMO levels are energetically nearly degenerate. For molecule **Zn–P2**, the wave functions of HOMO and HOMO – 1 have the same parity on the carbon atoms attached to the SMe anchoring groups but a different parity for molecule **Zn–P1**. According to ref (16), this may lead to a constructive quantum interference in **Zn–P2** and a destructive quantum interference in **Zn–P1**, with a hopefully extraordinarily high thermopower for **Zn–P1**. In the SI, we discuss in detail why this high thermopower is not observed. In short, the molecules **Zn–P1** and **Zn–P2**, similar to **P1** and **P2**, turn out to be no good realization of the two-level model proposed in ref (16), since more molecular orbitals than just the HOMO and HOMO – 1 contribute crucially to the conductance and thermopower. For this reason, the quantum transport properties of **Zn–P1** and **Zn–P2** single-molecule junctions are quite similar.

## Conclusions

We studied both experimentally and theoretically the electrical conductance and thermopower of molecular junctions created from two classes of porphyrins attached to gold electrodes at their β-position. Our work is novel in various main aspects: (i) we successfully developed the methodology to synthesize 2,13- and 2,12-disubstituted porphyrins, of which only one example exists to date,^51^ (ii) we experimentally studied charge transport in molecular junctions, created by attaching for the first time porphyrins to gold electrodes via their β-positions, (iii) we measured the thermopower of porphyrin molecular junctions for the first time, as well as (iv) we developed and applied advanced quantum transport theory in attempts to explain the experimental results using first principles. Our single-molecule break-junction measurements show that the electrical conductance of the **P1** and **Zn–P1** junctions is very similar, and the same holds for **P2** and **Zn–P2**, while there is a 6-fold reduction in the conductance of **P2** type junctions relative to the **P1** type. Thus, the position of the anchoring groups has a stronger effect on conductance than zinc insertion. Similarly, the thermopower for **P1** and **Zn–P1** junctions yields higher values than for **P2** and **Zn–P2** junctions. We also observe that the thermopower of **P1** junctions is slightly larger than that for **P2** junctions, while **Zn–P1** junctions show the largest thermopower and **Zn–P2** junctions the lowest, indicating that a reduced electrical conductance does not always correlate with an increased thermopower.

Hence, from these findings, we deduce that the electrical conductance and thermopower can be tuned by modifying the methylthio attachment points on the β-positions of the porphyrin skeleton (change in geometric symmetry from *C*_2v_ (**P1**, **Zn–P1**) to *C*_2h_ (**P2**, **Zn–P2**)). The measured conductance is somewhat lower compared to what is observed for a published porphyrin molecular junction with attachment points of the thiogroup to the gold surface in the 5,15-*meso*-position (both as free base and with zinc inserted).^61^ The thermopower is similar to that reported in our work concerning OPE molecular junctions.^62^

In order to understand the transport mechanisms in these molecular junctions, we applied different levels of electronic structure descriptions in quantum transport schemes to go beyond the predictions of pure DFT. Basic trends of a reduced electrical conductance for the transition from **P1** to **P2** derivatives are reproduced with all procedures; the newly introduced DFT + Σ-IS scheme improves upon the conventional DFT + Σ approach in terms of somewhat larger conductance and thermopower values in better agreement with the experiment.

Finally, in our experiments and theory, we did not observe the predicted^16^ large difference in conductance and thermopower values between **Zn–P1** and **Zn–P2** derivatives. Our work highlights the need for further refinements to current electronic structure methods to achieve better quantitative agreement of experimental and theoretical quantum transport properties, ultimately enabling an accurate prediction of synthesis targets for high-performing thermoelectric molecular structures. In this context, it will be crucial to obtain better atomistic knowledge of frequently realized experimental junction geometries and the junctions’ chemical environment to calibrate the theoretical procedures. We are confident that our pioneering study of the thermopower in porphyrin molecular junctions will provide a reference for future computational and experimental endeavors. We note that the experimental approach employed here does not directly enable the control of the position of the energy levels with respect to the electrochemical potential of the electrodes. Future experimental studies of thermoelectrics in molecular junctions will benefit from the use of gate electrodes^4,74−77^ to better understand and control level alignments and to enable tuning to an optimal combination of high thermopower and high thermocurrent.^78^

## Supporting Information Available

Synthetic protocols and synthesis characterization data of all of the new chemical compounds; detailed experimental techniques for electrical conductance and thermopower measurements; details about theoretical calculations using DFT; and theoretical studies of quantum interference in Zn porphyrin molecules and of transmission eigenchannels (PDF)
