# Supplementary material for: Electrical Conductance and Thermopower of β-Substituted Porphyrin Molecular Junctions—Synthesis and Transport
Source: J Am Chem Soc. 2023 Oct 24;145(43):23541–55. doi: 10.1021/jacs.3c07258 (PMC10623571; doi:10.1021/jacs.3c07258)
Supplement: Supplementary file 1 — ja3c07258_si_001.pdf [file ja3c07258_si_001.pdf]

Supporting Information

Electrical Conductance and Thermopower of *β*-Substituted Porphyrin Molecular Junctions – Synthesis and Transport

Hailiang Xu,[1,2§] Hao Fan,[2§] Yuxuan Luan,[3§] Shen Yan,[3§]
León Martin,[4§] Ruijiao Miao,[3] Fabian Pauly,[4*] Edgar Meyhofer,[3*] Pramod Reddy,[3,5*] Heiner Linke[1,6*] and Kenneth Wärnmark[1,2*]

[1] NanoLund, Lund University, Box 118, 22100 Lund, Sweden
[2] Department of Chemistry, Centre of Analysis and Synthesis, Lund University, Box 121, 22100 Lund, Sweden
[3] Department of Mechanical Engineering, University of Michigan, Ann Arbor, MI 48109, United States
[4]Institute of Physics and Centre for Advanced Analytics and Predictive Sciences, University of Augsburg, 86159 Augsburg, Germany
[5] Department of Materials Science and Engineering, University of Michigan, Ann Arbor, MI 48109, United States

[6] Solid State Physics, Lund University, Box 118, 22100 Lund, Sweden

Corresponding Authors

* Fabian Pauly: E-mail: fabian.pauly@uni-a.de

* Edgar Meyhofer: E-mail: meyhofer@umich.edu

* Pramod Reddy: E-mail: pramodr@umich.edu

* Heiner Linke: E-mail: heiner.linke@ftf.lth.se

* Kenneth Wärnmark. E-mail: kenneth.warnmark@chem.lu.se

Author Contributions

§These authors contributed equally to this work.

Table of Contents

Part 1. General Information 2

Part 2. Details of Optimizations and Synthesis. 3

Part 3. ^{1}H and ^{13}C NMR Spectra for New Compounds........ 15

Part 4. Electronic structure of isolated molecules........ 40

Part 5. Experimental techniques........ 44

Part 6. Transport calculations using DFT 51
Part 7. Transport calculations using DFT+Σ 53
Part 8. Transport calculations using DFT+Σ-IS 54
Part 9. Quantum interference in Zn-porphyrin molecules 61
Part 10. Transmission eigenchannels 63
References 66

Part 1. General Information

Reactions run at room temperature in the range of 20-23 °C. Anhydrous THF was distilled from sodium/benzophenone ketyl prior to use. All other solvents were ACS grade. The following reagents, pyrrole, *n*-butyllithium solution (*n*-BuLi, 2.5 M in hexane), triisopropylsilyl chloride (TIPSCl), *N*-bromosuccinimide (NBS), dimethyl disulfide (MeSSMe), tetrabutylammonium fluoride (TBAF, 1.0 M in THF), phosphorus (V) oxychloride ($POCl_3$), *N,N*-dimethylformamide (DMF, anhydrous), methanesulfonyl chloride (MsCl), sodium borohydride ($NaBH_4$), DDQ (2,3-dichloro-5,6-dicyano-*p*-benzoquinone), dimethylamine solution (40 wt% in H_2O), *tert*-butyllithium solution (*t*-BuLi, 1.7 M in pentane), boron trifluoride-methanol solution ($BF_3 \cdot MeOH$, 10% in methanol), zinc acetate dihydrate ($Zn(OAc)_2 \cdot 2H_2O$) and 1,8-diazabicyclo[5.4.0]undec-7-ene (DBU) were purchased, and used as received from Sigma Aldrich. *N*-bromosuccinimide (NBS) was purchased from Sigma Aldrich and recrystallized with boiling water before use. Magnesium bromide ($MgBr_2$) was purchased from Sigma Aldrich and dried at 150 °C under vacuum over night before use. The following compounds **1**,[1] **2**,[1] **15**,[S2] **16**,[3] **17**[4] and **11**[5] were synthesized according to the literature. Column chromatography (ø x h, cm) was performed using silica gel (60 Å, 230-300 mesh, purchased from Aldrich) as the solid support. All prepared compounds were transferred from the flasks into the small sample vessels using CH_2Cl_2 as the solvent. All NMR spectra were recorded on a Bruker Avance 400 MHz spectrometer. Deuterated solvents were used as received from Sigma Aldrich. ^1H NMR and ^{13}C NMR chemical shifts are reported in δ units, parts per million (ppm) relative to the chemical shift of the residual solvent; $CDCl_3$ (7.26 ppm for ^1H NMR spectra and 77.16 ppm for ^{13}C NMR spectra), DMSO-d_6 (2.50 ppm for ^1H NMR spectra and 39.52 ppm for ^{13}C NMR spectra), TFA-*d* (11.50 ppm for ^1H NMR spectra and 164.20 ppm for ^{13}C NMR spectra), THF-d_8 (1.73 ppm for ^1H NMR spectra and 67.21 ppm for ^{13}C NMR spectra). The melting point was recorded on a micro melting point apparatus SMP3 (Stuart Scientific, UK) and corrected using standard compounds. IR spectra were recorded on a Bruker IR spectromenter Alpha II. Electron spray ionization–high resolution mass (ESI–HRMS) spectra were recorded on a Waters Micromass Q-Tof micro mass spectrometer. Elemental analyses were performed by Mikroanalytisches Laboratorium KOLBE (Mülheim an der Ruhr, Germany).

Part 2. Details of Optimizations and Synthesis.

Table S1. Optimization for the synthesis of tripyrrane **8**.

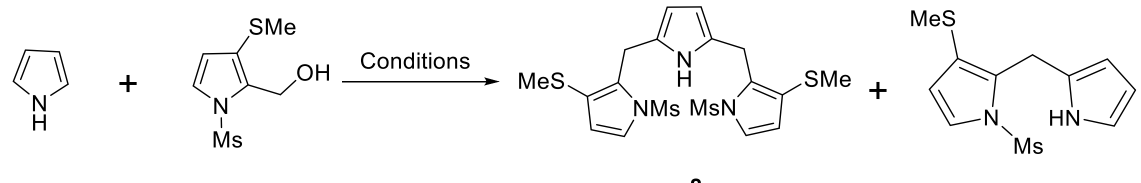

Entry	Pyrrole : 7	Conditions	8 (%)[a]	12 (%)[a]
**1**	1 : 2	$InCl_3$ (0.1 equiv.), toluene, rt, 15 h	32%	41%
**2**	2 : 1	HCl (8.5 mM), H_2O, <5 °C, 12 h	trace	84%
**3**	1 : 1.5	HCl (8.5 mM), H_2O, <5 °C, 12 h	20%	58%
**4**	1 : 2	HCl (8.5 mM), H_2O, <5 °C, 12 h	30%	60%
**5**	1 : 3	HCl (8.5 mM), H_2O, <5 °C, 12 h	29%	68%
**6**	1 : 2	HCl (8.5 mM), DMSO, <5 °C, 12 h	26%	37%
**7**	1 : 2	HCl (8.5 mM), DMSO/H_2O, <5 °C, 12 h	21%	65%
**8**	1 : 2	TFA (0.5 equiv.), CH_2Cl_2, rt, 16 h	37%	trace
**9**	1 : 2	TFA (0.5 equiv.), CH_2Cl_2, reflux, 4 h	42%	trace
**10**	1 : 2	TFA (2.0 equiv.), CH_2Cl_2, reflux, 4 h	26%	trace

[a] Isolated yield.

Table S2. Optimization for the synthesis of dipyrromethane **22**.

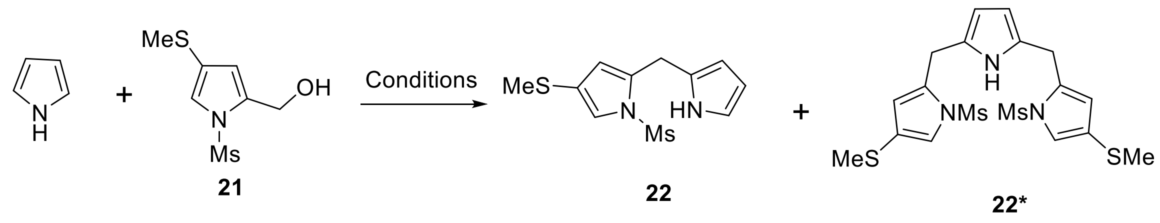

Entry	Pyrrole : 21	Conditions	22 (%)[a]	22* (%)[a]
**1**	4 : 1	HCl (8.5 mM), H_2O, <5 ºC, 12 h	trace	ND[b]
**2**	7 : 1	$InCl_3$ (0.2 equiv.), toluene, rt, 15 h	ND[b]	ND[b]
**3**	7 : 1	TFA (0.5 equiv.), CH_2Cl_2, reflux, 4 h	24%	ND[b]
**4**	7 : 1	BF_3·MeOH (0.26 equiv.), MeOH, $CHCl_3$, 50 ºC, 16 h	70%	ND[b]

[a] Isolated yield. [b] Not detected.

3-(Methylthio)-1-(triisopropylsilyl)-1*H*-pyrrole (3). To a stirred solution of **2** (36.28 g, 120.0 mmol) in anhydrous THF (200 mL), *n*-BuLi (2.5 M in *n*-Hexane, 72.0 mL, 180.0 mmol) was added at -78 ºC under nitrogen atmosphere. The solution was stirred at this temperature for 6 h, then Me_2S_2 (22.60 g, 240.0 mmol) were added, and the mixture was left to reach room temperature (rt) under stirring overnight. The mixture was poured into water (400 mL) and was extracted with DCM (3 × 200 mL). The combined organic phases were washed with water (300 mL) and brine (300 mL), dried over Na_2SO_4 and concentrated to dryness *in vacuo*. The resulting residue was purified by silica gel chromatography (5 × 20, PE:EtOAc 99:1) to give **3** (29.07 g, 107.9 mmol) as a light yellow liquid in 90% yield. R_f = 0.5 (PE:EtOAc 99:1). ^1H NMR (400 MHz, $CDCl_3$): δ (ppm) 6.76 (t, J = 2.0 Hz, 1H), 6.74 (t, J = 2.8 Hz, 1H), 6.32 (q, J = 1.2 Hz, 1H), 2.36 (s, 3H), 1.46-1.38 (m, 3H), 1.08 (d, J = 7.6 Hz, 18H). ^{13}C NMR (100 MHz, $CDCl_3$): δ (ppm) 125.8, 125.1, 116.9, 113.6, 20.7, 17.9, 11.7. IR (neat): ν (cm^{-1}) 2946, 2867, 1650, 1466, 1259, 1083, 692. HRMS (ESI): m/z $[M+H]^+$ Calcd for $C_{14}H_{27}NSSi$: 270.1712; Found: 270.1710. Anal. Calcd for $C_{14}H_{27}NSSi$•1/10 H_2O: C 61.98, H 10.11, N 5.16, S 11.82. Found: C 61.96, H 10.22, N 5.16, S 11.74.

3-(Methylthio)-1*H*-pyrrole (4). A solution of TBAF (1 M in THF, 87.0 mL, 87.0 mmol) was added to a stirred solution of **3** (23.5 g, 87.0 mmol) in THF (60 mL). The mixture was stirred at rt for 20 min. The solvent was removed to dryness *in vacuo*, and the resulting residue was purified by silica gel chromatography (5 × 30, PE:EtOAc 80:20 → PE:EtOAc 0:100) to give **4** (9.85 g, 87.0 mmol) as a colorless liquid in 100% yield. The compound turns brown within minutes due to degradation. R_f = 0.5 (PE:EtOAc 80:20). ^1H NMR (400 MHz, $CDCl_3$): δ (ppm) 8.31 (br, 1H), 6.81 (q, J = 2.0 Hz, 1H), 6.77 (dd, J = 2.8 and 4.8 Hz, 1H), 6.29-6.27 (m, 1H), 2.36 (s, 3H). ^{13}C NMR (100 MHz, $CDCl_3$): δ (ppm) 119.9, 118.9, 115.0, 111.8, 20.7. IR (neat): ν (cm^{-1}) 3387, 2918, 1686, 1523, 1417, 1069, 778, 709. HRMS (ESI): m/z $[M+H]^+$ Calcd for C_5H_7NS: 114.0377; Found: 114.0375.

3-(Methylthio)-1*H*-pyrrole-2-carboxaldehyde (**5**). $POCl_3$ (8.22 mL, 88.2 mmol) was added dropwise to stirred anhydrous DMF (6.94 mL, 89.6 mmol) at 0 ºC under nitrogen atmosphere. The solution was stirred at rt for 15 min and then 1,2-dichloroethane (40 mL) was added to the solution. The mixture was cooled to 0 ºC. A solution of **4** (9.34 g, 82.5 mmol) in 1,2-dichloroethane (40 mL) was added via a cannula, and the reaction was heated at reflux for 2 h before cooling to rt. A solution of sodium acetate (aq., sat., 60 mL) was added, and the reaction was brought to reflux for another 1 h and was left cooling to rt. The cooled reaction mixture was extracted with CH_2Cl_2 (3 × 30 mL). The combined organic phases were washed with brine (50 mL) and dried over Na_2SO_4. The solvent was removed to dryness *in vacuo*, and the resulting residue was purified by silica gel chromatography (5 × 15, PE:EtOAc 80:20) to give **5** (10.02 g, 70.97 mmol) as a white solid in 86% yield. R_f = 0.4 (PE:EtOAc 80:20). Mp: 75.8-76.5 ºC. ^1H NMR (400 MHz, DMSO-d_6): δ (ppm) 12.13 (br, 1H), 9.57 (d, J = 0.8 Hz, 1H), 7.24-7.22 (m, 1H), 6.31 (t, J = 2.4 Hz, 1H), 2.42 (s, 3H). ^{13}C NMR (100 MHz, DMSO-d_6): δ (ppm) 176.7, 129.4, 129.1, 127.2,

110.6, 16.7. IR (neat): ν (cm^{-1}) 3247, 2839, 1629, 1404, 1340, 782. HRMS (ESI): m/z $[M+H]^+$ Calcd for C_6H_7NOS: 142.0327; Found: 142.0326. Anal. Calcd for C_6H_7NOS: C 51.04, H 5.00, N 9.92. Found: C 51.07, H 5.05, N 9.83.

1-(Methylsulfonyl)-3-(methylthio)-1*H*-pyrrole-2-carboxaldehyde (6). To a suspension of sodium hydride (60% dispersion in mineral oil, 2.46 g, 61.6 mmol) in THF (140 mL), a solution of **5** (7.25 g, 51.3 mmol) in THF (60 mL) was added dropwise at 0 ºC. Then the mixture was stirred at rt for 1 h. After cooling to 0 ºC, methanesulfonyl chloride (5.16 mL, 66.7 mmol) was added dropwise, and the reaction was stirred at rt overnight. The reaction was diluted with H_2O (30 mL) and concentrated *in vacuo*. The residual aqueous phase was extracted with DCM (3 × 30 mL), and the combined organic phases were washed successively with sodium bicarbonate (aq., sat., 50 mL), H_2O (50 mL) and brine (50 mL). The combined organic phases were dried over Na_2SO_4 and concentrated to dryness *in vacuo*. The resulting residue was purified by silica gel chromatography (5 × 15, PE:EtOAc 70:30) to give **6** (9.34 g, 42.6 mmol) as a purple solid in 83% yield. R_f = 0.4 (PE:EtOAc 70:30). Mp: 141.8-142.3 ºC. ^{1}H NMR (400 MHz, DMSO-d_6): δ (ppm) 9.96 (d, J = 0.4 Hz, 1H), 7.61 (d, J = 3.2 Hz, 1H), 6.63 (dd, J = 3.2 and 0.8 Hz, 1H), 3.73 (s, 3H), 2.45 (s, 3H). ^{13}C NMR (100 MHz, DMSO-d_6): δ (ppm) 178.2, 138.7, 129.3, 126.5, 110.2, 43.0, 14.9. IR (neat): ν (cm^{-1}) 3029, 2930, 1643, 1438, 1345, 1154, 796. HRMS (ESI): m/z $[M+H]^+$ Calcd for $C_7H_9NO_3S_2$: 220.0102; Found: 220.0101. Anal. Calcd for $C_7H_9NO_3S_2$: C 38.34, H 4.14, N 6.39; found: C 38.35, H 4.13, N 6.38.

1-(Methylsulfonyl)-3-(methylthio)-1*H*-pyrrole-2-methanol (7). To a solution of **6** (7.40 g, 33.8 mmol) in a mixture of methanol (27 ml) and THF (270 ml), $NaBH_4$ (10.21 g, 269.9 mmol) was slowly added in portions at 0 ºC. The reaction was stirred at rt for 2 h. The reaction was quenched with H_2O (3 mL), and the solvents were concentrated *in vacuo*. The residue was washed with water (60 mL) and extracted with CH_2Cl_2 (3 × 50 mL). The combined organic phases were dried over Na_2SO_4 and concentrated to dryness *in vacuo*. The residue was purified by silica gel chromatography (5 × 17, PE:EtOAc 60:40) to give **7** (7.01 g, 31.7 mmol) as a white solid in 94% yield. R_f = 0.5 (PE:EtOAc 60:40). Mp: 92.8-93.4 ºC. ^{1}H NMR (400 MHz, DMSO-d_6): δ (ppm) 7.21 (d, J = 3.6 Hz, 1H), 6.43 (d, J = 3.6 Hz, 1H), 5.31 (t, J = 4.8 Hz, 1H), 4.66 (d, J = 4.8 Hz, 2H), 3.58 (s, 3H), 2.32 (s, 3H). ^{13}C NMR (100 MHz, DMSO-d_6): δ (ppm) 132.8, 122.6, 120.6, 112.9, 52.2, 42.7, 18.5. IR (neat): ν (cm^{-1}) 3401, 3027, 2931, 1363, 1326, 1176, 1155, 1003, 776. HRMS (ESI): m/z $[M+Na]^+$ Calcd for $C_7H_{11}NO_3S_2$: 244.0078; Found: 244.0084. Anal. Calcd for $C_7H_{11}NO_3S_2$: C 37.99, H 5.01, N 6.33. Found: C 37.83, H 5.10, N 6.30.

2,5-Bis((1-(methylsulfonyl)-3-(methylthio)-1*H*-pyrrol-2-yl)methyl)-1*H*-pyrrole (8). To a solution of **7** (4.42 g, 20.0 mmol) and pyrrole (0.671 g, 9.99 mmol) in CH_2Cl_2 (60 mL), TFA (0.571 g, 4.99 mmol) was added. The reaction mixture was stirred at reflux for 4 h. After cooling to rt, the solvents were removed to dryness *in vacuo*, and the resulting residue was purified by silica gel chromatography (3 × 20, PE:EtOAc 80:20) to give **8** (1.99 g, 4.20 mmol) as a white solid in 42% yield. R_f = 0.5 (PE:EtOAc

80:20). Mp: >133.7 ºC (decomposition). ^{1}H NMR (400 MHz, $CDCl_3$): δ (ppm) 8.44 (br, 1H), 7.08 (d, J = 3.6 Hz, 2H), 7.34 (d, J = 3.6 Hz, 2H), 5.84 (d, J = 2.8 Hz, 2H), 4.23 (s, 4H), 2.63 (s, 6H), 2.34 (s, 6H). ^{13}C NMR (100 MHz, $CDCl_3$): δ (ppm) 132.7, 127.4, 121.9, 120.1, 114.6, 107.3, 42.4, 23.6, 19.2. IR (neat): ν (cm^{-1}) 3415, 3012, 2922, 1361, 1176, 1121, 773. HRMS (ESI): m/z [M+H]$^+$ Calcd for $C_{18}H_{23}N_3O_4S_4$: 474.0650; Found: 474.0644. Anal. Calcd for $C_{18}H_{23}N_3O_4S_4$: C 45.65, H 4.89, N 8.87. Found: C 45.90, H 5.01, N 8.71.

2,5-Bis((3-(methylthio)-1*H*-pyrrol-2-yl)methyl)-1*H*-pyrrole (9). To a solution of **8** (0.237 g, 0.500 mmol) in dry methanol (5 mL), KOH (0.628 g, 11.2 mmol) was added. The reaction mixture was stirred at rt overnight, washed with water (5 mL) and extracted with DCM (3 × 5 mL). The combined organic phases were dried over Na_2SO_4 and concentrated to dryness *in vacuo* to give **9** (0.152 g, 0.479 mmol) as a dark yellow solid in 96% yield. R_f = 0.5 (PE:EtOAc 80:20). Mp: >112 ºC (dec). ^{1}H NMR (400 MHz, DMSO-d_6): δ (ppm) 10.74 (s, 2H), 10.2 (s, 1H), 6.63 (t, J = 2.8 Hz, 2H), 6.04 (t, J = 2.4 Hz, 2H), 5.48 (d, J = 2.8 Hz, 2H), 3.85 (s, 4H), 2.13 (s, 6H). ^{13}C NMR (100 MHz, DMSO-d_6): δ (ppm) 132.2, 128.6, 116.7, 111.6, 109.1, 105.0, 23.9, 20.7. IR (neat): ν (cm^{-1}) 3363, 3105, 2917, 1611, 1419, 1312, 1132, 1074, 764, 718. HRMS (ESI): m/z [M+Na]$^+$ Calcd for $C_{16}H_{19}N_3S_2$: 340.0918; Found: 340.0918. Anal. Calcd for $C_{16}H_{19}N_3S_2$: C 60.53, H 6.03, N 13.24. Found: C 60.36, H 6.06, N 13.21.

2,13-Bis(methylthio)porphyrin (P1). To a vigorously stirred solution of TFA (3.70 mL, 48.3 mmol) in chloroform (48 mL), a solution of **9** (0.152 g, 0.479 mmol) in chloroform (5 mL) and a solution of aldehyde **11** (59.0 mg, 0.479 mmol) in chloroform (5 mL) were added simultaneously during 10 min at rt. The reaction mixture was stirred for 20 min, then a solution of DDQ (0.108 g, 0.479 mmol) in toluene (1.0 mL) was added in one portion. After an additional 10 min of stirring, the reaction mixture was quenched by triethanolamine (7.15 g, 47.9 mmol). The resulting mixture was then cooled for 1 h in the refrigerator, filtered using a glass sinter (G3), and the filtrate was collected. The collected black crystals consisting of triethanoloammonium trifluoroacetate were washed with DCM until they became pale green. The combined filtrates were evaporated to dryness *in vacuo*. The resulting residue was purified by silica gel chromatography (3 × 15, DCM) to give **P1** (17.31 mg, 0.04300 mmol) as a dark red solid in 9% yield. R_f = 0.4 (PE:EtOAc 80:20). Mp: >295 ºC (dec). ^{1}H NMR (400 MHz, TFA-d): δ (ppm) 11.05 (s, 2H), 10.79 (s, 2H), 9.80 (s, 2H), 9.77 (s, 2H), 9.45 (s, 2H), 3.38 (s, 6H). ^{13}C NMR (100 MHz, TFA-d): δ (ppm) 153.4, 147.5, 147.2, 144.3, 142.4, 134.5, 133.0, 125.6, 105.1, 104.9, 17.1. IR (neat): ν (cm^{-1}) 3313, 3097, 2920, 1424, 1227, 1061, 974, 840, 775, 744. HRMS (ESI): m/z [M+H]$^+$ Calcd for $C_{22}H_{18}N_4S_2$: 403.1051; Found: 403.1048. Anal. Calcd for $C_{22}H_{18}N_4S_2 \cdot 3/10\ H_2O$: C 64.78, H 4.60, N 13.73. Found: C 64.77, H 4.53, N 13.48. λ_{abs} (toluene) 422, 556, 584, 600 nm.

[2,13-Bis(methylthio)porphinato]zinc(II) (Zn-P1). To a solution of **P1** (47.90 mg, 0.1190 mmol) in DCM (30 mL), a solution of zinc acetate dehydrate (0.17 g, 0.78 mmol) in methanol (7.5 mL) was added. The reaction mixture was stirred in the dark overnight at rt. The solvents were removed *in vacuo*, and

the resulting residue was purified by silica gel chromatography (3 × 17, DCM:THF 98:2) to give **Zn-P1** (49.85 mg, 0.1070 mmol) as a dark red solid in 90% yield. R_f = 0.8 (DCM). Mp: >370 ºC. ^{1}H NMR (400 MHz, THF-d_8): δ (ppm) 10.28 (s, 2H), 10.09 (s, 2H), 9.49 (s, 2H), 9.45 (s, 2H), 9.17 (s, 2H), 3.22 (s, 6H). ^{13}C NMR (100 MHz, THF-d_8): δ (ppm) 150.5, 149.89, 149.88, 147.7, 143.3, 132.4, 132.2, 126.5, 103.6, 101.3, 17.2. IR (neat): ν (cm^{-1}) 2952, 2921, 2853, 2183, 2160, 1455, 1262, 1126, 1004, 856. HRMS (ESI): m/z $[M]^{\bullet+}$ Calcd for $C_{22}H_{16}N_4S_2Zn$: 464.0108; Found: 464.0101. Anal. Calcd for $C_{22}H_{16}N_4S_2Zn$•1/5 THF: C 57.01, H 3.69, N 11.66. Found: C 57.17, H 3.50, N 11.68. λ_{abs} (toluene) 417, 543, 579 nm.

2-((1*H*-pyrrol-2-yl)methyl)-1-(methylsulfonyl)-3-(methylthio)-1*H*-pyrrole (12). To a solution of **7** (0.89 g, 4.0 mmol) and pyrrole (0.55 mL, 8.0 mmol) in H_2O (10 mL), HCl (8.5 mM, 10 mL) was added at <5 ºC under nitrogen atmosphere. The reaction mixture was stirred at <5 ºC for 12 h and subsequently extracted with CH_2Cl_2 (3 × 20 mL). The combined organic phases were dried over Na_2SO_4 and concentrated *in vacuo*. The residue was purified by silica gel chromatography (3 × 15, PE:EtOAc 80:20) to afford **12** (0.910 g, 3.37 mmol) as a white solid in 84% yield. R_f = 0.5 (PE:EtOAc 80:20). Mp: 80.7-81.6 ºC. ^{1}H NMR (400 MHz, DMSO-d_6): δ (ppm) 1.57 (br, 1H), 7.17 (d, J = 3.2 Hz, 1H), 6.60 (td, J = 2.4 and 1.6 Hz, 1H), 6.49 (d, J = 3.2 Hz, 1H), 5.88 (dd, J = 5.6 and 2.8 Hz, 1H), 5.51-5.48 (m, 1H), 4.19 (s, 2H), 2.82 (s, 3H), 2.32 (s, 3H). ^{13}C NMR (100 MHz, DMSO-d_6): δ (ppm) 131.8, 127.7, 121.8, 119.2, 116.7, 113.7, 107.4, 105.5, 41.6, 23.3, 18.6. IR (neat): ν (cm^{-1}) 3426, 3143, 3009, 2921, 1565, 1356, 1268, 1205, 1172, 1121, 760, 717, 577. HRMS (ESI): m/z $[M+H]^+$ Calcd for $C_{11}H_{14}N_2O_2S_2$: 293.0394. Found: 293.0397.

2-((1*H*-pyrrol-2-yl)methyl)-3-(methylthio)-1*H*-pyrrole (13). To a solution of **12** (0.54 g, 2.0 mmol) in dry methanol (12 mL), KOH (1.25 g, 22.4 mmol) was added. The reaction mixture was stirred at rt overnight, washed with water (10 mL) and extracted with DCM (3 × 10 mL). The combined organic phases were dried over Na_2SO_4 to give **13** (0.349 g, 1.82 mmol) as a yellow liquid in 91% yield. R_f = 0.5 (PE:EtOAc 80:20). ^{1}H NMR (400 MHz, DMSO-d_6): δ (ppm) 10.74 (s, 1H), 10.46 (s, 1H), 6.63 (t, J = 2.8 Hz, 1H), 6.57 (dd, J = 4.0 and 2.8 Hz, 1H), 6.03 (t, J = 2.8 Hz, 1H), 5.86 (q, J = 2.8 Hz, 1H), 5.60 (s, 1H), 3.89 (s, 2H), 2.12 (S, 3H). ^{13}C NMR (100 MHz, DMSO-d_6): δ (ppm) 132.0, 129.6, 116.7, 116.3, 111.6, 109.2, 107.2, 105.0, 23.8, 20.7. IR (neat): ν (cm^{-1}) 3372, 3104, 2917, 1565, 1421, 1026, 969, 718. HRMS (ESI): m/z $[M+Na]^+$ Calcd for $C_{10}H_{12}NO_2S$: 215.0619. Found: 215.0615.

5-((3-(Methylthio)-1*H*-pyrrol-2-yl)methyl)-1*H*-pyrrole-2-carbaldehyde (14). $POCl_3$ (0.11 mL, 1.18 mmol) was added dropwise to stirred anhydrous DMF (0.72 mL) at 0 °C under nitrogen atmosphere. The mixture was stirred at 0 ºC for 15 min. To a solution of **12** (0.200 g, 1.04 mmol) in DMF (5 mL), the freshly prepared Vilsmeier reagent (0.72 mL) was added at 0 ºC and stirred under nitrogen atmosphere. The resulting solution was stirred at 0 °C for 2 h. The reaction mixture was diluted with 1,2-dichloroethane (6 mL), and sodium acetate (aq., sat., 6 mL) was added. The resulting mixture was

stirred at reflux for 1 h. After cooling to rt, the reaction mixture was extracted with DCM (2 × 10 mL). The combined organic phases were successively washed with sodium carbonate (aq., sat., 10 mL), H_2O (10 mL) and brine (10 mL), dried over Na_2SO_4 and concentrated to dryness *in vacuo*. The resulting residue was purified by silica gel chromatography (3 × 12, PE:EtOAc 60:40) to give **14** (0.146 g, 0.666 mmol) as a white solid in 64% yield. R_f = 0.5 (PE:EtOAc 60:40). Mp: 95.3-96.1 ºC. ^{1}H NMR (400 MHz, DMSO-d_6): δ (ppm) 11.92 (br, 1H), 10.82 (br, 1H), 9.35 (s, 1H), 6.87 (dd, J = 3.6 and 2.4 Hz, 1H), 6.69 (t, J = 2.8 Hz, 1H), 6.07 (t, J = 2.8 Hz, 1H), 5.84 (dd, J = 3.6 and 2.4 Hz, 1H), 3.98 (s, 2H), 2.13 (s, 3H). ^{13}C NMR (100 MHz, DMSO-d_6): δ (ppm) 178.2, 140.4, 132.0, 130.1, 121.7, 117.3, 111.8, 110.1, 109.1, 23.9, 20.6. IR (neat): ν (cm^{-1}) 3270, 3122, 2917, 2829, 1630, 1494, 1185, 1043, 779, 721. HRMS (ESI): m/z [M+H]$^+$ Calcd for $C_{11}H_{12}N_2OS$: 221.0749. Found: 221.0744. Anal Calcd for $C_{11}H_{12}N_2OS$: C 59.98, H 5.49, N 12.72. Found: C 59.92, H 5.47, N 12.71.

2,7-Di(methylthio)-*N*^{5},*N*^{5},*N*^{10},*N*^{10}-tetramethyl-5*H*,10*H*-dipyrrolo[1,2-*a*:1',2'-*d*]pyrazine-5,10-diamine (18). To a stirred solution of **17** (3.92 g, 9.80 mmol) in anhydrous THF (200 mL), *t*-BuLi (1.6 M in pentane, 25.0 mL, 40.0 mmol) was added at -78 ºC under nitrogen atmosphere. The mixture was stirred at -78 ºC for 1 h, and then Me_2S_2 (3.76 g, 40.0 mmol) was added. The reaction mixture was stirred at -78 ºC for additional 5 h and was left to reach rt overnight under stirring. The mixture was quenched by ammonium chloride (aq., sat., 40 mL), and the organic solvents were removed *in vacuo*. The resulting aqueous phase was extracted with DCM (3 × 50 mL). The combined organic phases were dried over Na_2SO_4, and the solvents were removed to dryness *in vacuo*. The resulting dark residue was washed with EtOAc to yield **18** (2.40 g, 7.15 mmol) as an off-white solid in 73% yield. Mp: >100 °C (decomposition). ^{1}H NMR (400 MHz, $CDCl_3$): δ (ppm) 6.93 (d, J = 1.6 Hz, 2H), 6.20 (dd, J = 1.6 and 0.8 Hz, 2H), 5.75 (d, J = 0.8 Hz, 2H), 2.37 (s, 6H), 2.24 (s, 12H). ^{13}C NMR (100 MHz, $CDCl_3$): δ (ppm) 126.3, 121.0, 115.8, 108.9, 71.9, 39.3, 20.4. IR: ν (cm^{-1}) 2935, 2915, 2860, 2826, 1475, 1358, 1304, 1187, 1090, 1002, 797, 628. HRMS (ESI): m/z [M+Na]$^+$ Calcd for $C_{16}H_{24}N_4S_2$: 359.1340; Found: 359.1388. Anal. Calcd $C_{16}H_{24}N_4S_2$•3/10 H_2O: C 56.21, H 7.07, N 16.34. Found: C 56.22, H 7.07, N 16.34.

4-(Methylthio)-1*H*-pyrrole-2-carboxaldehyde (19). To a solution of compound **18 (**4.30 g, 12.8 mmol**)** in THF (50 mL), $NaHCO_3$ (aq., sat., 50 mL) was added, and the resulting reaction mixture was stirred at reflux for 15 h. After cooling to rt, the reaction mixture was extracted with DCM (3 × 50 mL). The combined organic phases were dried over Na_2SO_4 and evaporated to dryness *in vacuo*. The resulting residue was purified by silica gel chromatography (5 × 15, PE:EtOAc 80:20) to give **19** (3.60 g, 25.5 mmol) as a light yellow solid in 99% yield. R_f = 0.4 (PE:EtOAc 80:20). Mp: 82.5-83.7 °C. ^{1}H NMR (400 MHz, DMSO-d_6): δ (ppm) 12.26 (s, 1H), 9.43 (d, J = 0.8 Hz, 1H), 7.25 (dd, J = 1.6 and 0.8 Hz, 1H), 7.02 (d, J = 1.6 Hz, 1H), 2.33 (s, 3 H). ^{13}C NMR (100 MHz, DMSO-d_6): δ (ppm) 179.1, 133.2, 127.7, 121.6, 117.5, 19.0. IR (neat): ν (cm^{-1}) 3161, 3106, 2982, 2747, 2681, 1679, 1372, 1351, 1235,

812, 758. HRMS (ESI): m/z $[M+H]^+$ Calcd for C_6H_7NOS: 142.0327; Found: 142.0325. Anal. Calcd for C_6H_7NOS: C 51.04, H 5.00, N 9.92. Found: C 51.25, H 5.02, N 9.83.

1-(Methylsulfonyl)-4-(methylthio)-1*H*-pyrrole-2-carboxaldehyde (20). To a suspension of sodium hydride (60% dispersion in mineral oil, 1.29 g, 32.2 mmol) in THF (60 mL), a solution of **19** (3.50 g, 24.8 mmol) in THF (60 mL) was added dropwise at 0 °C under stirring. The reaction mixture was stirred at rt for 1 h. After cooling to 0 °C, methanesulfonyl chloride (5.16 mL, 34.7 mmol) was added dropwise, and the reaction mixture was stirred at rt overnight. The reaction mixture was diluted with H_2O (50 mL), and organic solvents were removed *in vacuo*. The residual aqueous phase was extracted with DCM (3 × 50 mL), and the combined organic phases were washed successively with sodium carbonate (aq., sat., 50 mL), H_2O (50 mL) and brine (50 mL). The combined organic phases were dried over Na_2SO_4 and concentrated to dryness *in vacuo*. The resulting residue was purified by silica gel chromatography (5 × 17, PE:EtOAc 70:30) to give **20** (4.11 g, 18.7 mmol) as a light pink solid in 76% yield. R_f = 0.5 (PE:EtOAc 70:30). Mp: 63.1-64.0 °C. ^{1}H NMR (400 MHz, DMSO-d_6): δ (ppm) 9.74 (d, J = 0.4 Hz, 1H), 7.52 (dd, J = 2.0 and 0.4 Hz, 1H), 7.40 (d, J = 2.0 Hz, 1H), 3.79 (s, 3H), 2.44 (s, 3H). ^{13}C NMR (100 MHz, DMSO-d_6): δ (ppm) 179.2, 133.2, 126.9, 126.1, 120.6, 42.6, 16.8. IR (neat): ν (cm^{-1}) 2925, 2867, 1678, 1439, 1366, 1169, 1060, 799, 747. HRMS (ESI): m/z $[M+H]^+$ Calcd for $C_7H_9NO_3S_2$: 220.0102; Found: 220.0100. Anal. Calcd for $C_7H_9NO_3S_2$: C 38.34, H 4.14, N 6.39, S 29.24. Found: C 38.42, H 4.11, N 6.37.

1-(Methylsulfonyl)-4-(methylthio)-1*H*-pyrrole-2-methanol (21). To a solution of **20** (2.92 g, 13.3 mmol) in a mixture of methanol (11 mL) and THF (110 mL), $NaBH_4$ (4.030 g, 106.5 mmol) was slowly added in four portions at 0 °C. The reaction mixture was stirred at rt for 1h. The reaction was quenched with H_2O (3 mL), and the solvents were removed *in vacuo*. The residue was washed with distilled H_2O (30 mL) and extracted with DCM (3 × 30 mL). The combined organic phases were dried over Na_2SO_4 and concentrated to dryness *in vacuo*. The residue was purified by silica gel chromatography (3 × 15, PE:EtOAc 50:50) to give **21** (2.89 g, 13.1 mmol) as a colorless liquid in 98% yield. R_f = 0.5 (PE:EtOAc 60:40). ^{1}H NMR (400 MHz, DMSO-d_6): δ (ppm) 7.00 (d, J = 2.0 Hz, 1H), 6.32 (d, J = 2.0 Hz, 1H), 5.34 (t, J = 4.8 Hz, 1H), 4.54 (d, J = 4.8 Hz, 2H), 3.52 (s, 3H), 2.35 (s, 3H). ^{13}C NMR (100 MHz, DMSO-d_6): δ (ppm) 135.7, 119.6, 118.6, 114.6, 55.2, 42.4, 16.8. IR (neat): ν (cm^{-1}) 3393, 2925, 1357, 1172, 1148, 1064, 932, 779. HRMS (ESI): m/z $[M+Na]^+$ Calcd for $C_7H_{11}NO_3S_2$: 244.0078; Found: 244.0071. Anal. Calcd for $C_7H_{11}NO_3S_2$•1/5 H_2O: C 37.39, H 5.11, N 6.23; found: C 37.35, H 4.94, N 6.24.

2-((1*H*-pyrrol-2-yl)methyl)-1-(methylsulfonyl)-4-(methylthio)-1*H*-pyrrole (22). To a solution of **21** (2.71 g, 12.2 mmol) and pyrrole (5.77 g, 86.0 mmol) in a mixture of MeOH (12 mL) and $CHCl_3$ (144 mL), $BF_3 \cdot CH_3OH$ (10% in methanol) (2.45 mL, 3.19 mmol) was added and stirred at rt under nitrogen atmosphere for 1 h, and stirred at 50 °C for 16 h. The reaction mixture was diluted with H_2O (100 mL) and extracted with DCM (2 × 50 mL). The combined organic phases were dried over Na_2SO_4 and

concentrated to dryness *in vacuo*. The resulting residue was purified by silica gel chromatography (3 × 15, PE:EtOAc 80:20) to give **22** (2.32 g, 8.58 mmol) as a light yellow liquid in 70% yield. R_f = 0.5 (PE:EtOAc 80:20). ^{1}H NMR (400 MHz, DMSO-d_6): δ (ppm) 10.64 (s, 1H), 6.96 (d, J = 2.0 Hz, 1H), 6.65 (dd, J = 4.0 and 1.6 Hz, 1H), 5.95-5.92 (m, 2H), 5.81-5.80 (m, 1H), 4.05 (s, 2H), 3.12 (s, 3H), 2.33 (s, 3H). ^{13}C NMR (100 MHz, DMSO-d_6): δ (ppm) 134.6, 127.1, 119.1, 118.6, 117.0, 113.9, 107.3, 106.4, 41.9, 25.4, 16.6. IR (neat): ν (cm^{-1}) 3418, 3011, 2922, 1357, 1173, 1130, 1062, 963, 778. HRMS (ESI): m/z [M+H]$^+$ Calcd for $C_{11}H_{14}N_2O_2S_2$: 271.0575; Found: 271.0570. Anal. Calcd for $C_{11}H_{14}N_2O_2S_2$: C 48.87, H 5.22, N 10.36. Found: C 48.72, H 5.19, N 10.34.

5-((1-(Methylsulfonyl)-4-(methylthio)-1*H*-pyrrol-2-yl)methyl)-1*H*-pyrrole-2-carbaldehyde (23). $POCl_3$ (0.90 mL, 9.7 mmol) was added dropwise to stirred anhydrous DMF (6 mL) at 0 °C under nitrogen atmosphere. The mixture was stirred at 0 ºC for 15 min. To a solution of **22** (2.30 g, 8.52 mmol) in DMF (25 mL), the freshly prepared Vilsmeier reagent (5.35 mL) was added at 0 ºC and stirred under nitrogen atmosphere. The resulting solution was stirred at 0 °C for 2 h. The reaction mixture was diluted with 1,2-dichloroethane (40 mL), and sodium acetate (aq., sat., 40 mL) was added. The resulting mixture was stirred at reflux for 1 h. After cooling to rt, the reaction mixture was extracted with DCM (2 × 30 mL). The combined organic phases were successively washed with sodium carbonate (aq., sat., 40 mL), H_2O (40 mL) and brine (40 mL), dried over Na_2SO_4 and concentrated to dryness *in vacuo*. The resulting residue was purified by silica gel chromatography (3 × 12, PE:EtOAc 60:40) to give **23** (2.11 g, 7.07 mmol) as a light yellow solid in 83% yield. R_f = 0.5 (PE:EtOAc 70:30). Mp: >157 °C (dec). ^{1}H NMR (400 MHz, $CDCl_3$): δ (ppm) 9.64 (br, 1H), 9.41 (s, 1H), 6.95 (d, J = 2.0 Hz, 1H), 6.90 (dd, J = 4.0 and 2.8 Hz, 1H), 6.20-6.18 (m, 1H), 6.16-6.15 (m, 1H), 4.21 (s, 2H), 2.76 (s, 3H), 2.37 (s, 3H). ^{13}C NMR (100 MHz, $CDCl_3$): δ (ppm) 178.8, 137.5, 132.5, 131.7, 122.2, 121.4, 119.9, 116.1, 111.1, 42.5, 26.3, 17.7. IR (neat): ν (cm^{-1}) 3247, 2923, 1640, 1358, 1173, 1132, 1062, 779. HRMS (ESI): m/z [M+H]$^+$ Calcd for $C_{12}H_{14}N_2O_3S_2$: 299.0524; Found: 299.0520. Anal. Calcd for $C_{12}H_{14}N_2O_3S_2$: C 48.31, H 4.73, N 9.39, S 21.49. Found: C 48.39, H 4.81, N 9.31.

5-((4-(Methylthio)-1*H*-pyrrol-2-yl)methyl)-1*H*-pyrrole-2-carbaldehyde (24). To a stirred solution of **23** (2.03 g, 6.80 mmol) in methanol (30 mL), sodium hydroxide (aq., 5 M, 8 mL) was added. The reaction mixture was stirred at reflux for 3 h. Ammonium chloride (aq., sat., 20 mL) was added, and the mixture was extracted with DCM (3 × 30 mL). The combined organic phases were washed with water (20 mL) and brine (20 mL), dried over Na_2SO_4 and concentrated to dryness *in vacuo*. The resulting residue was purified by silica gel chromatography (3 × 15, EtOAc:PE 80:20) to give **24** (1.33 g, 6.05 mmol) as a yellow solid in 89% yield. R_f = 0.5 (PE:EtOAc 60:40). Mp: >110 ºC (dec). ^{1}H NMR (400 MHz, $CDCl_3$): δ (ppm) 10.55 (s, 1H), 9.35 (s, 1H), 8.89 (s, 1H), 6.97 (dd, J = 3.6 and 2.4 Hz, 1H), 6.71 (dd, J = 2.4 and 1.6 Hz, 1H), 6.18 (dd, J = 3.6 and 2.4 Hz, 1H), 6.09 (dd, J = 2.4 and 1.6 Hz, 1H), 4.00 (s, 2H), 2.33 (s, 3H). ^{13}C NMR (100 MHz, $CDCl_3$): δ (ppm) 179.0, 141.9, 132.2, 128.7, 125.0, 119.8,

115.2, 111.0, 110.2, 26.8, 20.6. IR (neat): ν (cm^{-1}) 3261, 2918, 2827, 1630, 1493, 1412, 1041, 776. HRMS (ESI): m/z [M+H]$^+$ Calcd for $C_{11}H_{12}N_2OS$: 221.0749; Found: 221.0744. Anal. Calcd for $C_{11}H_{12}N_2OS$: C 59.98, H 5.49, N 12.72. Found: C 60.01, H 5.51, N 12.70.

[2,12-Bis(methylthio)porphinato]magnesium(II) (Mg-P2). To a suspension of **24** (0.11 g, 0.50 mmol) in toluene (5 mL), DBU (0.75 mL, 5.0 mmol) was added dropwise under stirring at rt. To the resulting solution, dried $MgBr_2$ (0.28 g, 1.5 mmol) was added in one portion under stirring. The reaction flask was equipped with a reflux condenser and was heated at 135 °C under air for 19 h. After cooling to rt, the reaction mixture was concentrated to dryness *in vacuo*. The resulting solid was triturated with THF (15.0 mL), sonicated for 1 min, stirred vigorously at rt for 20 min, and then heated to reflux for 1 h. After cooling to rt, the mixture was filtered through a glass sinter (P3) to remove the insoluble black solid, and the resulting filter cake was extracted with hot THF thoroughly until THF phases became colorless. The filtrate was concentrated to dryness *in vacuo*, and the resulting residue was reprecipitated by a THF/petroleum ether system to give **Mg-P2** (74.2 mg, 0.175 mmol) as a purple solid in 70% yield. Mp: >370 ºC. ^{1}H NMR (400 MHz, DMSO-d_6): δ (ppm) 10.20 (s, 2H), 10.18 (s, 2H), 9.57 (d, J = 3.6 Hz, 2H), 9.51 (d, J = 3.6 Hz, 2H), 9.24 (s, 2H), 3.24 (s, 6H). ^{13}C NMR (100 MHz, DMSO-d_6): δ (ppm) 149.1, 148.7, 148.3, 146.3, 142.0, 132.22, 132.18, 126.1, 104.6, 100.9, 16.8. IR (neat): ν (cm^{-1}) 3354, 3020, 2917, 2613, 1619, 1430, 1264, 1074, 1008, 991, 853, 774. HRMS (ESI): m/z [M]$^{\bullet+}$ Calcd for $C_{22}H_{16}MgN_4S_2$: 424.0667; Found: 424.0659. Anal. Calcd for $C_{22}H_{16}MgN_4S_2$: C 62.20, H 3.80, N 13.19. Found: C 62.28, H 3.96, N 13.12. λ_{abs} (toluene) 420, 552, 581, 599 nm.

2,12-Bis(methylthio)porphyrin (P2). To a solution of **Mg-P2** (65 mg, 0.15 mmol) in CH_2Cl_2 (80 mL), TFA (1.5 mL) was added dropwise at rt. The reaction mixture was stirred at rt for 40 min. The reaction mixture was quenched by Et_3N (2.8 mL) and concentrated to dryness *in vacuo*. The resulting residue was purified by chromatography (DCM) to give **P2** (30 mg, 0.075 mmol) as a dark red solid in 50% yield. R_f = 0.8 (PE:DCM 20:80). Mp: >328 ºC (dec). ^{1}H NMR (400 MHz, TFA-d): δ (ppm) 11.04 (s, 2H), 10.94 (s, 2H), 9.85 (d, J = 4.4 Hz, 2H), 8.32 (d, J = 4.4 Hz, 2H), 9.53 (s, 2H), 3.39 (s, 6H). ^{13}C NMR (100 MHz, TFA-d): δ (ppm) 152.0, 145.9, 145.8, 145.5, 143.6, 133.8, 133.6, 126.3, 106.7, 103.2, 17.0. IR (neat): ν (cm^{-1}) 3312, 2922, 2853, 1399, 1224, 1059, 957, 852, 774, 732. HRMS (ESI): m/z [M+H]$^+$ Calcd for $C_{22}H_{18}N_4S_2$: 403.1051; Found: 403.1046. Anal. Calcd for $C_{22}H_{18}N_4S_2$•1/7 H_2O: C 65.23, H 4.55, N 13.83. Found: C 65.20, H 4.49, N 13.88. λ_{abs} (toluene) 379, 407, 503, 543, 581 nm.

[2,12-Bis(methylthio)porphinato]zinc(II) (Zn-P2). To a solution of **P2** (30.0 mg, 0.0745 mmol) in THF (30 mL), zinc acetate dehydrate (0.164 g, 0.745 mmol) was added. The reaction mixture was heated to reflux under stirring in the dark for 2 h. After cooling to room temperature, the solvents were removed *in vacuo*, and the resulting residue was triturated with H_2O (15 mL). The obtained suspension was filtered through a glass sinter (P4), and the solid was washed with H_2O and a small amount of MeOH. The residue was rinsed with THF until the THF phase became colorless. The filtrate was concentrated

to dryness *in vacuo* to give **Zn-P2** (12 mg, 35%) as a purple solid. Mp: >370 ºC. ^{1}H NMR (400 MHz, THF-d_8): δ (ppm) 10.19 (s, 2H), 10.03 (s, 2H), 9.39 (d, J = 4.2 Hz, 2H), 9.36 (d, J = 4.2 Hz, 2H), 9.08 (s, 2H), 3.21 (s, 6H). ^{13}C NMR (126 MHz, THF-d_8): δ (ppm) 149.96, 149.57, 149.15, 147.42, 143.11, 132.36, 132.33, 126.67, 104.71, 101.61, 17.60. HRMS (ESI): m/z [M+H]$^+$ Calcd for $C_{22}H_{16}N_4S_2Zn$: 465.2050; Found: 465.0186. Anal. Calcd for $C_{22}H_{16}N_4S_2Zn$: C 56.72, H 3.46, N 12.03. Found: C 56.51, H 3.50, N 11.91. λ_{abs} (toluene) 418, 549, 579, 594 nm.

2-((1*H*-Pyrrol-2-yl)methyl)-4-(methylthio)-1*H*-pyrrole (25). To a stirred solution of **22** (0.404 g, 0.149 mmol) in methanol (5 mL), KOH (1.87 g, 33.4 mmol) was added. The reaction mixture was stirred at rt overnight, washed with water (10 mL) and extracted with DCM (3 × 5 mL). The combined organic phases were dried over Na_2SO_4 and concentrated to dryness *in vacuo* to give **25** (0.285 g, 1.48 mmol) as a light yellow liquid in 99% yield. R_f = 0.4 (PE:EtOAc 80:20). ^{1}H NMR (400 MHz, DMSO-d_6): δ (ppm) 10.6 (br, 1H), 10.5 (br, 1H), 6.64 (dd, J = 2.4 and 1.6 Hz, 1H), 6.60-6.58 (m, 1H), 5.90 (q, J = 2.8 Hz, 1H), 5.79 (dd, J = 2.4 and 1.6 Hz, 1H), 5.77-5.75 (m, 1H), 3.77 (s, 2H), 2.22 (s, 3H). ^{13}C NMR (100 MHz, DMSO-d_6): δ (ppm) 131.4, 129.3, 118.4, 116.4, 112.7, 108.4, 107.1, 105.3, 25.9, 20.0. IR (neat): ν (cm^{-1}) 3366, 3123, 2917, 1693, 1563, 1418, 1114, 1090, 793, 751, 549. HRMS (ESI): m/z [M+H]$^+$ Calcd for $C_{10}H_{12}N_2S$: 193.0799; Found: 193.0804.

3-(Methylthio)-5-((1*H*-pyrrol-2-yl)methyl)-1*H*-pyrrole-2-carbaldehyde (26). $POCl_3$ (0.11 mL, 1.18 mmol) was added dropwise to stirred anhydrous DMF (0.72 mL) at 0 °C under nitrogen atmosphere. The mixture was stirred at 0 ºC for 15 min. To a solution of **25** (0.10 g, 0.52 mmol) in DMF (4 mL), the freshly prepared Vilsmeier reagent (0.36 mL) was added at 0 ºC and stirred under nitrogen atmosphere. The resulting solution was stirred at 0 °C for 2 h. The reaction mixture was diluted with 1,2-dichloroethane (3 mL), and sodium acetate (aq., sat., 3 mL) was added. The resulting mixture was stirred at reflux for 1 h. After cooling to rt, the reaction mixture was extracted with DCM (2 × 5 mL). The combined organic phases were successively washed with sodium carbonate (aq., sat., 5 mL), H_2O (5 mL) and brine (5 mL), dried over Na_2SO_4 and concentrated to dryness *in vacuo*. The resulting residue was purified by silica gel chromatography (3 × 12, PE:EtOAc 65:35) to give **26** (24.0 mg, 0.109 mmol) as a light green solid in 21% yield. R_f = 0.5 (PE:EtOAc 60:40). Mp: >125 ºC (dec). ^{1}H NMR (400 MHz, DMSO-d_6): δ (ppm) 11.95 (br, 1H), 10.60 (br, 1H), 9.47 (s, 1H), 6.62 (td, J = 2.4 and 1.6 Hz, 1H), 6.05 (d, J = 2.4 Hz, 1H), 5.90 (q, J = 2.4 Hz, 1H), 5.81-5.78 (m, 1H), 3.86 (s, 2H), 2.38 (s, 3H). ^{13}C NMR (100 MHz, DMSO-d_6): δ (ppm) 175.5, 140.8, 130.3, 128.1, 127.5, 116.9, 109.1, 107.3, 105.8, 25.8, 16.6. IR (neat): ν (cm^{-1}) 3232, 2922, 2852, 1611, 1415, 1314, 798, 718. HRMS (ESI): m/z [M+H]$^+$ Calcd for $C_{11}H_{12}N_2OS$: 221.0749; Found: 221.0746. Anal. Calcd for $C_{11}H_{12}N_2OS$: C 59.98, H 5.49, N 12.72. Found: C 59.77, H 5.51, N 12.61.

5-((5-Formyl-1*H*-pyrrol-2-yl)methyl)-3-(methylthio)-1*H*-pyrrole-2-carbaldehyde (27). After purification by silica gel chromatography (3 × 12, PE:EtOAc 50:50) compound **27** (42.6 mg, 0.172

mmol) was obtained as a light yellow solid in 33% yield. R_f = 0.4 (PE:EtOAc 50:50). Mp: >173 ºC (dec). ^{1}H NMR (400 MHz, DMSO-d_6): δ (ppm) 11.02 (br, 1H), 12.01 (br, 1H), 9.48 (s, 1H), 9.37 (s, 1H), 6.91 (dd, J = 3.6 and 2.0 Hz, 1H), 6.12 (s, 1H), 6.08 (dd, J = 3.6 and 2.0 Hz, 1H), 3.94 (s, 2H), 2.39 (s, 3H). ^{13}C NMR (100 MHz, DMSO-d_6): δ (ppm) 178.5, 175.7, 138.7, 138.2, 132.4, 130.3, 128.5, 121.7, 109.7, 109.5, 25.7, 16.7. IR (neat): ν (cm^{-1}) 3223, 2961, 2920, 2835, 1631, 1613, 1476, 1416, 1279, 814, 779. HRMS (ESI): m/z [M+H]$^+$ Calcd for $C_{11}H_{12}N_2O_2S$: 249.0698; Found: 249.0691. Anal. Calcd for $C_{12}H_{12}N_2O_2S$•1/5 H_2O: C 57.22, H 4.96, N 11.12. Found: C 57.24, H 4.70, N 10.94.

Part 3. ^{1}H and ^{13}C NMR Spectra of New Compounds

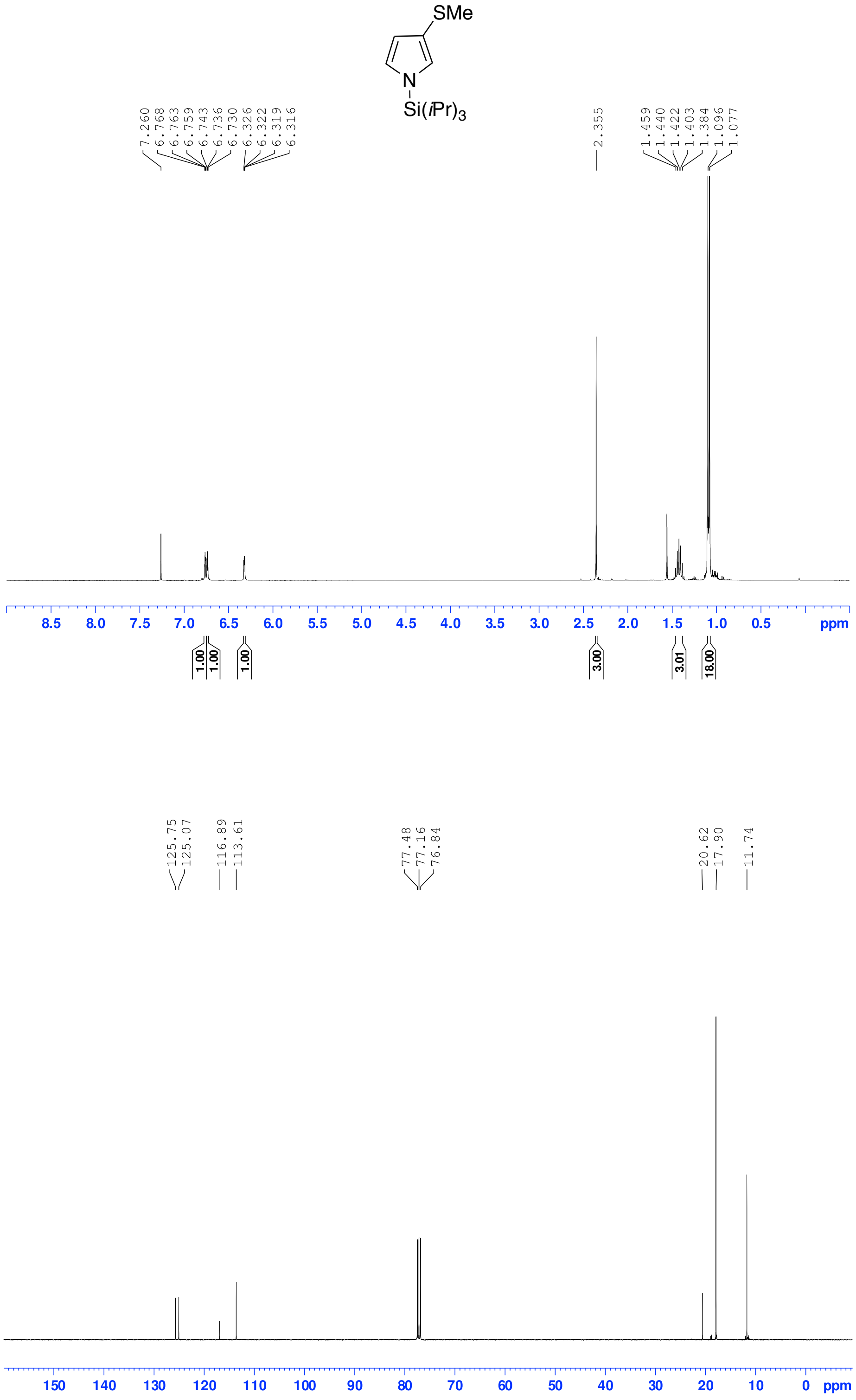

Figure S1. ^{1}H (top) and ^{13}C (bottom) NMR spectra of compound **3** in $CDCl_3$.

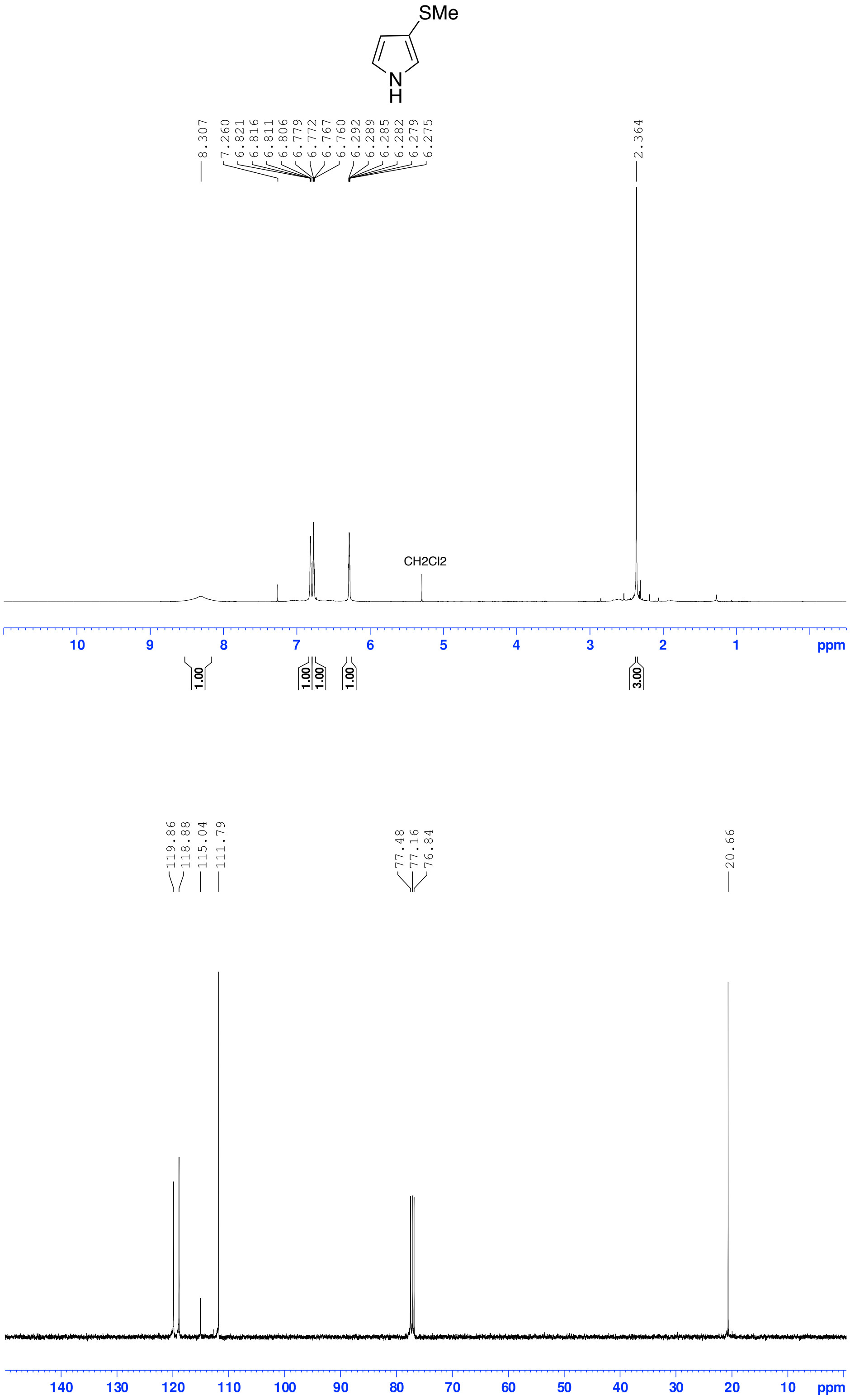

Figure S2. ^{1}H (top) and ^{13}C (bottom) NMR spectra of compound **4** in $CDCl_3$.

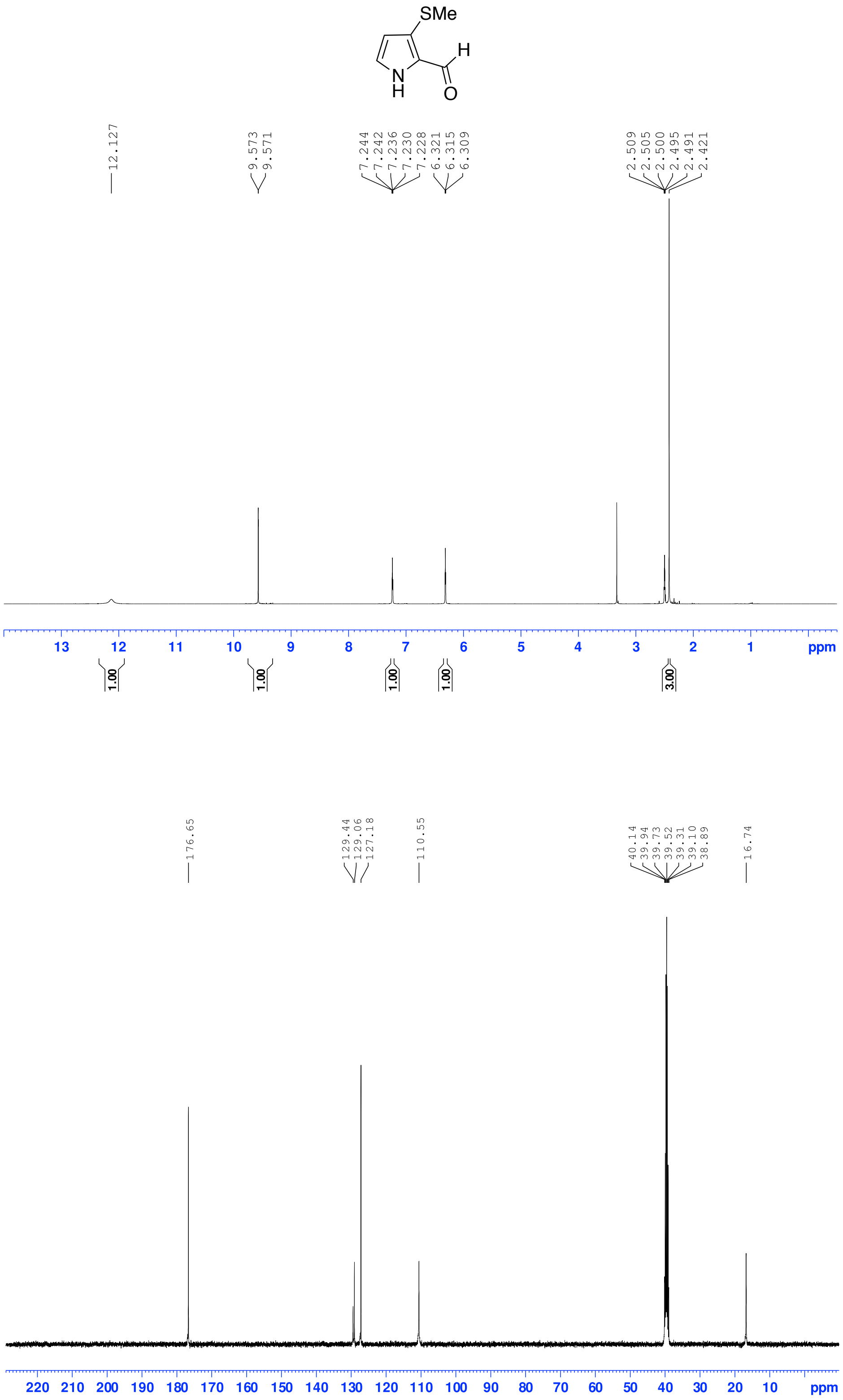

Figure S3. ^{1}H (top) and ^{13}C (bottom) NMR spectra of compound **5** in DMSO-d_6.

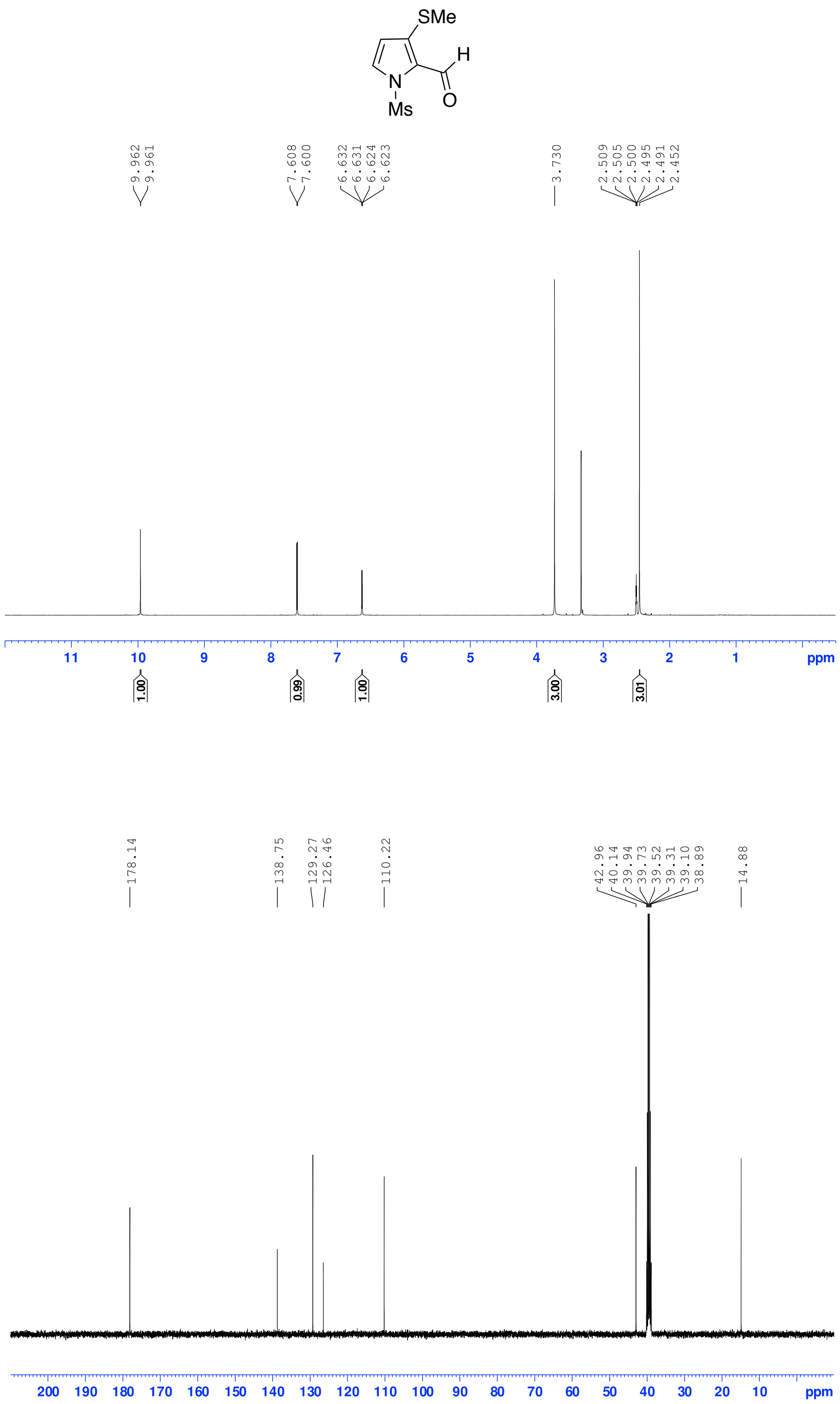

Figure S4. ^{1}H (top) and ^{13}C (bottom) NMR spectra of compound **6** in DMSO-d_6.

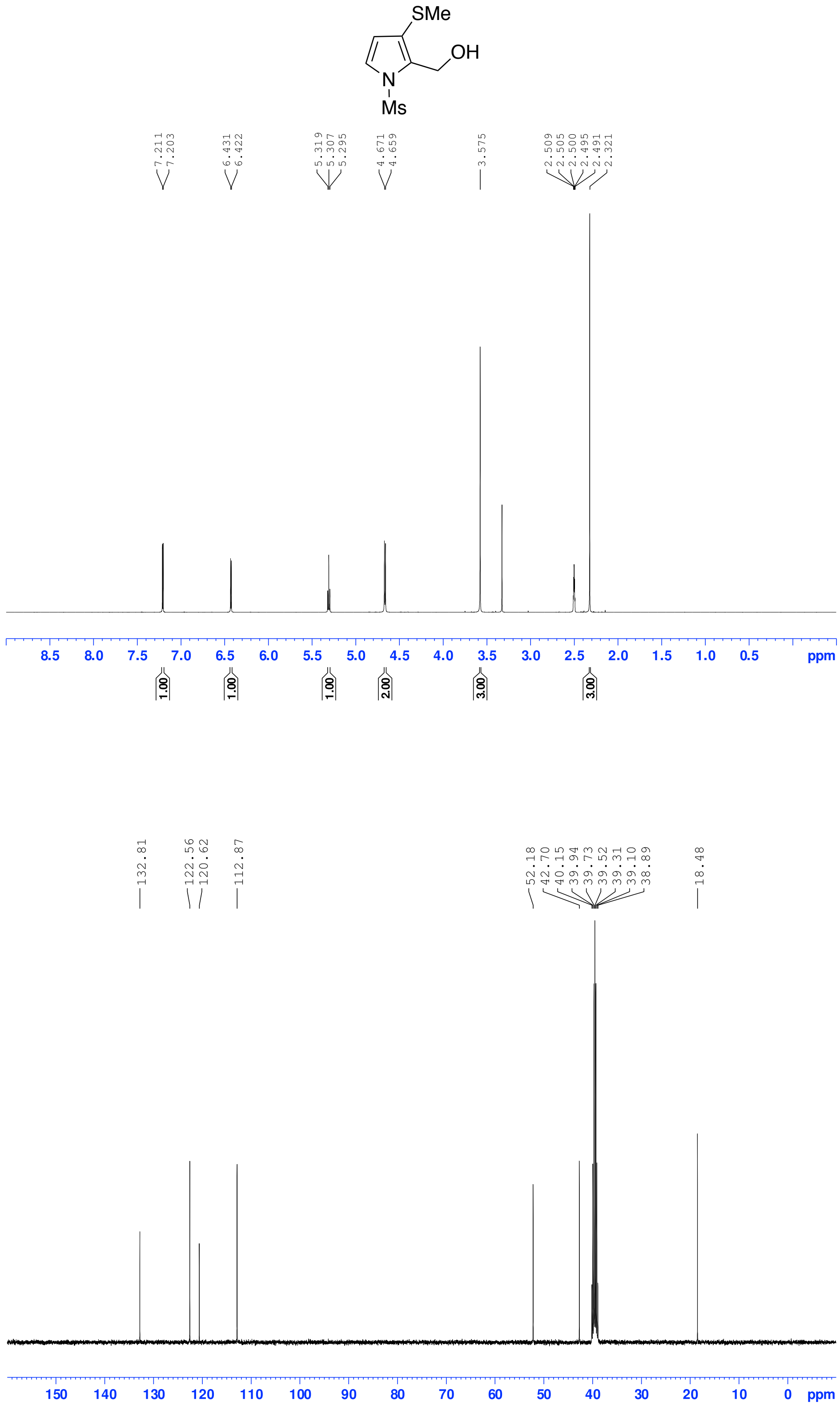

Figure S5. ^{1}H (top) and ^{13}C (bottom) NMR spectra of compound **7** in DMSO-d_6.

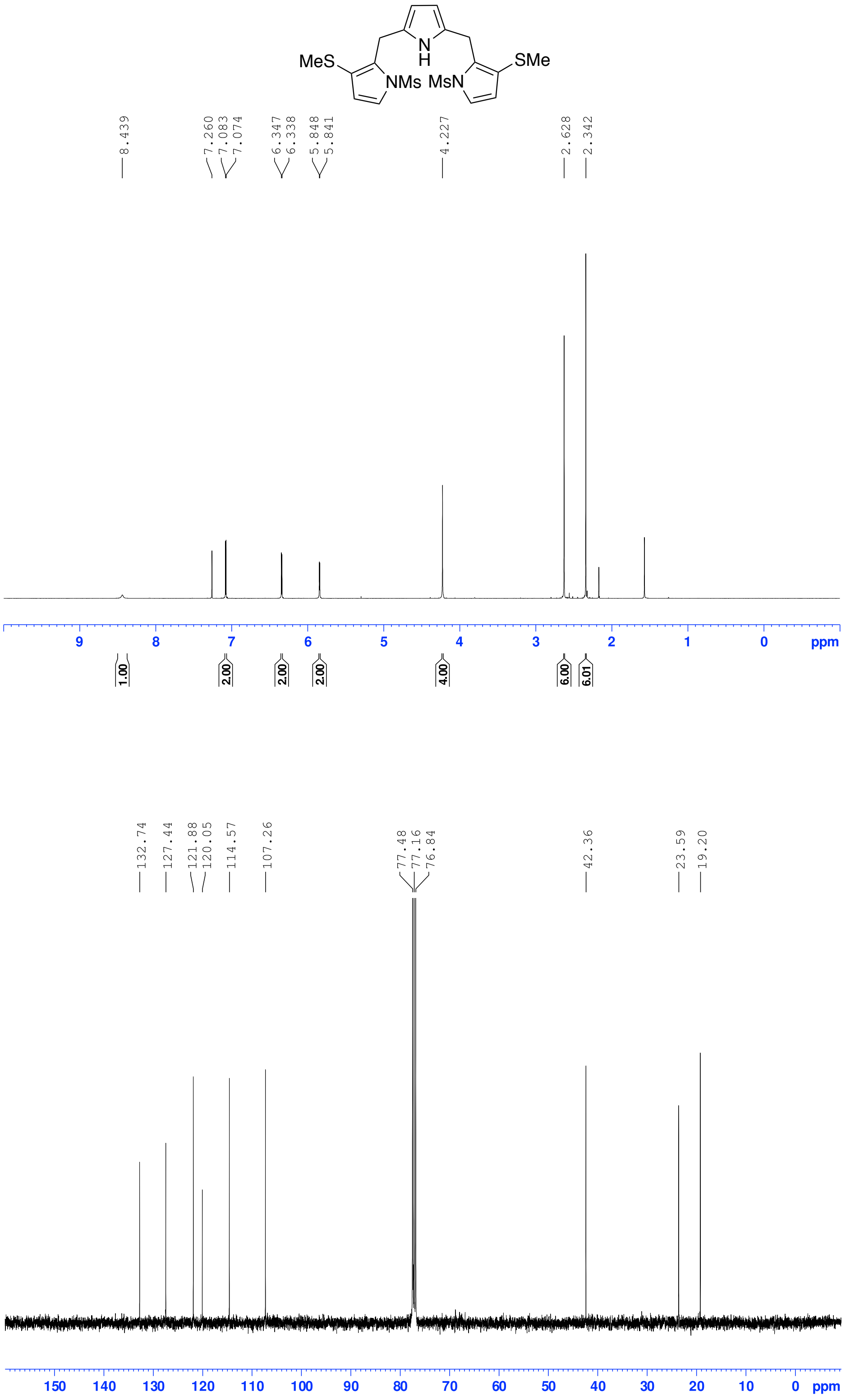

Figure S6. ^{1}H (top) and ^{13}C (bottom) NMR spectra of compound **8** in $CDCl_3$.

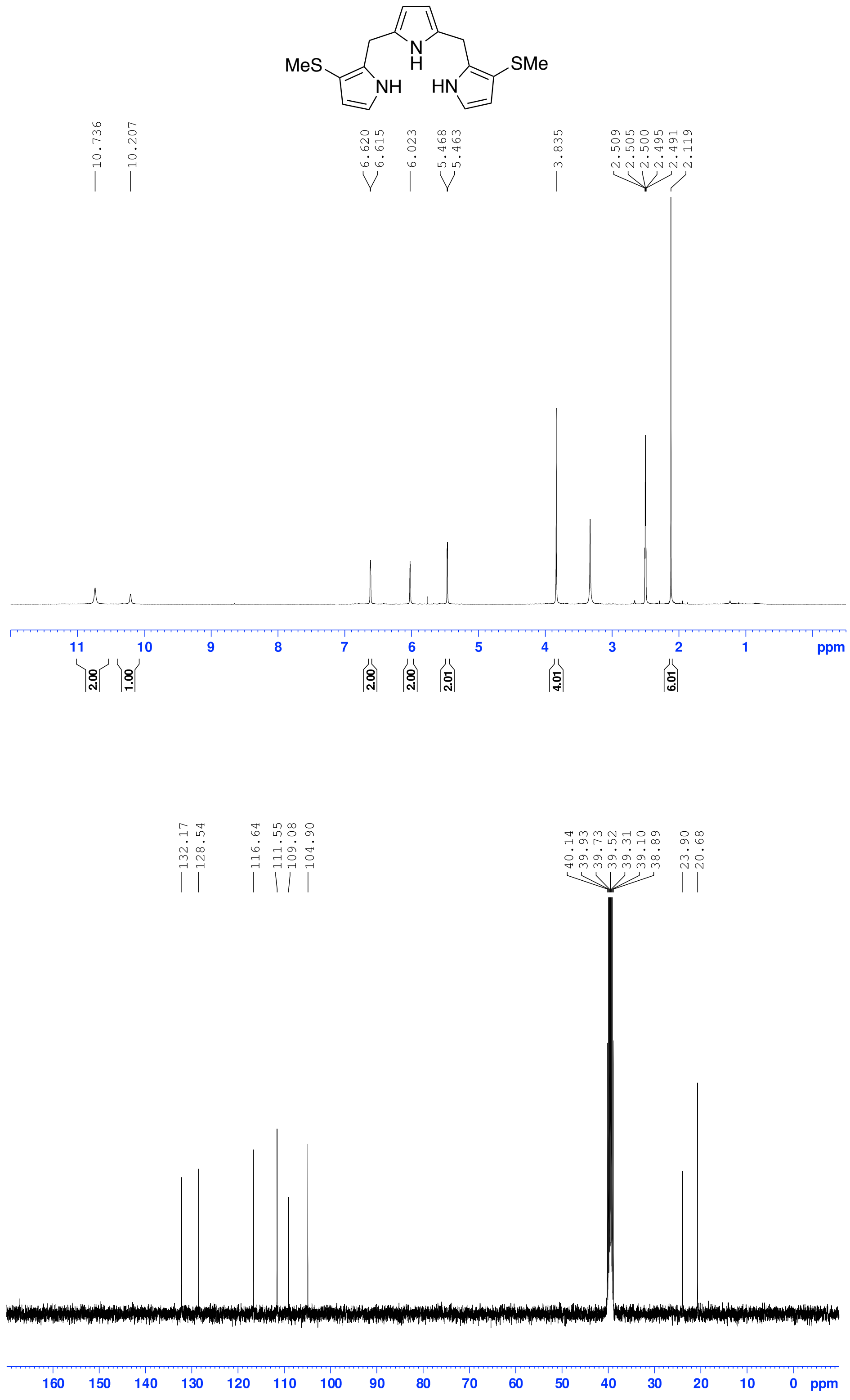

Figure S7. ^{1}H (top) and ^{13}C (bottom) NMR spectra of compound **9** in DMSO-d_6.

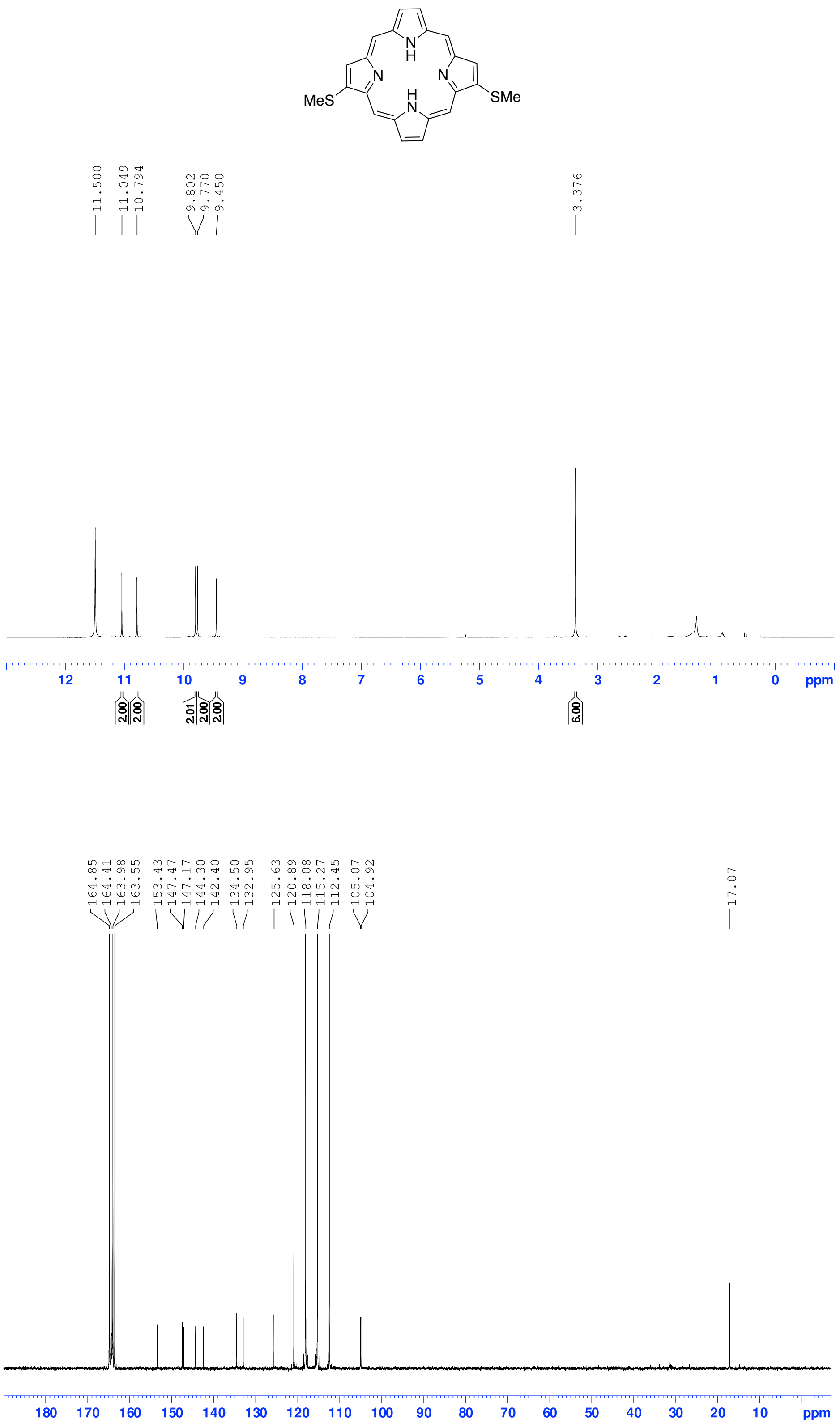

Figure S8. ^{1}H (top) and ^{13}C (bottom) NMR spectra of compound **P1** in TFA-*d*.

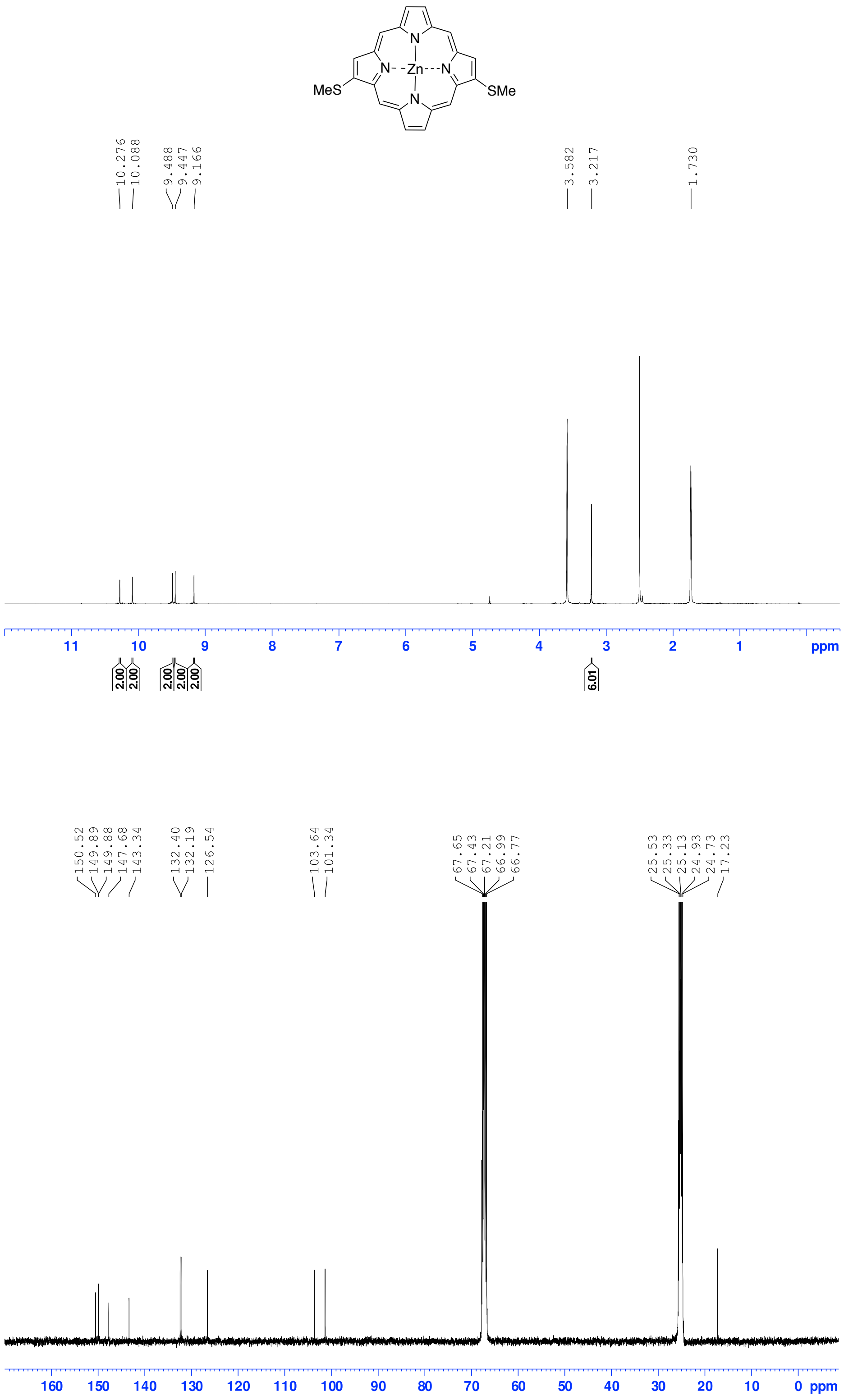

Figure S9. ^{1}H (top) and ^{13}C (bottom) NMR spectra of compound **Zn-P1** in THF-d_8.

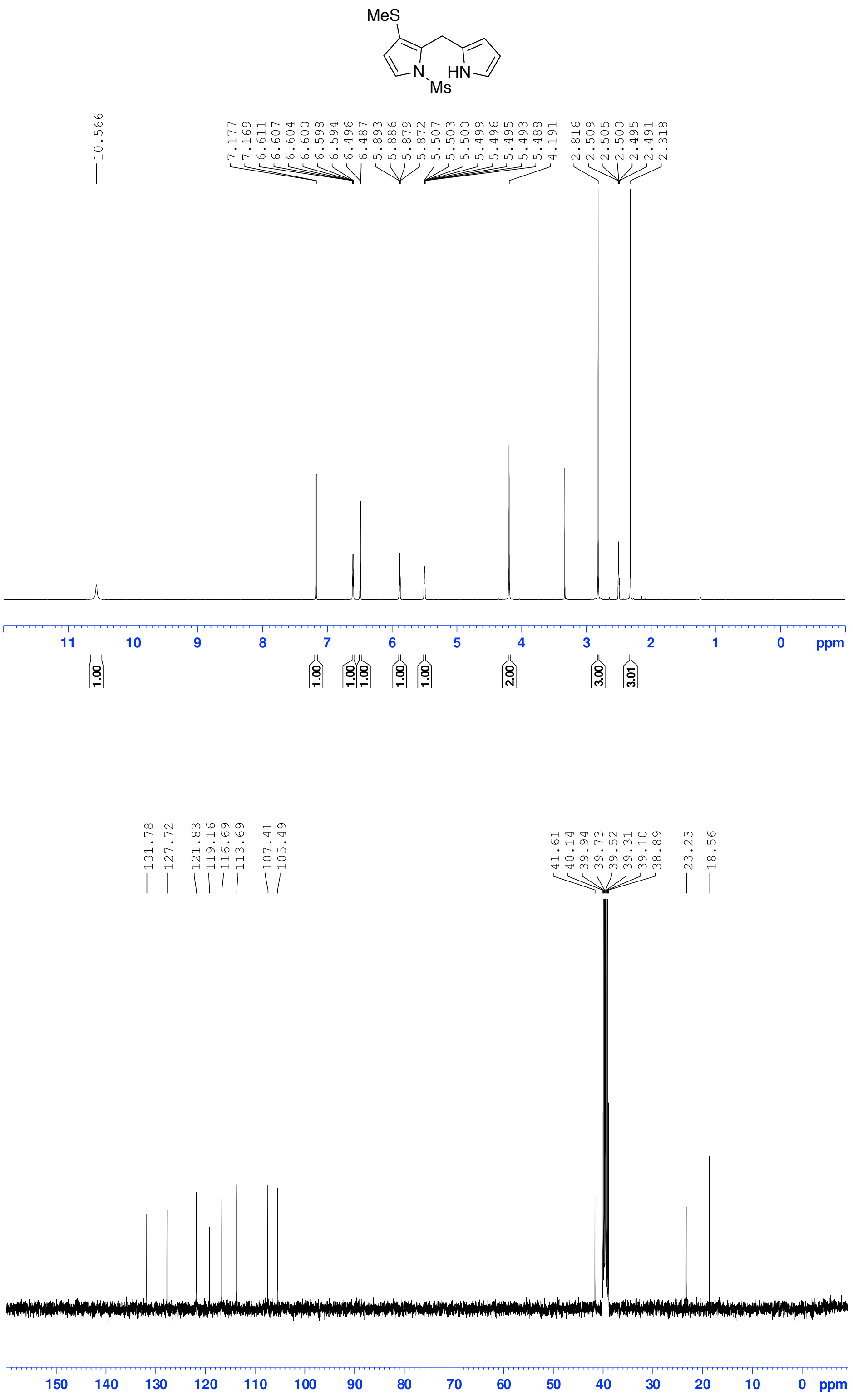

Figure S10. ^{1}H (top) and ^{13}C (bottom) NMR spectra of compound **12** in DMSO-d_6.

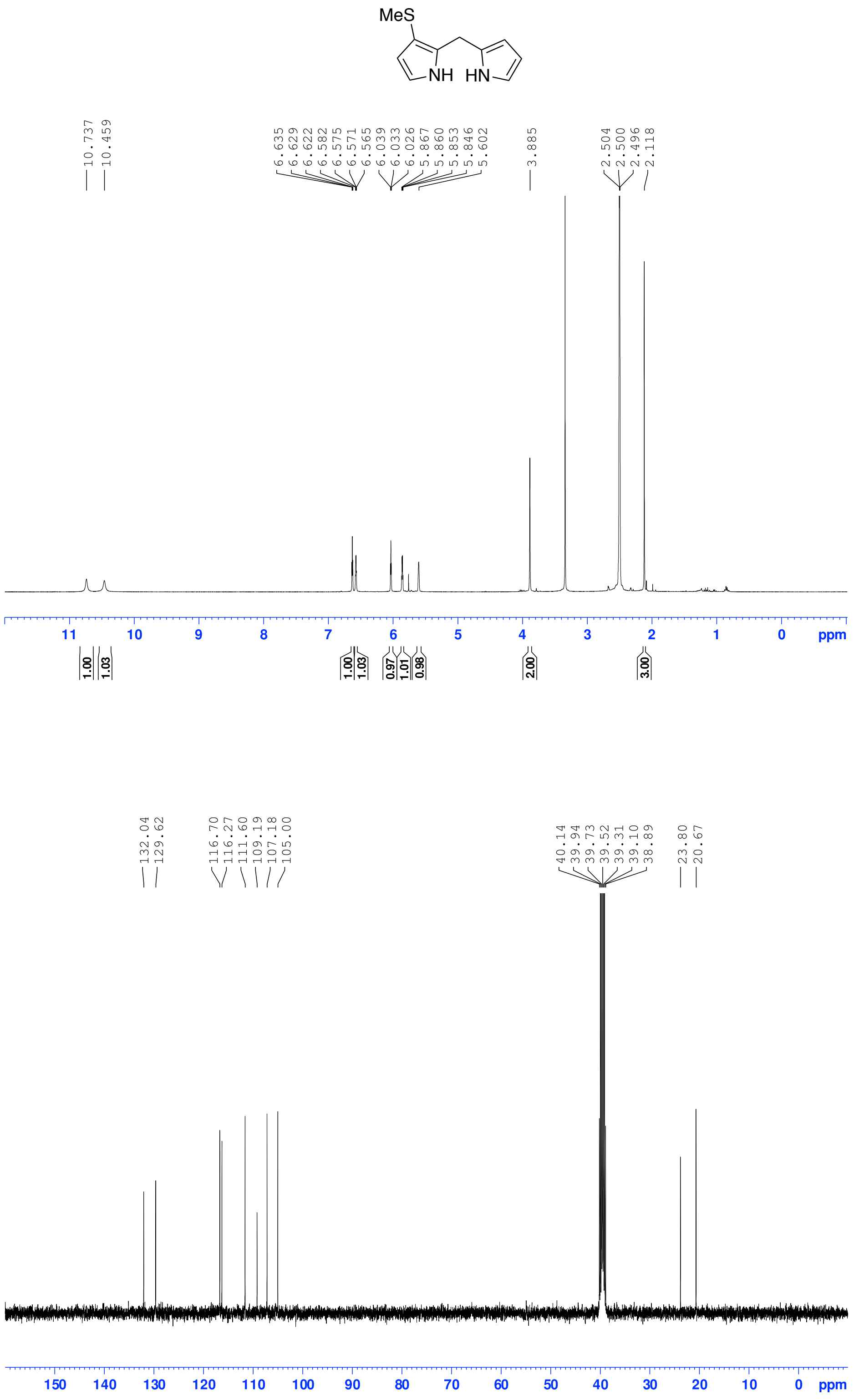

Figure S11. ^{1}H (top) and ^{13}C (bottom) NMR spectra of compound **13** in DMSO-d_6.

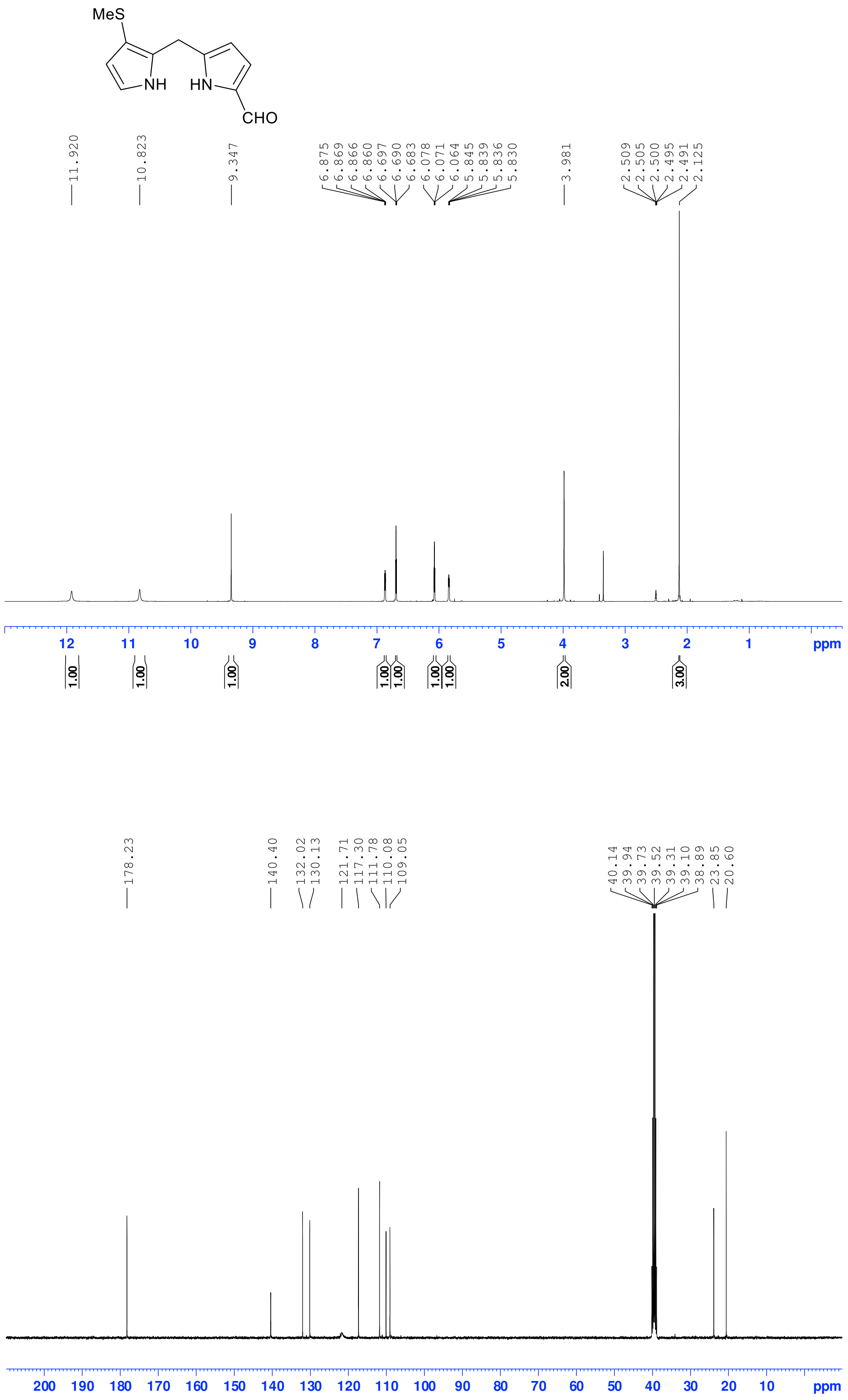

Figure S12. ^{1}H (top) and ^{13}C (bottom) NMR spectra of compound **14** in DMSO-d_6.

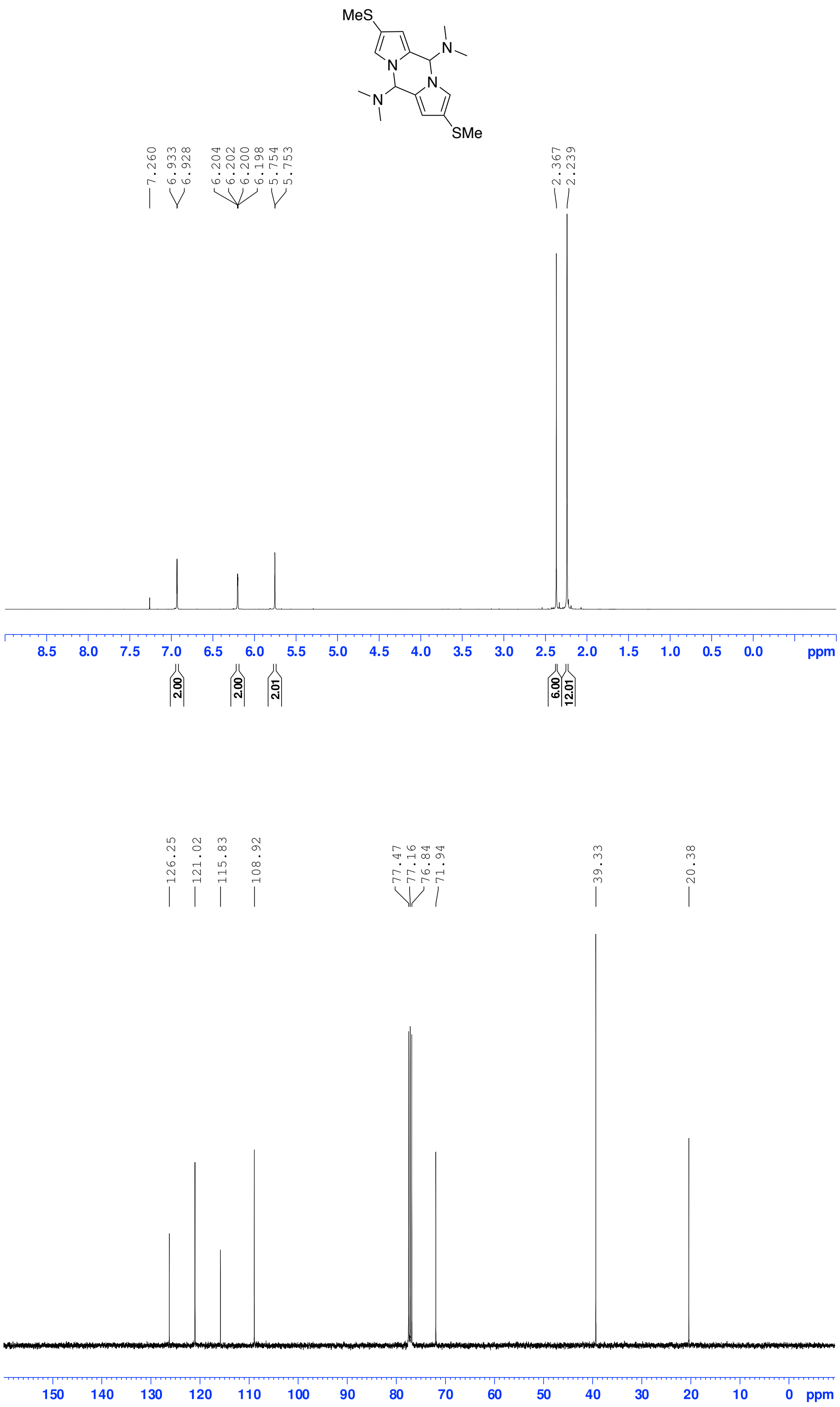

Figure S13. ^{1}H (top) and ^{13}C (bottom) NMR spectra of compound **18** in $CDCl_3$.

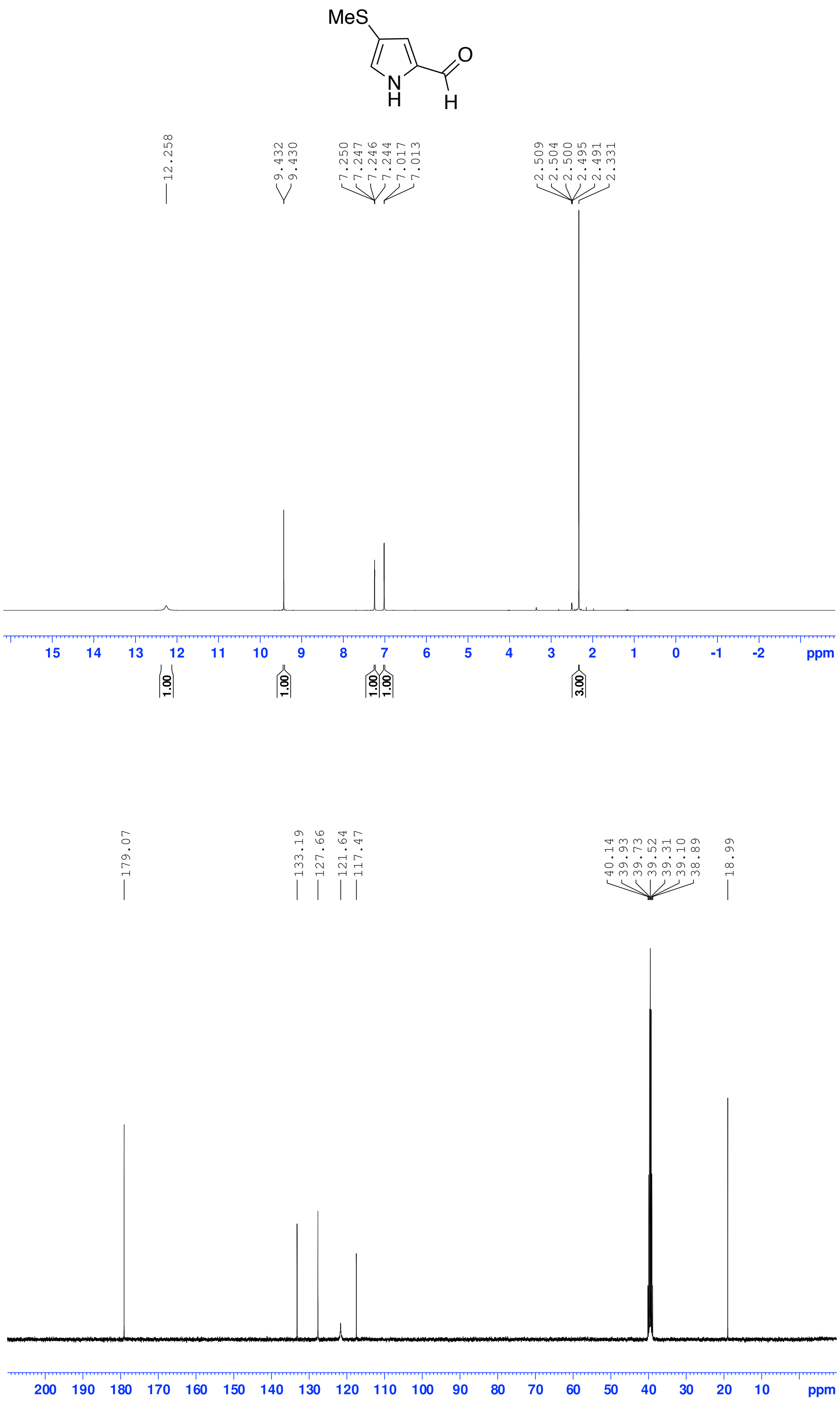

Figure S14. ^{1}H (top) and ^{13}C (bottom) NMR spectra of compound **19** in DMSO-d_6.

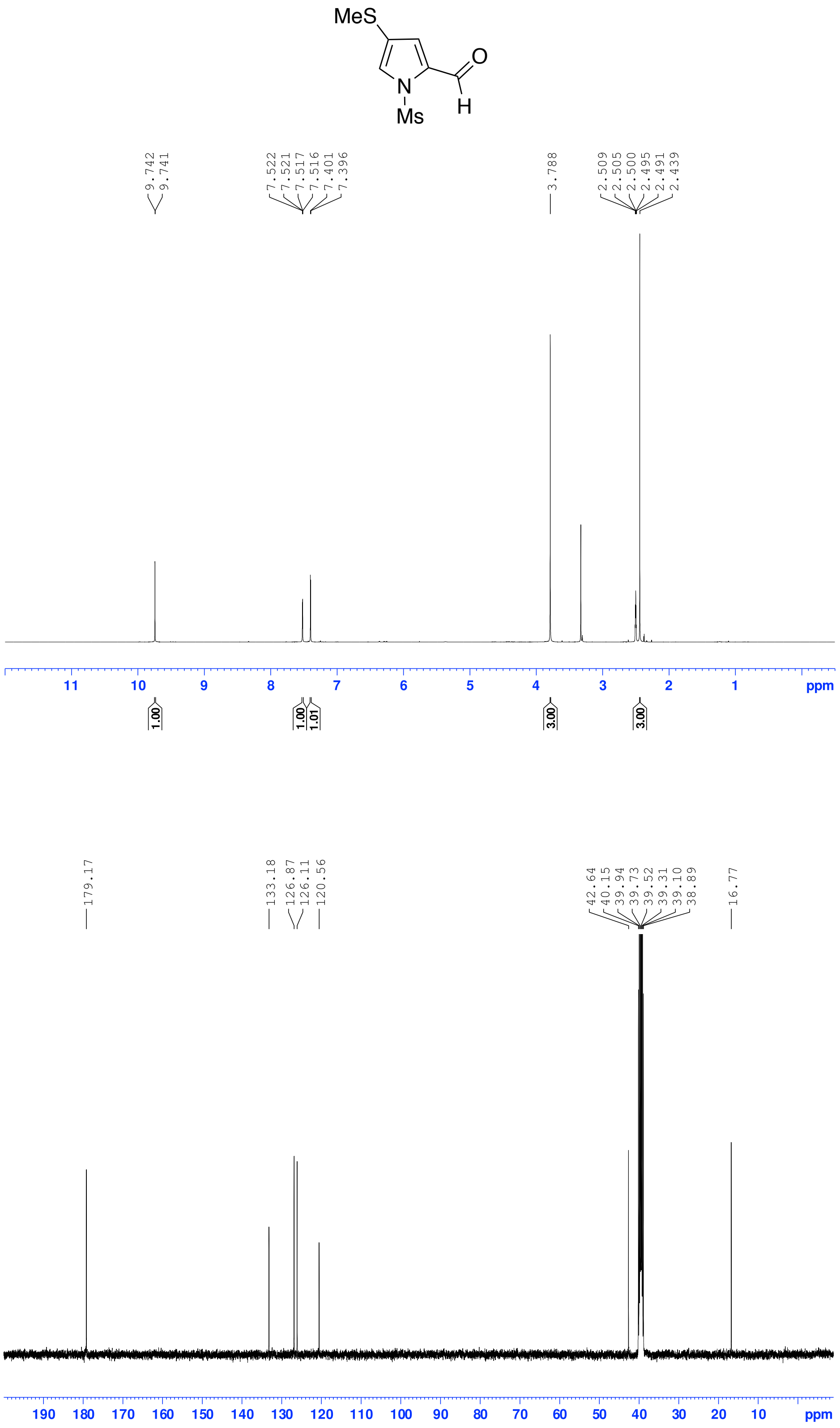

Figure S15. ^{1}H (top) and ^{13}C (bottom) NMR spectra of compound **20** in DMSO-d_6.

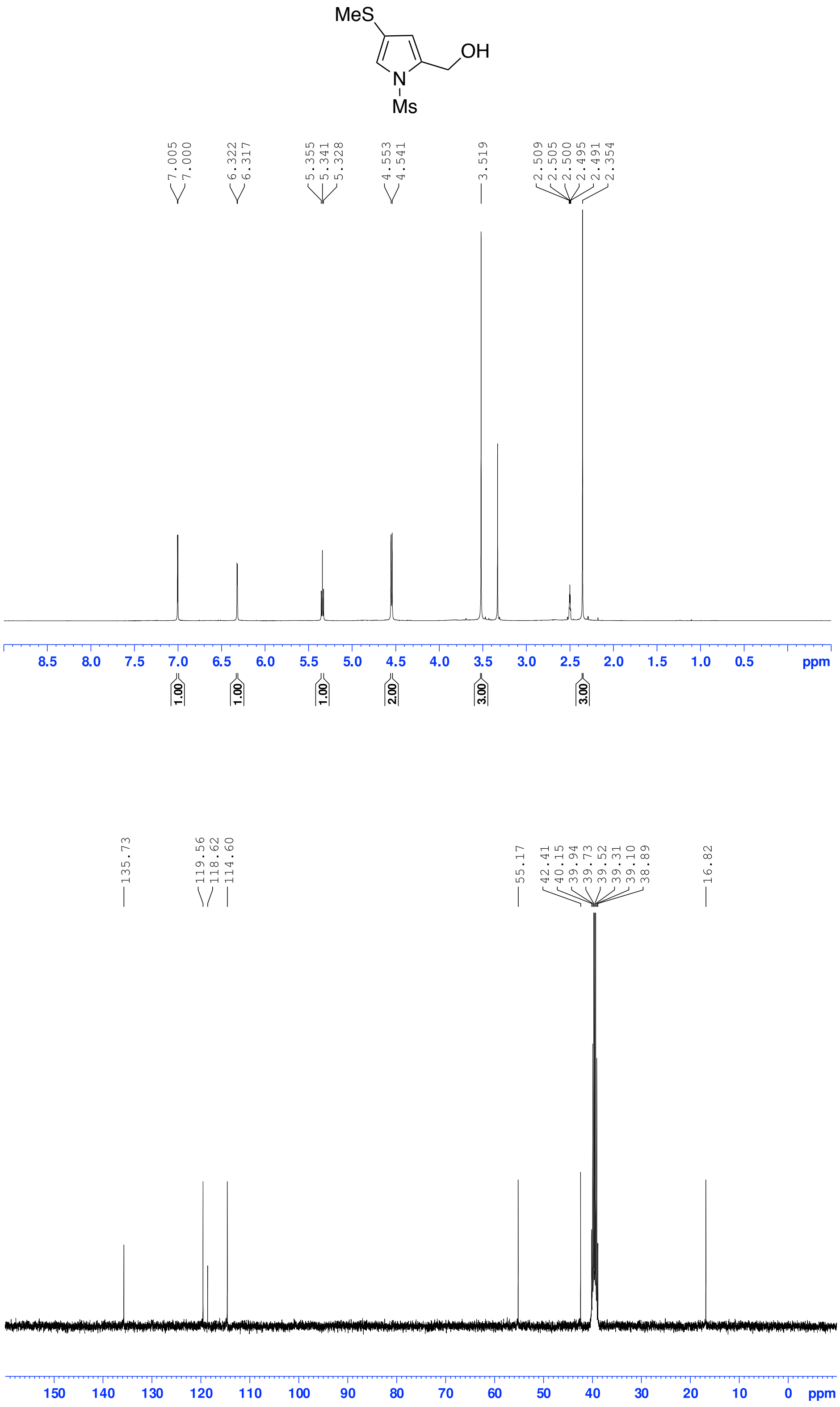

Figure S16. ^{1}H (top) and ^{13}C (bottom) NMR spectra of compound **21** in DMSO-d_6.

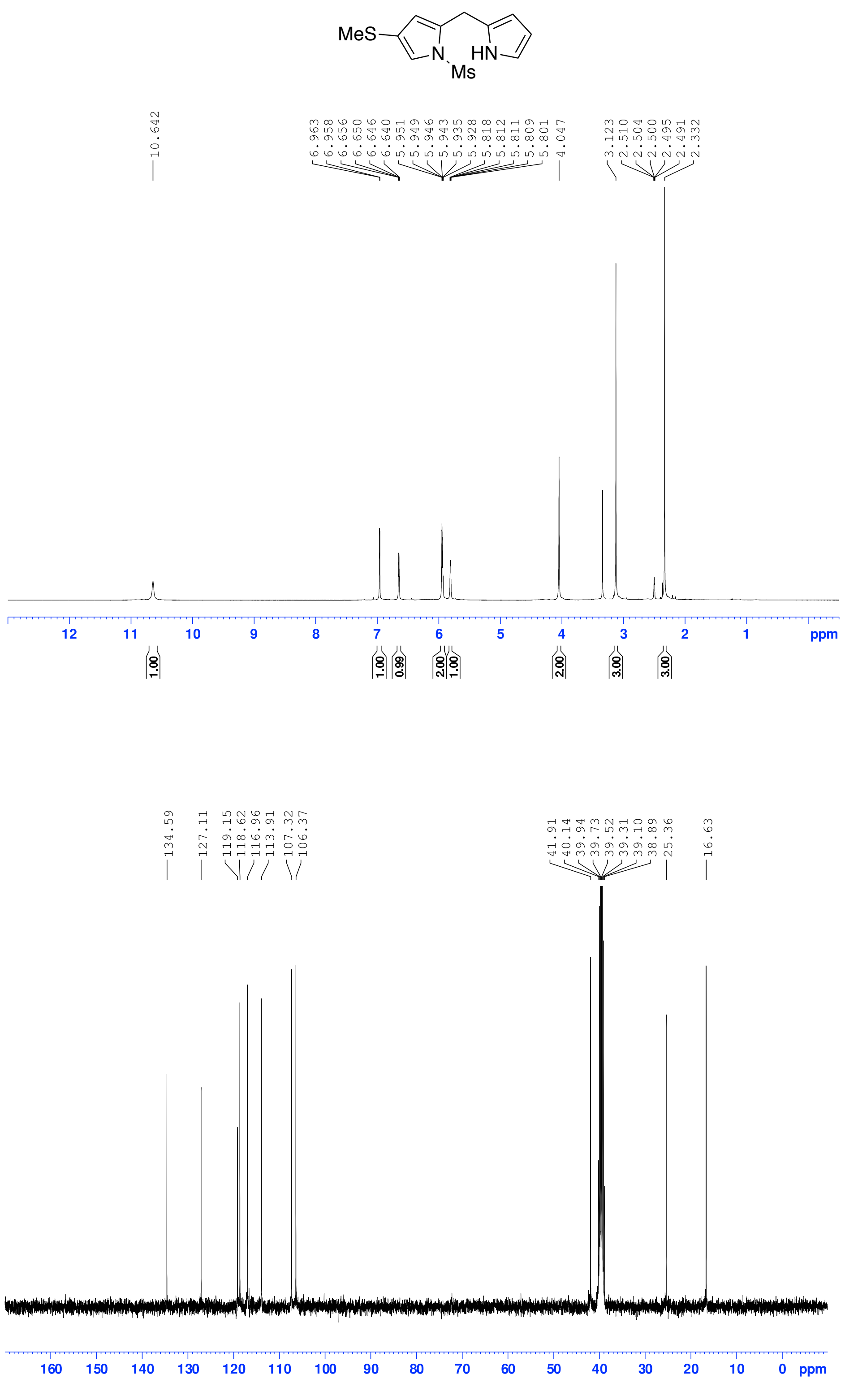

Figure S17. ^{1}H (top) and ^{13}C (bottom) NMR spectra of compound **22** in DMSO-d_6.

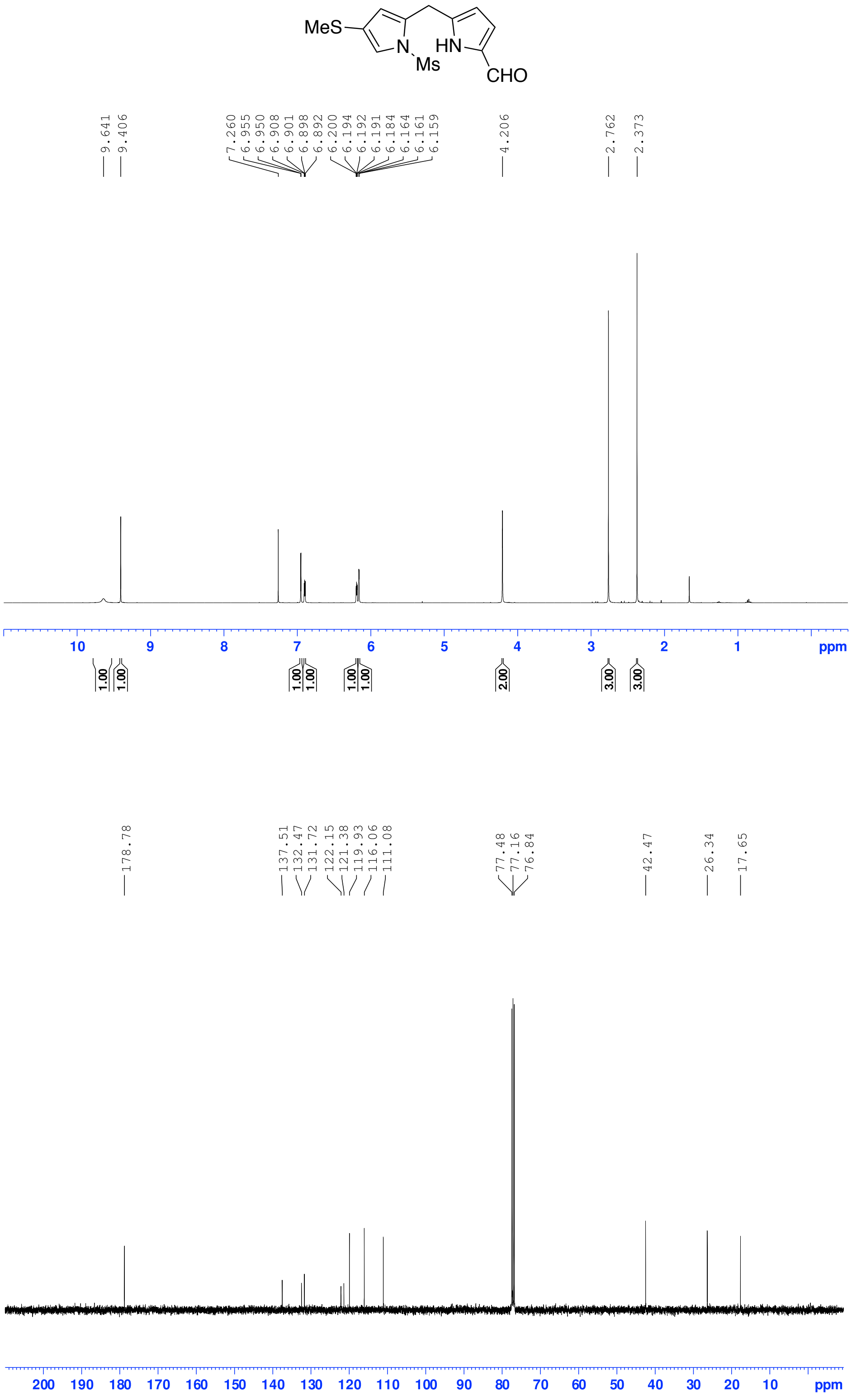

Figure S18. ^{1}H (top) and ^{13}C (bottom) NMR spectra of compound **23** in $CDCl_3$.

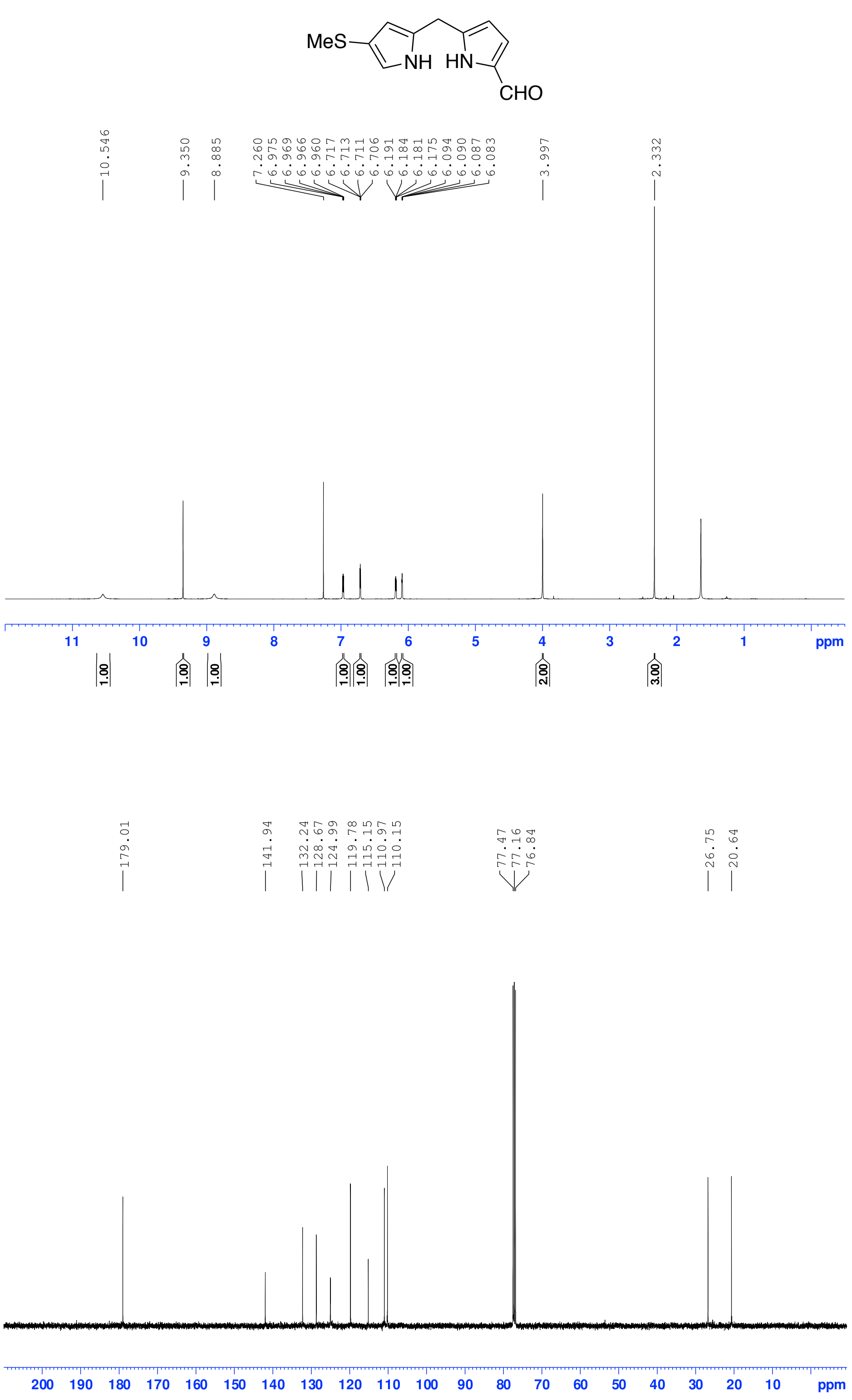

Figure S19. ^{1}H (top) and ^{13}C (bottom) NMR spectra of compound **24** in $CDCl_3$.

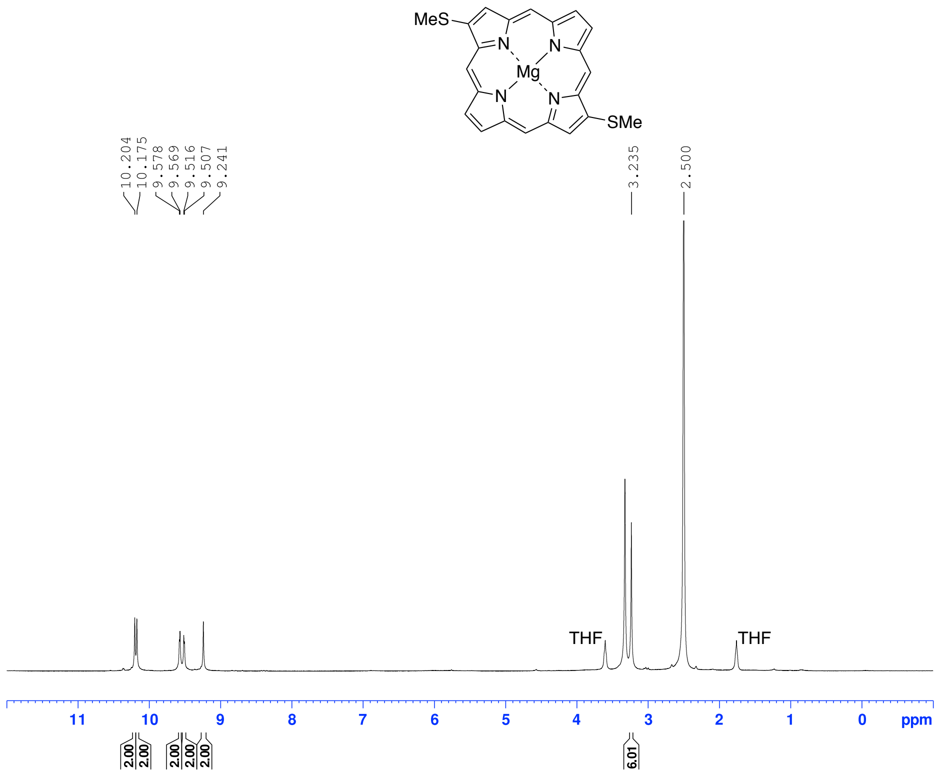

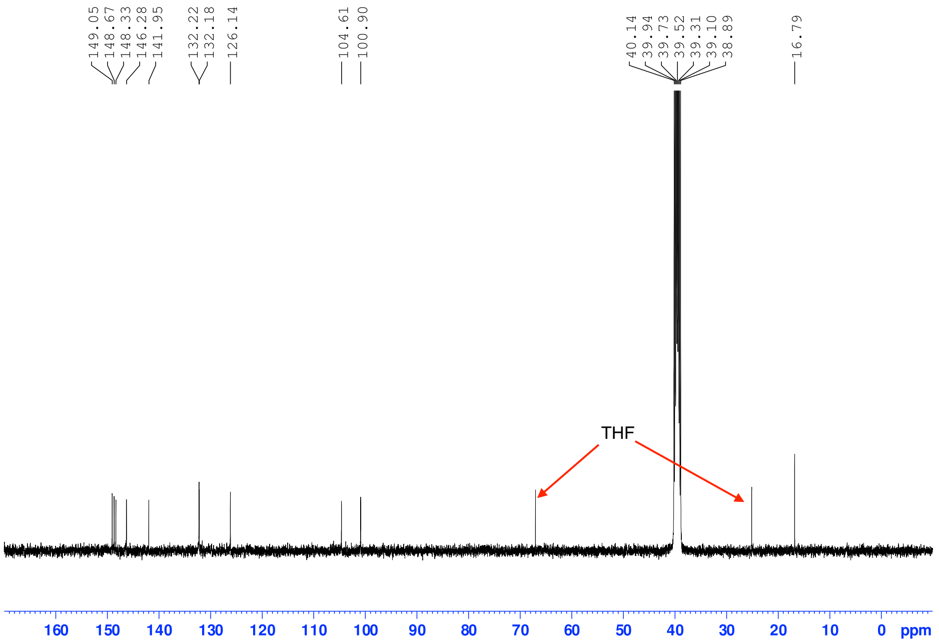

Figure S20. ^{1}H (top) and ^{13}C (bottom) NMR spectra of compound **Mg-P2** in DMSO-d_6.

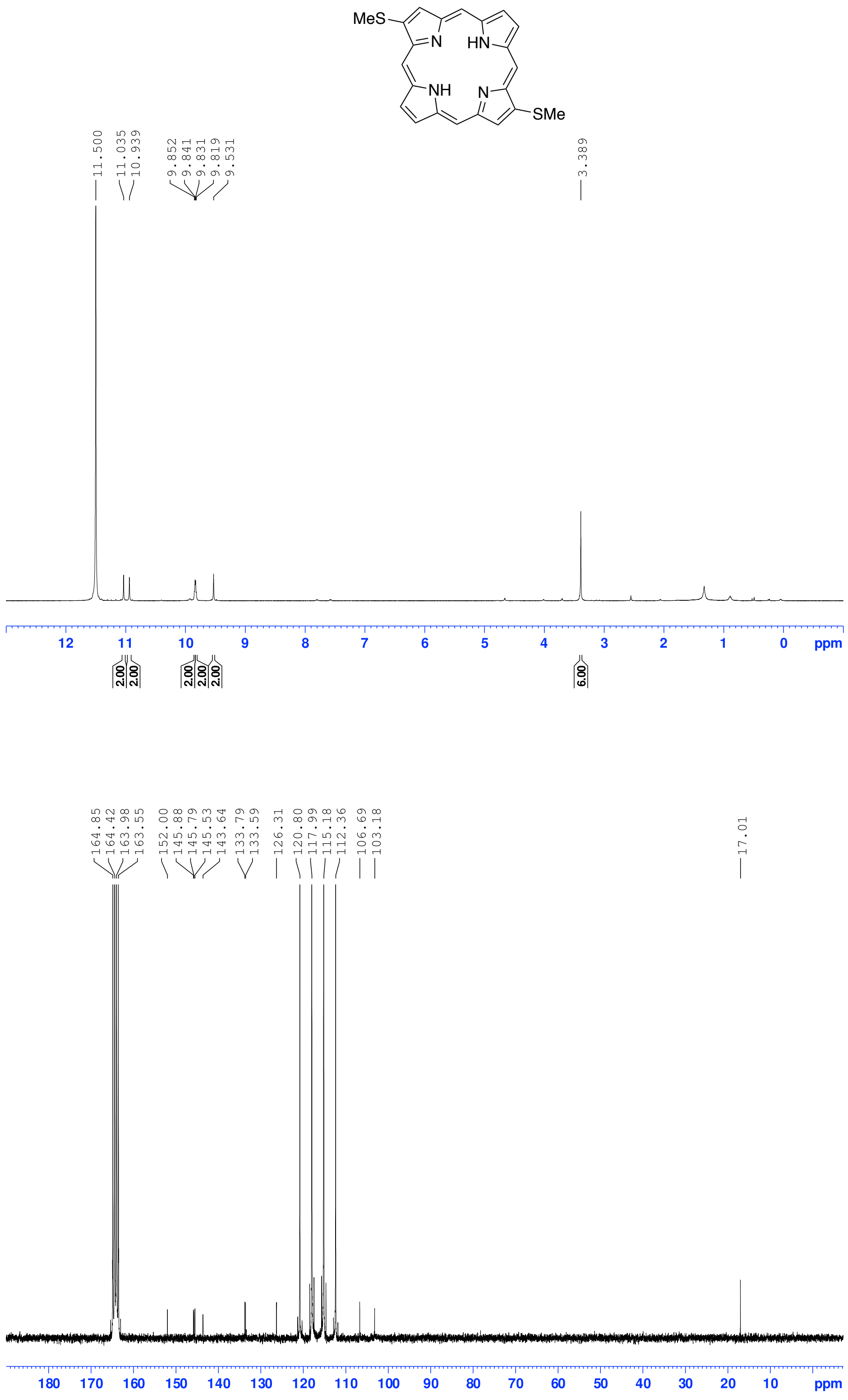

Figure S21. ^{1}H (top) and ^{13}C (bottom) NMR spectra of compound **P2** in TFA-*d*.

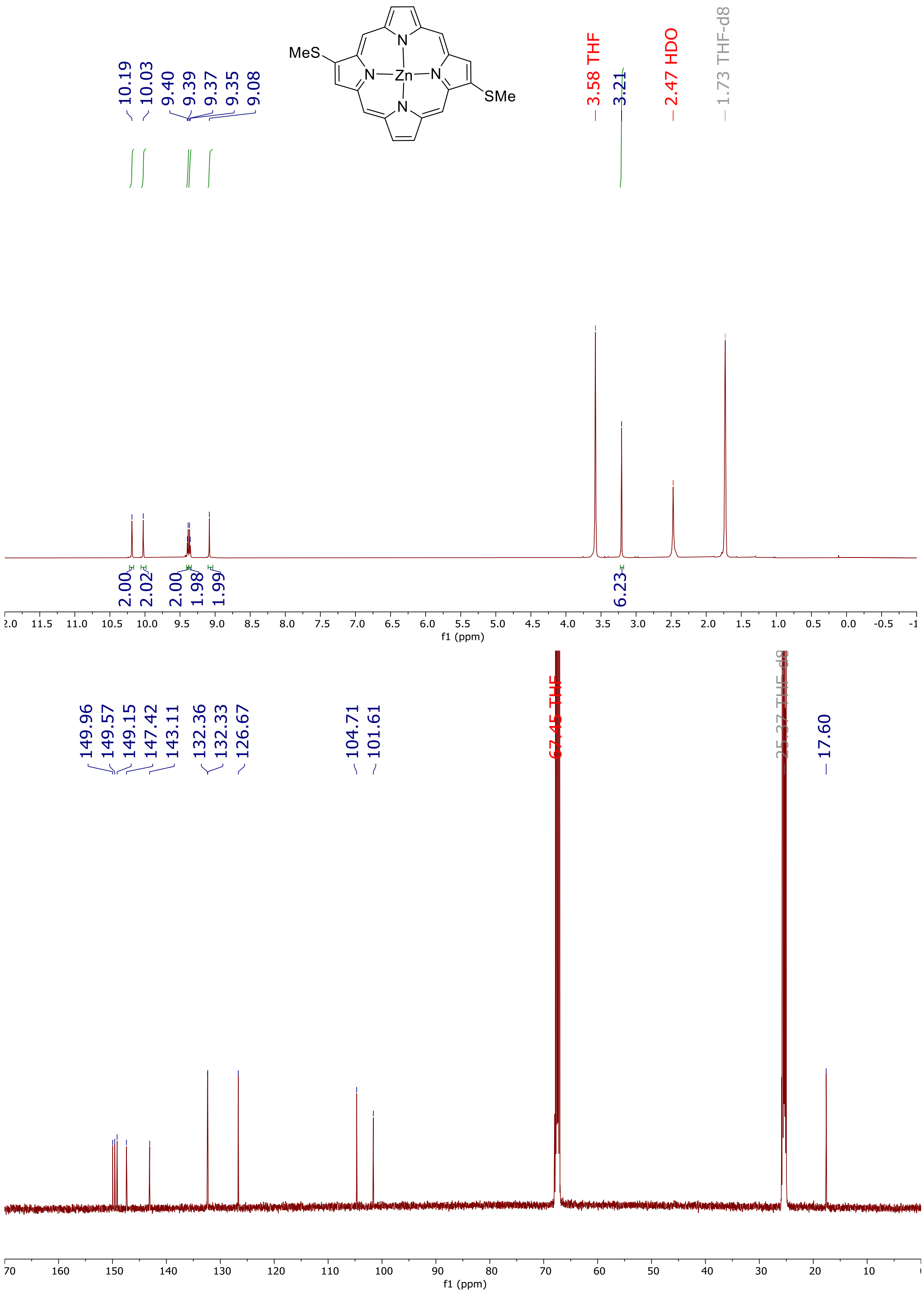

Figure S22. ^{1}H (top) and ^{13}C (bottom) NMR spectra of compound **Zn-P2** in THF-d_8.

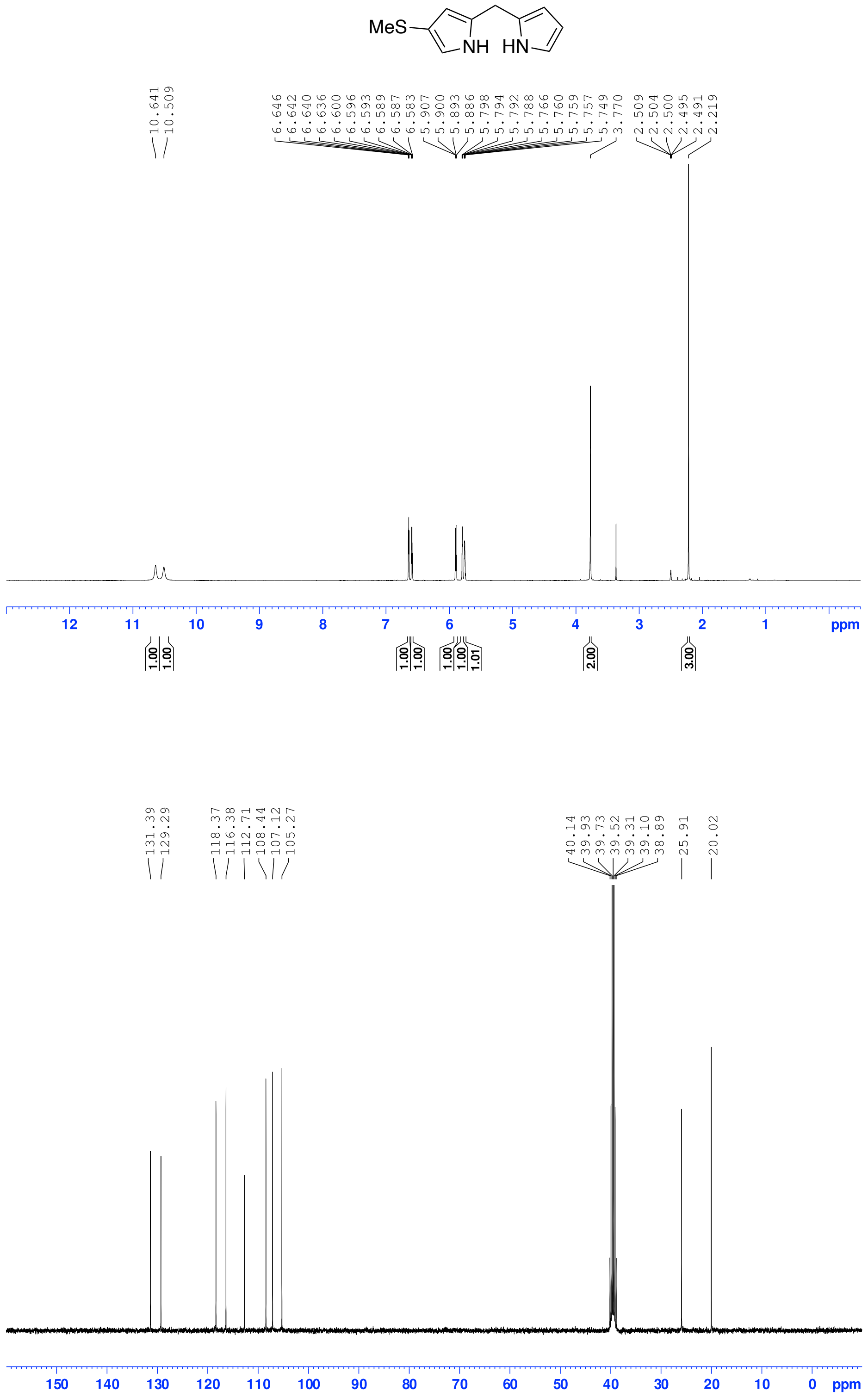

Figure S23. ^{1}H (top) and ^{13}C (bottom) NMR spectra of compound **25** in DMSO-d_6.

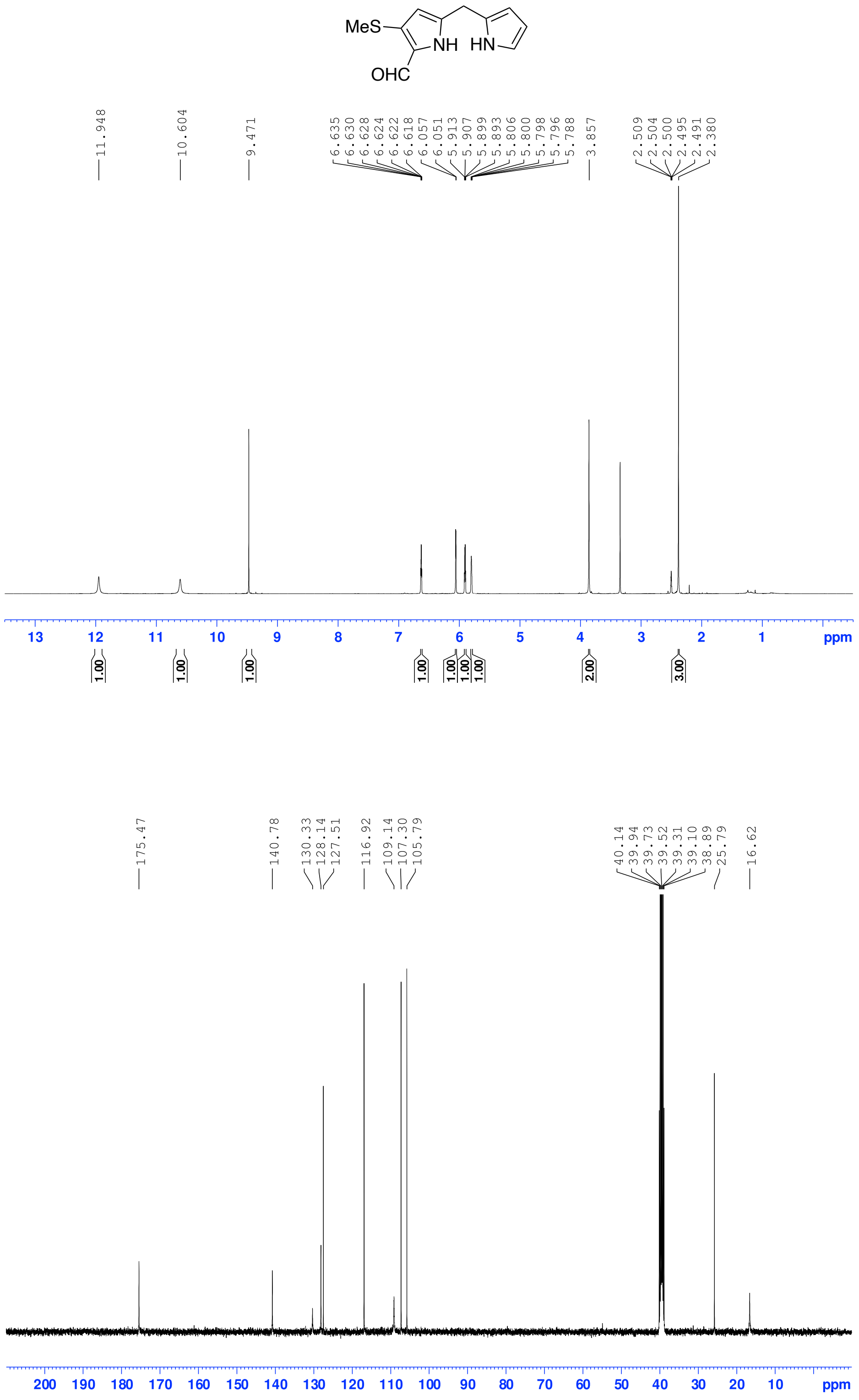

Figure S24. ^{1}H (top) and ^{13}C (bottom) NMR spectra of compound **26** in DMSO-d_6.

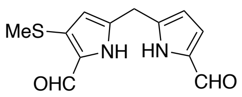

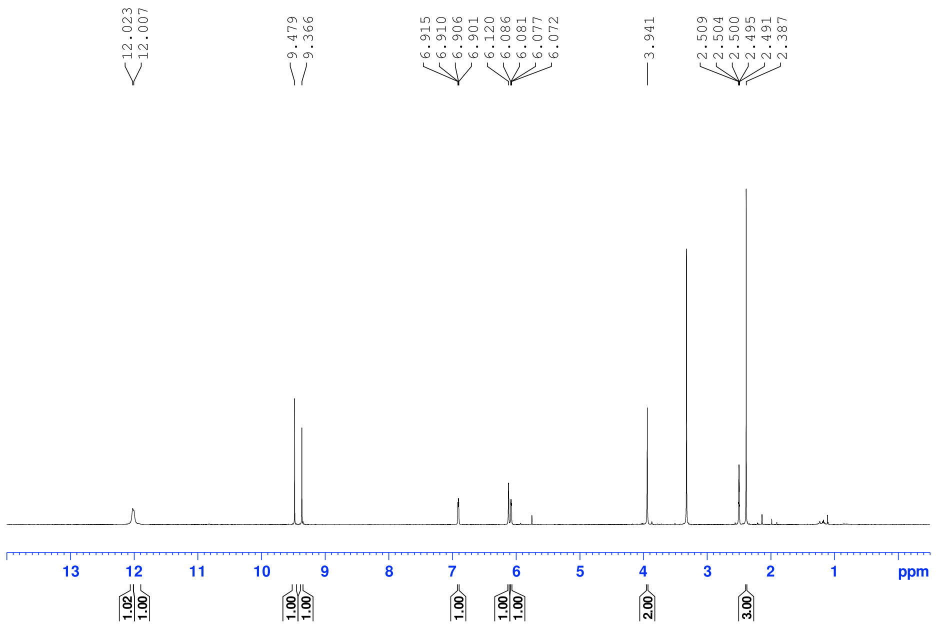

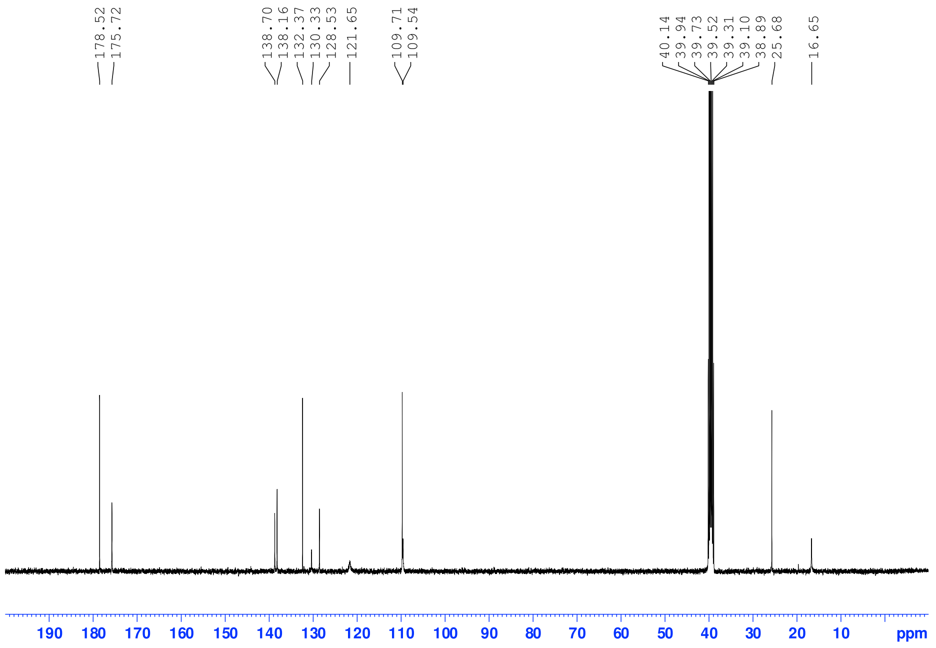

Figure S25. ^{1}H (top) and ^{13}C (bottom) NMR spectra of compound **27** in DMSO-d_6.

Characterization of the porphyrins by UV/VIS spectroscopy: The absorption spectra of the porphyrins **P1**, **Zn-P1**, **P2** and **Zn-P2** in toluene are shown in Figure S26. The spectrum of **P1** shows the typical porphyrin B and Q absorption bands at 385 and 510, 540, 580, 630 nm, respectively. The absorption spectrum of **Zn-P1** displays the B and Q absorption bands at 417 and 543, 579 nm, respectively. Compared to the spectrum of **P1**, the absorption of **P2** shows two B bands at 379 and 407 nm, respectively, which is most probably caused by π-π stacking. **Zn-P2** shows similar absorption bands as **Zn-P1**, while the major B band at 418 nm becomes sharper.

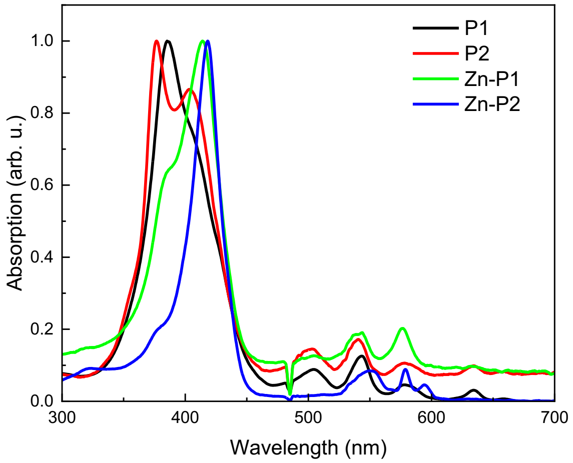

Figure S26. Absorption spectra of porphyrins **P1** (black line), **P2** (red line), **Zn-P1** (green line) and **Zn-P2** (blue line) at room temperature and at a concentration of 0.1 mM in toluene. The absorption spectra are normalized at the B-band. The dip at 480 nm and spectra offset are due to instrument errors.

Part 4. Electronic structure of isolated molecules

Let us first study the electronic structure of the isolated molecules. Beside the four molecules **P1**, **P2**, **Zn-P1** and **Zn-P2**, we consider the pristine porphyrin, **P0**, and Zn-porphyrin, **Zn-P0**, without the SMe anchoring groups. The geometries of **P0** and **Zn-P0** are shown in Figure S27. Of special interest are the highest occupied molecular orbital (HOMO) and the lowest unoccupied molecular orbital (LUMO), as well as the size of the electronic HOMO-LUMO gap. In Tables S3 to S5 the corresponding values are shown for all four molecules using three different methods, namely density functional theory (DFT), ΔSCF and G_0W_0.

On the technical side, all calculations of the isolated molecules were performed with the TURBOMOLE quantum chemistry software package.[S6] As the basis set, we used def-TZVP.[S7] Molecular ground state geometries were determined with DFT by minimizing total energies (energy convergence criterion "scfconv 8", geometry convergence criterion "gcart 3"). As exchange-correlation functional for the DFT

calculations we chose PBE.[S8] Calculations with ΔSCF and G_0W_0 use PBE geometries.

The DFT results in Table S3 show that the position of the sulfur atoms and the connected methylene groups on both sides basically do not affect the energy of the frontier molecular orbitals. The Zn in the center of the porphyrin ring of **Zn-P1** and **Zn-P2** causes a slight decrease of HOMO energies as well as a small increase of the LUMO energies. In this way, the electronic gap size increases by around 0.1 eV. A similar effect can already be seen in the comparison between the **P0** and **Zn-P0** molecules, meaning that the Zn atom does not introduce any relevant electronic states inside the HOMO-LUMO gap.

Since DFT tends to underestimate electronic gaps of molecules,[S9] we employ the ΔSCF method to get a more reliable estimate of frontier molecular orbital energies. This method uses differences in the total energy between the neutral and charged molecules to calculate the electron affinity (EA) and the ionization potential (IP), which correspond to the negative of HOMO and LUMO energies, respectively.[S9] The list in Table S4 for all molecules shows the same trends as already described for the DFT values. Quantitatively, HOMO energies are however reduced by around 1.5 eV and LUMO energies increased by a similar amount, such that HOMO-LUMO gaps increase by nearly 3.3 eV.

Another popular method to compute electronic quasiparticle energies is the *GW* approximation, which is based on many body perturbation theory and the Hedin equations.[S10,S11,S12] Results that we obtained by using the non-self-consistent G_0W_0 correction are listed in Table S5. As compared to the DFT calculations, we find a substantial gap opening. However, the HOMO-LUMO gaps are smaller than ΔSCF results by around 0.5 eV. This originates mainly from HOMO states that are increased by around 0.5 eV as compared to ΔSCF.

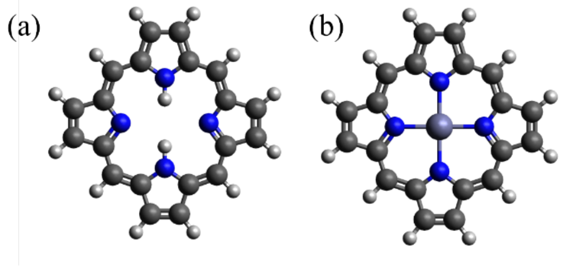

Figure S27. Geometries of (a) **P0** and (b) **Zn-P0**, i.e., the studied porphyrins without SMe anchoring groups.

Table S3. Calculated frontier molecular levels and HOMO-LUMO energy gaps using DFT for molecules **P0, Zn-P0, P1**, **P2**, **Zn-P1** and **Zn-P2**.

Molecule	HOMO (eV)	LUMO (eV)	Energy gap (eV)
**P0**	-5.01	-3.07	1.94
**Zn-P0**	-5.05	-2.99	2.07
**P1 (|)**	-5.11	-3.23	1.88
**P1 (-)**	-5.11	-3.22	1.89
**P2 (|)**	-5.10	-3.23	1.87
**P2 (-)**	-5.11	-3.23	1.88
**Zn-P1**	-5.17	-3.15	2.01
**Zn-P2**	-5.17	-3.16	2.01

Table S4. Calculated frontier molecular levels and HOMO-LUMO energy gaps using ΔSCF based on DFT for molecules **P0, Zn-P0, P1**, **P2**, **Zn-P1** and **Zn-P2**.

Molecule	-EA (eV)	-IP (eV)	Energy gap (eV)
**P0**	-6.78	-1.33	5.45
**Zn-P0**	-6.82	-1.25	5.57
**P1 (|)**	-6.73	-1.52	5.22
**P1 (-)**	-6.78	-1.59	5.19
**P2 (|)**	-6.69	-1.61	5.08
**P2 (-)**	-6.77	-1.61	5.16
**Zn-P1**	-6.75	-1.44	5.31
**Zn-P2**	-6.79	-1.54	5.25

Table S5. Calculated frontier molecular levels and HOMO-LUMO energy gaps using G_0W_0 based on DFT for molecules **P0, Zn-P0, P1**, **P2**, **Zn-P1** and **Zn-P2**.

Molecule	HOMO (eV)	LUMO (eV)	Energy gap (eV)
**P0**	-6.16	-1.32	4.84
**Zn-P0**	-6.22	-1.22	4.99
**P1 (|)**	-6.25	-1.54	4.71
**P1 (-)**	-6.20	-1.55	4.65
**P2 (|)**	-6.29	-1.54	4.75
**P2 (-)**	-6.20	-1.55	4.65
**Zn-P1**	-6.27	-1.48	4.79
**Zn-P2**	-6.28	-1.48	4.80

Part 5. Experimental techniques

Sample preparation

Gold (Au) STM tips were fabricated by an electrochemical etching method. Briefly, we first prepared an etchant solution by mixing 25 ml of 37% hydrochloric acid (hydrochloric acid, certified ACS Plus, Fisher Chemical™) with an equal volume of absolute ethanol (200 proof, USP, Decon™ Labs). Then, a ~1 cm long Au wire and a 6 mm diameter Au circular ring were prepared from a gold wire (Alfa Aesar™ gold wire, 0.25 mm dia., annealed, Premion™, 99.999%), which served as anode and cathode, respectively. The Au ring was placed at the surface of the etchant solution, and the Au wire to be etched was located perpendicular to the plane of the Au circular ring with its end ~1 mm below the solution surface. Next, a 7 V DC bias was applied between the anode (Au wire) and cathode (Au ring) to drive the electrochemical etching process. Finally, the etching process was stopped manually immediately after the Au wire was etched and broke at the meniscus of the solution, leaving a fresh and sharp Au STM tip at the end of the wire.

Template stripped samples that were used in the experiments were prepared by first depositing 100 nm Au on a pristine Si wafer (100 mm silicon wafer, 500 μm thick, SSP Prime), which was pre-cleaned in a Piranha solution for 20 mins to remove any residual particulates. Subsequently, 7 mm × 7 mm square glass pieces (diced from a 500 μm thick, 100 mm Borofloat 33 glass wafer, double side polished) were attached to the Au-coated silicon wafer by using a small drop of epoxy (EPO-TEK® 377). To prepare a sample for our measurement, we stripped off one of the glass pieces from the Au-coated silicon wafer and used the side where the pristine Au film was originally facing the silicon wafer as the Au surface for forming a molecular monolayer.

To form a layer of molecules on the Au substrate, a 0.5 mM solution of test molecules in dichloromethane solvent was drop-casted onto the freshly prepared Au template stripped sample for 3 hours to allow the adsorption of a layer of molecules. Subsequently, the prepared Au film covered with a layer of test molecules was carefully rinsed with ethanol and dried with high-purity nitrogen gas. The sample was immediately used for single molecule electrical conductance and thermopower measurements.

Electrical conductance measurements

The electrical conductance of single-molecule junctions was investigated using the scanning tunneling microscope break-junction (STM-BJ) technique. The measurements were conducted under ambient conditions (~50% relative humidity) at room temperature. In the STM-BJ measurements, a constant DC voltage bias of 100 mV was applied between an electrochemically etched Au STM tip and a freshly prepared template-stripped Au substrate on which a molecular monolayer was formed. The STM Au tip was first approached towards the Au substrate to make a contact. When a Au-Au contact was detected by the recorded current, the tip was subsequently withdrawn from the Au substrate at a constant speed

of 0.2-0.4 nm/s, during which molecules were trapped between the tip and substrate stochastically. The current (*I*) flowing through the molecular junctions was recorded during the tip retraction process with a low-noise current amplifier (DDPCA-300, FEMTO®). The formation of molecular junctions manifested as plateaus in the electrical conductance traces. The last plateau before junction rupture represents a single molecule junction. In order to determine the most probable single molecule electrical conductance, we collected ~1000 consecutive traces to construct the 1D histograms for all four molecules. The 2D density plots of electrical conductance versus electrode displacement that were generated from these data are shown in Figure S28, while the histograms, built from these data, are shown in Figure 5 of the manuscript along with Gaussian fits to the peaks.

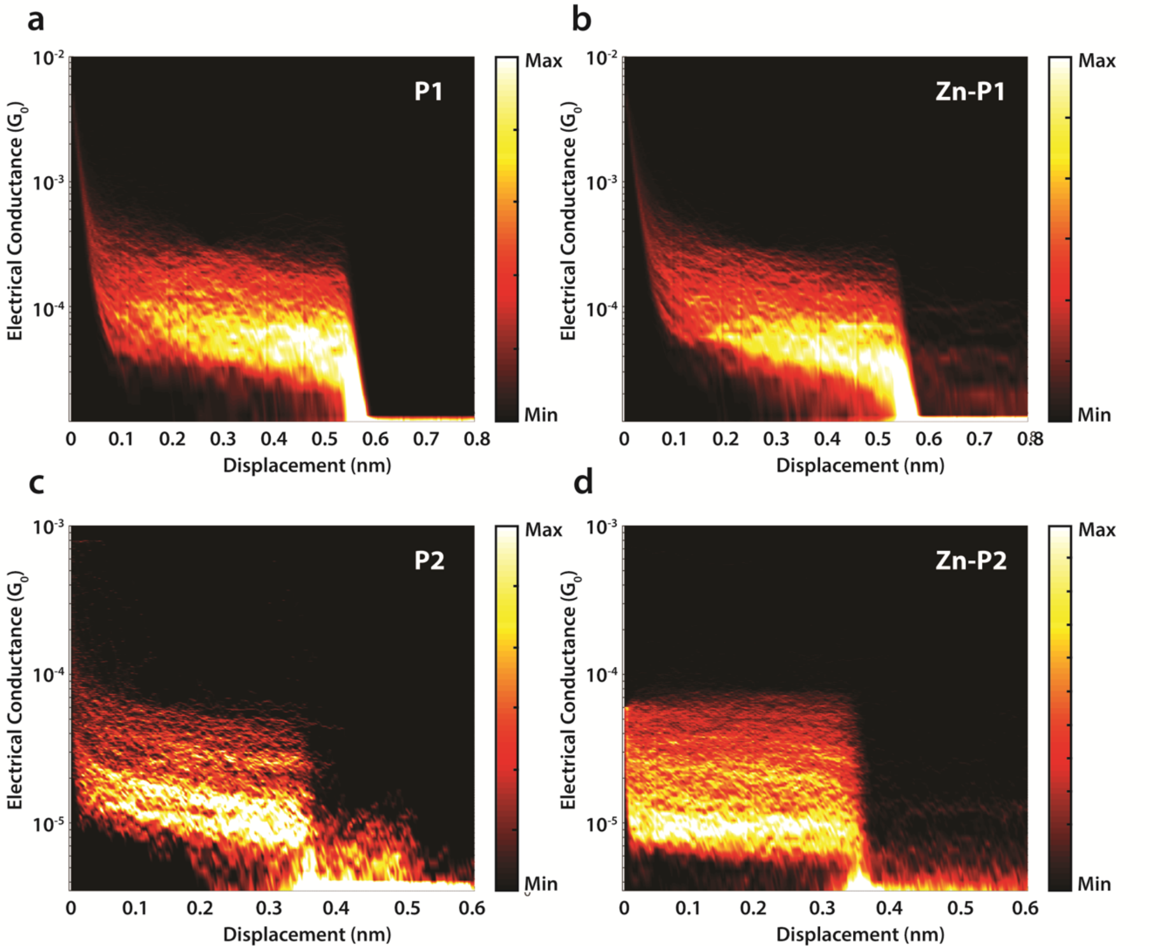

Figure S28. 2D density plots of the measured electrical conductance vs. electrode displacement for (a) **P1**, (b) **Zn-P1**, (c) **P2** and (d) **Zn-P2** based junctions. Each plot is constructed from ~1000 consecutively measured electrical conductance traces without any data selection. For each molecule, the recorded electrical conductance in a single trace starts from the point where the electrical conductance value is saturated, i.e., the upper bound of the measurement range, and extends to the point where the co electrical conductance reached the noise floor when the tip is fully retracted from the substrate.

Seebeck coefficient measurements

Seebeck coefficient measurements of single molecule junctions were implemented by a modified STM-BJ method. A resistive heater attached to the sample holder was employed to heat the sample to various temperatures. To measure the thermopower of a molecular junction, the tip motion was modified from the approach described above for electrical conductance measurements. Specifically, the tip withdrawal was stopped immediately, once the most probable electrical conductance value, determined from the electrical conductance measurements, was reached. While the molecule was held between the tip and the substrate, the bias applied to the tip was repeatedly switched between 100 mV and 0 mV with a duty cycle of 0.4 s until the junction ruptured. Currents flowing through the junction under applied biases of 100 mV and 0 mV (with the substrate grounded and the bias applied to the tip, see Figure S29 for sample data obtained for a **Zn-P2** junction under a temperature differential of 11.4 K) were recorded in real time and were used to determine the electrical conductance and thermoelectric current, respectively.

The short circuit thermoelectric current (I_{th}) flowing through the molecular junction, i.e. the electrical current induced by the applied temperature differential ($\Delta T = T_{substrate} - T_{tip}$) under a zero applied voltage bias, and the electrical conductance (G), measured in the same switching cycle, were used to estimate the open circuit thermoelectric voltage ($\Delta V_{TE} = V_{substrate} - V_{tip}$). As shown in the Figure S30, we define I_{th} to be positive when the short circuit thermoelectric current flows through the junction from the tip to the substrate. Based on this definition, we relate the expected open circuit thermoelectric voltage (ΔV_{TE}), to the measured short circuit thermoelectric current (I_{th}) and the electrical conductance (G) via: $\Delta V_{TE} = I_{th}/G$. The corresponding histograms of the thermoelectric voltage were first constructed using data obtained from over 5000 switching cycles (e.g., see Fig. 6 of the manuscript for histograms), and the peak value of the histogram was extracted by fitting a Gaussian distribution to the histogram and identifying the peak of the Gaussian distribution curve. Next, to obtain the Seebeck coefficient (i.e., thermopower) of the molecular junction we applied a linear fit to the measured values of ΔV_{TE} (obtained from the peaks of histograms corresponding to various temperature differentials ΔT) and determined the Seebeck coefficient of the molecular junction ($S_{junction}$) using:

$$S_{junction} = -\frac{\Delta V_{TE}}{\Delta T} + S_{Cu} = -\frac{I_{th}}{G\Delta T} + S_{Cu}$$

Here, we include the Seebeck coefficient of copper (1.94 μV/K) in the above equation as a copper wire is involved in our measurements as shown schematically in Figure S30.

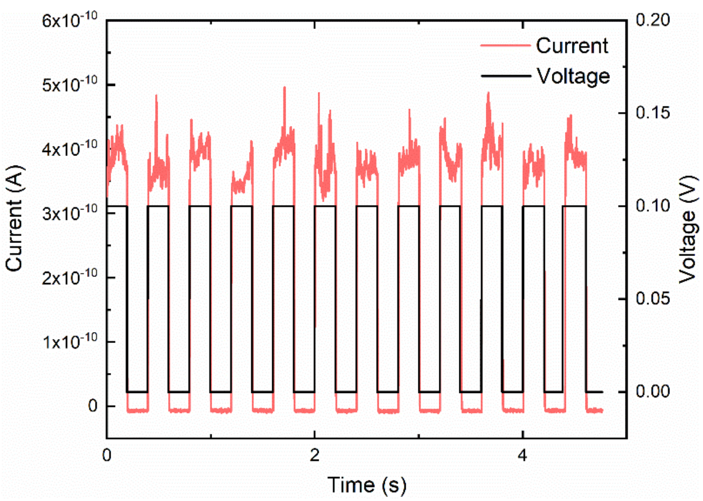

Figure S29. Applied voltage bias and the measured thermoelectric current in an experiment where a **Zn-P1** molecular junction was studied under an applied temperature differential of 11.4 K. The voltage was switched from 0.1 V to 0 V with a periodicity of 0.4 s, and the resulting current was recorded simultaneously. The current measured under an applied bias of 0.1 V yields the electrical conductance of the junction (here ~$5\times10^{-5}G_0$), while the current when the applied bias was 0 V represents the thermoelectric current (I_{th}) and is related to the thermoelectric voltage, as described in the text.

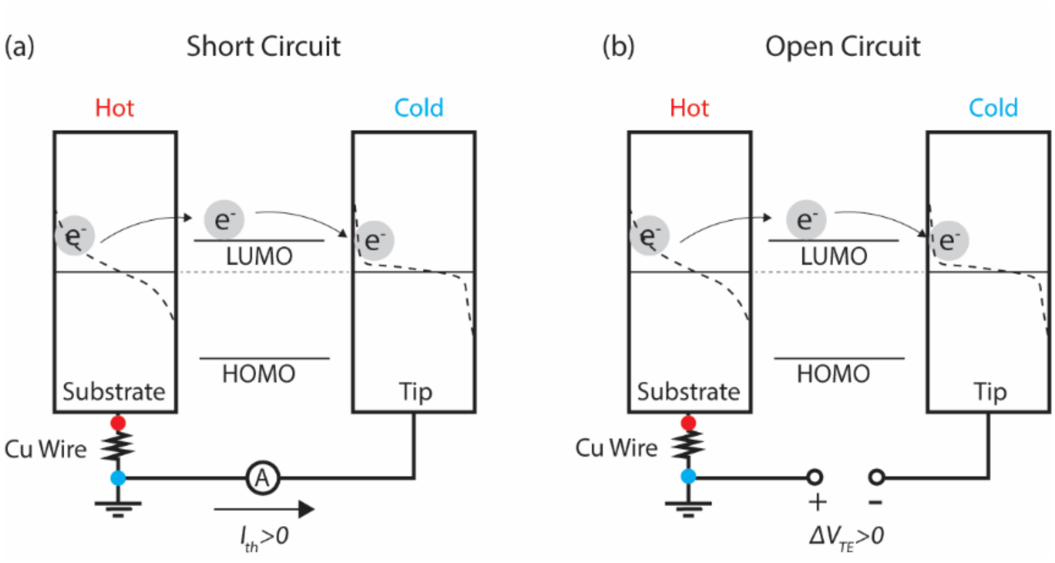

Figure S30. Convention for the sign of short circuit thermoelectric current (I_{th}) adopted in this work. (a) I_{th} is defined to be positive when the current flows through the molecular junction from the tip to the substrate. (b) Expected relative voltage of the hot and cold electrodes when a LUMO dominated molecular junction bridges them, resulting in the hot electrode being at a positive voltage with respect to the cold electrode. The expected open circuit voltage (ΔV_{TE}) can be related to the short circuit current via $\Delta V_{TE} = I_{th}/G$. The red and blue dots on the copper wire indicate the hot and cold ends of the wire, respectively.

Estimating the temperature differential applied across the molecular junction

Since experiments were performed under ambient conditions with electrochemically etched tips, it is important to carefully estimate the temperature differential applied across the molecular junction. The temperature differential maintained between the Au tip and the sample substrate as a function of heating current was measured by attaching K-type thermocouples (ready-made insulated thermocouples with Kapton®, PFA, glass braid insulation and molded connectors) to the tip and substrate simultaneously. Since the measurements were performed in an ambient environment, the tip geometry and effects of the water meniscus formed between the tip and substrate, which affects the actual temperature differential across the substrate-tip gap, have to be estimated. We accomplished this by performing simulations using the finite element method (FEM) implemented in COMSOL Multiphysics 5.5. We first characterized the exact geometry of our tips by imaging them in a scanning electron microscope (SEM) at different magnifications to identify the geometry of typical electrochemically etched Au tips (see Figure S31). Tips formed in the electrochemical etching process were found to have consistent opening angles between 25 to 27 degrees and a 1 μm-diameter ball-shaped distal end.

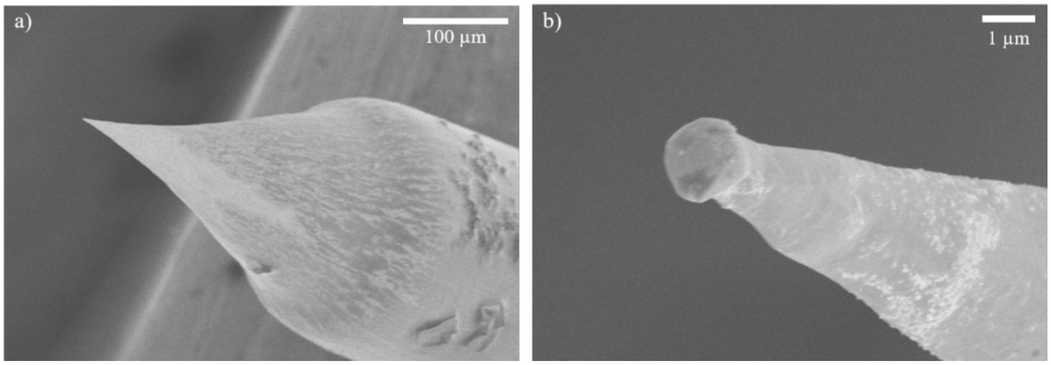

Figure S31. SEM images of electrochemically etched Au tips. It can be seen that the opening angle is around 25° and the diameter of the Au wire is around 230 μm. The distal tip of the etched Au wire is ball-shaped and has a diameter of around 1 μm.

Therefore we considered both the effects of tip geometry (i.e. the ball-shaped tip, see the SEM images) and ambient humidity, which introduces a water meniscus between the tip and the substrate (as described in previous work[S13-S15]) and results in an increased tip temperature due to thermal conduction through the meniscus. In our modelling, the thermal conductivity of air and water were chosen to be 0.01 W/m K[S13,S14] and 0.6 W/m K[S15], respectively. The tip-substrate distance was set to 2 nm and the temperatures of the heated sample and the base of the tip were chosen as 333 K and 293 K, respectively. Further, based on the well-established meniscus analysis in the tip-substrate model from past work[S13,S14], we estimate the radius of the water meniscus R_w to be ~50 nm.

Using these parameters, we simulated the temperature profile along the substrate-molecule-tip junction. The simulated results (Figure S32a) indicate that the temperature at the tip end is 314 K. Therefore, the

actual temperature differential across the tip and substrate is 19 K, which is lower than the nominally applied temperature differential of 40 K. To further understand the effects of the size of the water meniscus[S13,S14] on the simulation results, we varied R_w from 30 nm to 80 nm to determine the uncertainties stemming from the water meniscus geometry. The corresponding temperature distributions are shown in Figure S32b, which provides an estimate for the uncertainties of the temperature differential (± 7.5%) while determining the Seebeck coefficient. From this analysis we conclude that for our measurement configuration the true temperature differentials across the molecular junction are lower than what was estimated by measuring the tip and sample temperatures using thermocouples. Since the heat transfer equations are all linear we expect from this finite element analysis that the temperature differentials across a molecular junction are ~47.5% of the applied temperature differentials at all substrate temperatures. The validity of this analysis was confirmed by performing control experiments on molecular junctions with known Seebeck coefficients, as described below.

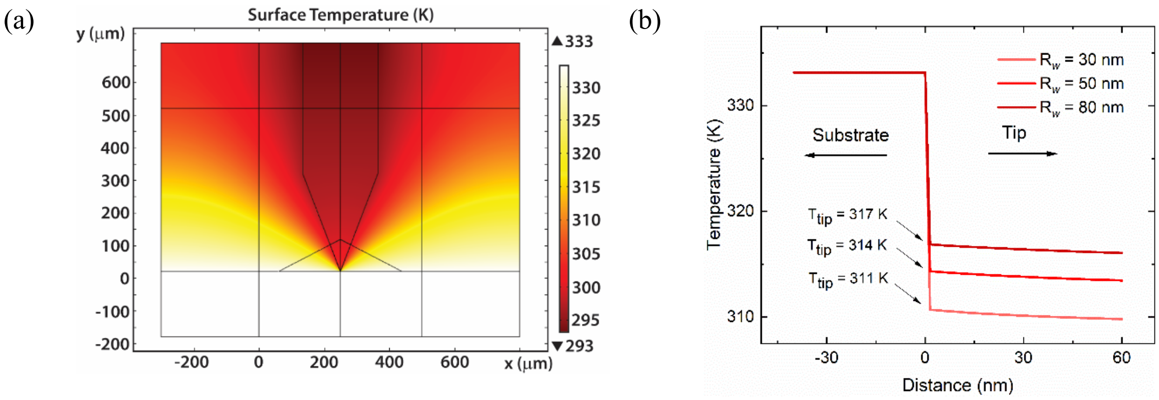

Figure S32. COMSOL simulation results. (a) Simulated temperature field when the radius of the water meniscus is set to 50 nm. (b) Substrate-tip temperature distribution along the axis of symmetry when the radius of the water meniscus is varied from 30 nm to 80 nm.

Control experiments with various molecular junctions

In order to demonstrate the validity of our measurement schemes and temperature estimation approach, we performed control experiments on various molecular junctions, where conduction is dominated by either holes (Au-*benzenedithiol*-Au), i.e. a HOMO-dominated junction, or electrons (Au-*bipyridine*-Au and Au-*fullerene*-Au), i.e. LUMO-dominated junctions, and applied the measurement schemes and analysis approaches described above. The results from the control measurements on Au-*benzenedithiol*-Au are shown in Figure S33 and the Seebeck coefficient is measured to be 12.20 ± 2.17 μV/K, which agrees well with the formerly reported values[S16,S17]. As is shown in Figure S34, Au-*bipyridine*-Au and Au-*fullerene(C_{60})*-Au junctions have negative Seebeck coefficients with values of -7.11 ± 1.66 μV/K and -13.67 ± 2.58 μV/K, respectively, which also agree well with the previous measurements.[S18,S19]

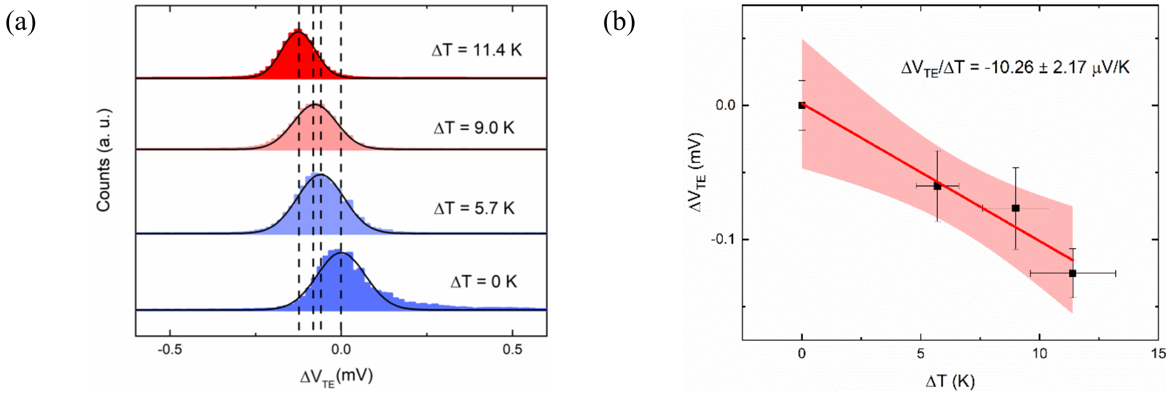

Figure S33. Control experiment using Au-*benzendithiol*-Au junctions. (a) Histograms of thermoelectric voltages at various temperature gradients Δ*T* and the corresponding Gaussian fits to the peak positions. (b) Thermoelectric voltage of Au-benzenedithiol-Au as a function of Δ*T*. Linear fitting gives the slope of -10.26 ± 2.17 μV/K, which corresponds to a Seebeck coefficient of 12.20 ± 2.17 μV/K after compensating for the Seebeck coefficient of Cu wires.

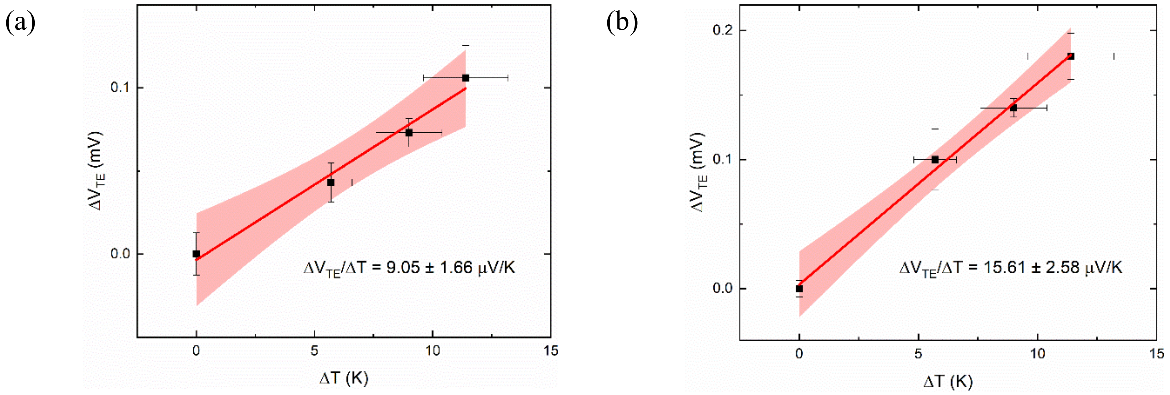

Figure S34. Temperature-dependent thermoelectric voltage plots of control experiments using (a) Au-*bipyridine*-Au and (b) Au-*fullerene*-Au junctions. Linear fittings give the slopes of 9.05 ± 1.66 μV/K and 15.61 ± 2.58 μV/K, which correspond to Seebeck coefficients of -7.11 ± 1.66 μV/K and -13.67 ± 2.58 μV/K for Au-*bipyridine*-Au and Au-*fullerene*-Au junctions, respectively.

Part 6. Transport calculations using DFT

As described in the main paper, see Figures 7 and 8, we studied the four porphyrin molecules **P1**, **P2**, **Zn-P1** and **Zn-P2** both in top-top (TT) and hollow-hollow (HH) contact geometries. In the TT junctions, the molecule is attached to pyramids of 20 gold atoms on each side, which are arranged in four planes. The sulfur atoms of the SMe anchors bind to the gold atoms of the tips. The two outermost planes on each side of this Au-molecule-Au extended central cluster (ECC) are later regarded as part of the semi-infinitely extended, crystalline electrodes in the transport calculations. In the HH junctions, the tip atoms of the gold pyramids are removed. However, the sulfur atoms of the SMe anchors still bind preferably to only one of the three gold atoms of the blunt tips due to lone-pair bonding.

Since the two outermost gold layers on each side are considered as crystalline bulk, we kept them fixed during the geometry optimizations. The basis set was chosen to be def-SV(P)[S20] in all cases and the exchange-correlation functional was PBE[S8]. The DFT total energy was converged to “scfconv 7”, the geometry relaxation to “gcart 3”.

The transmission curves based on the DFT quantum transport calculations are shown in Figure S35 for the TT and HH junctions. Resulting electrical conductance and thermopower values are listed in Table S6.

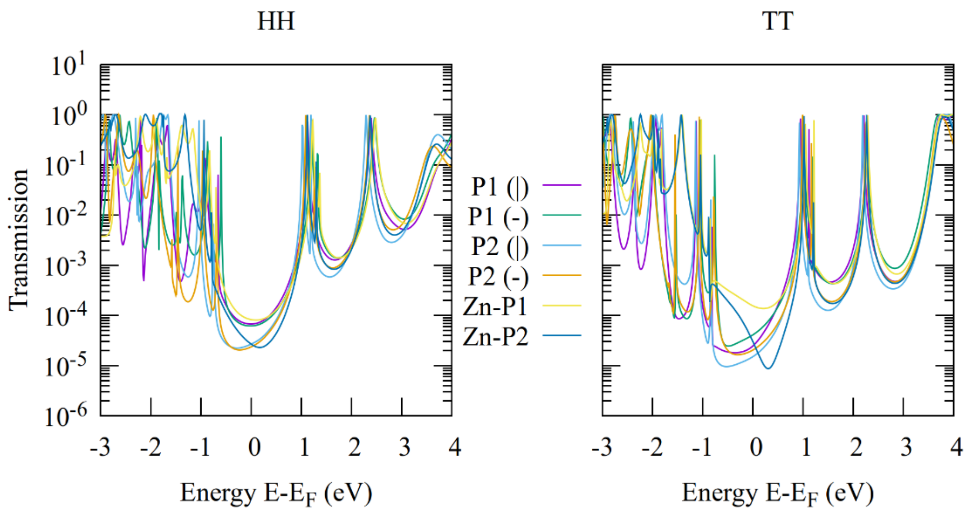

Figure S35. Transmission curves for single-molecule junctions containing **P1**, **P2**, **Zn-P1** and **Zn-P2** in TT and HH configurations using DFT.

Table S6. Calculated values for the electrical conductance *G* and thermopower *S* of single-molecule junctions containing **P1**, **P2**, **Zn-P1** and **Zn-P2** using DFT.

	**HH**		**TT**	
**Molecule**	G (G_0)	S (μV/K)	G (G_0)	S (μV/K)
**P1 (|)**	6.80×10^{-5}	-0.19	6.80×10^{-5}	-15.60
**P1 (-)**	6.22×10^{-5}	-0.44	4.13×10^{-5}	-11.05
**P2 (|)**	2.69×10^{-5}	-7.90	1.52×10^{-5}	-11.02
**P2 (-)**	2.38×10^{-5}	-7.86	2.06×10^{-5}	-9.91
**Zn-P1**	8.31×10^{-5}	3.36	1.57×10^{-4}	8.51
**Zn-P2**	2.71×10^{-5}	11.76	3.09×10^{-5}	36.33

Part 7. Transport calculations using DFT+Σ

Transport calculations with the DFT+Σ correction scheme were performed, as described in the literature and in the main text.[S21,S22] Junction geometries are those determined with DFT. To compute the shifts Δ_{occ} and Δ_{vrt}, determining are so-called image charge corrections, we consider the charge distribution of molecular orbitals and assume perfectly flat metal planes to be positioned 1.47 Å in front of the first unrelaxed layer, when seen from the molecule. Detailed transport results with the DFT+Σ method are presented in Figure S36 and Table S7.

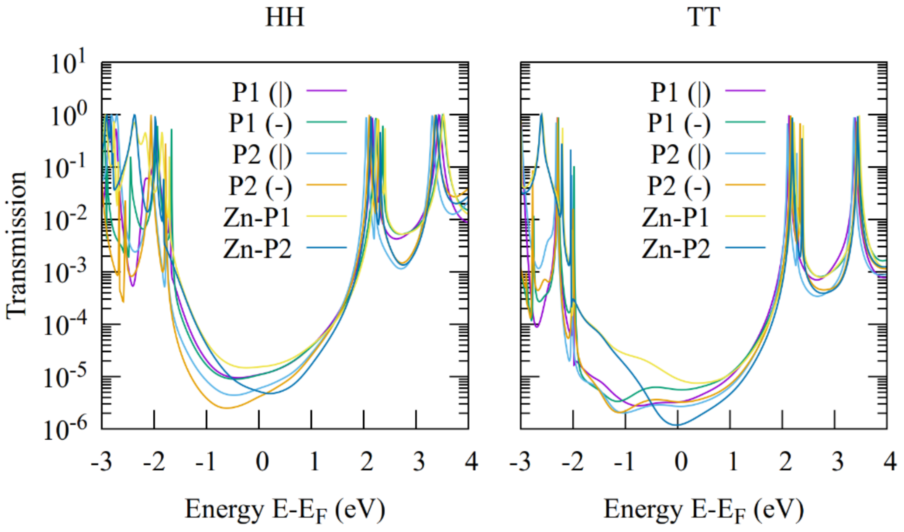

Figure S36. Transmission curves for single-molecule junctions containing **P1**, **P2**, **Zn-P1** and **Zn-P2** in HH and TT geometries using DFT+Σ.

Table S7. Calculated values for the electrical conductance *G* and thermopower *S* of single-molecule junctions containing **P1**, **P2**, **Zn-P1** and **Zn-P2** using DFT+Σ.

	HH		TT	
**Molecule**	***G*** **(*G*$_0$)**	***S*** **(µV/K)**	***G*** **(*G*$_0$)**	***S*** **(µV/K)**
**P1 (|)**	1.08×10^{-5}	-3.56	3.23×10^{-5}	-1.61
**P1 (-)**	1.07×10^{-5}	-4.14	5.63×10^{-6}	1.00
**P2 (|)**	5.89×10^{-6}	-6.01	2.70×10^{-6}	0.69
**P2 (-)**	4.10×10^{-6}	-8.21	3.27×10^{-6}	1.10
**Zn-P1**	1.52×10^{-5}	-1.60	9.00×10^{-6}	7.22
**Zn-P2**	5.19×10^{-6}	5.03	1.19×10^{-6}	-1.06

Part 8. Transport calculations using DFT+Σ-IS

Electronic transport within the method DFT+Σ-IS is computed as described in the main text. Image charge corrections are evaluated for each molecular orbital separately, and this procedure, applied to orbital *i* now, is identical to that used for HOMO and LUMO orbitals in the DFT+Σ scheme. The energy of the molecular orbital *i* is furthermore corrected through G_0W_0 calculations, which yield $\epsilon_{\mathrm{GW}}^{i} - \epsilon_{\mathrm{DFT}}^{i}$. As these latter corrections are computed for molecular orbitals of the isolated molecule but shall later be applied to energy levels in the molecular subspace of the junction, both states need to be related. Detailed transport results with the DFT+Σ-IS method are presented in Figures S37 to S41.

As briefly described in the main text, the mapping between an orbital $\tilde{\psi}_i$ of the molecular subspace in the junction and a molecular orbital ψ_j of the isolated molecule is crucial and can be characterized by the scalar product between both states. The evaluation of the scalar product uses the expansion of both wave functions

$$\psi_i(\mathbf{r}) = \sum_{\mu} C_{i,\mu}\, \phi_\mu(\mathbf{r})$$

and

$$\tilde{\psi}_j(\mathbf{r}) = \sum_{\mu} \tilde{C}_{j,\mu}\, \phi_\mu(\mathbf{r})$$

in terms of atom-centered basis functions ϕ_μ. It is thus given by

$$M_{i,j} = \left(\psi_i, \tilde{\psi}_j\right) = \sum_{\mu,\nu} C_{i,\mu}^{*} S_{\mu,\nu} \tilde{C}_{j,\nu}$$

with the scalar product matrix *M* and the elements of the overlap matrix

$$S_{\mu,\nu} = \int \mathrm{d}^3 r \phi_\mu^{*}(\mathbf{r}) \phi_\nu(\mathbf{r}).$$

The scalar product matrix *M* is thus given by the matrix product

$$M = C\, S\, \tilde{C}^T$$

of the two coefficient matrices C and $\tilde{C}$ with the overlap matrix *S*.

If the sets of eigenstates $\tilde{\psi}_i$ and ψ_j were identical, the scalar product of a group of orthonormal molecular orbitals would be calculated, and *M* would thus be the identity matrix. For the mapping an absolute value of 1 of a matrix element $M_{i,j}$ hence indicates perfect agreement between the *i*th eigenstate of the molecular subspace of the junction and the *j*th molecular orbital of the isolated molecule. A smaller absolute value of the matrix element $M_{i,j}$ indicates a worse agreement, until both states are orthogonal for an absolute value of 0. Indeed two states with an equally good agreement may exist, if $M_{i,j} < 2^{-1/2} \approx 0.71$.

Thus a matching threshold γ for identifying eigenstates should be located between around 0.7 and 1. If the matching threshold were chosen too low, i.e. below 0.7, some orbitals might be shifted in the wrong way due to a wrong identification. If the matching threshold were chosen too big, i.e. close to 1, the advantage of shifting orbitals individually instead of using one default shift for occupied or unoccupied states as used in DFT+Σ would be lost. As discussed in the paper, we choose a matching threshold γ=0.9, above which a mapping is identified to be sufficiently good.

To understand the influence of the matching threshold, we computed the electronic transport quantities G and S for all junction types as a function of γ. This study is shown in Figure S42. Based on the evolution of G and S as a function of γ in the interval from 0.7 to 1, γ=0.9 yields sufficiently robust results not only with regard to an agreement with experimental values, but also with regard to the stability for smaller changes of the matching threshold.

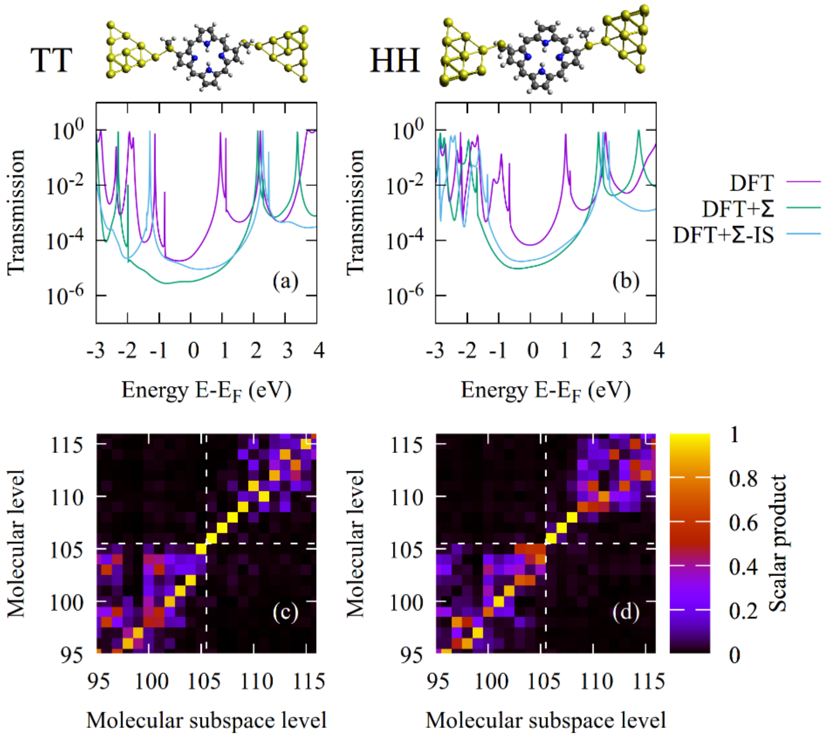

Figure S37. (a,b) Transmission curves for Au-**P1(l)**-Au junctions with the DFT+Σ-IS method, using individual shifts that are accepted according to a mapping analysis. As a comparison the DFT and DFT+Σ transmission curves are shown as well. Junction geometries are displayed at the top of the figure. (c,d) Mapping between the eigenstates of the molecular subspace in the corresponding single-molecule junction and the molecular orbitals of the isolated molecule **P1(l)** in TT and HH geometries around the HOMO (index 105) and LUMO (index 106), separated by the dashed white line. For TT, the absolute value of the scalar product is above the matching threshold for HOMO-3 to LUMO+5. For HH, this requirement is fulfilled for LUMO to LUMO+2. Although the matching condition is not fulfilled for the HOMO level in HH, we still correct its energy with the best match to ensure the gap opening effect.

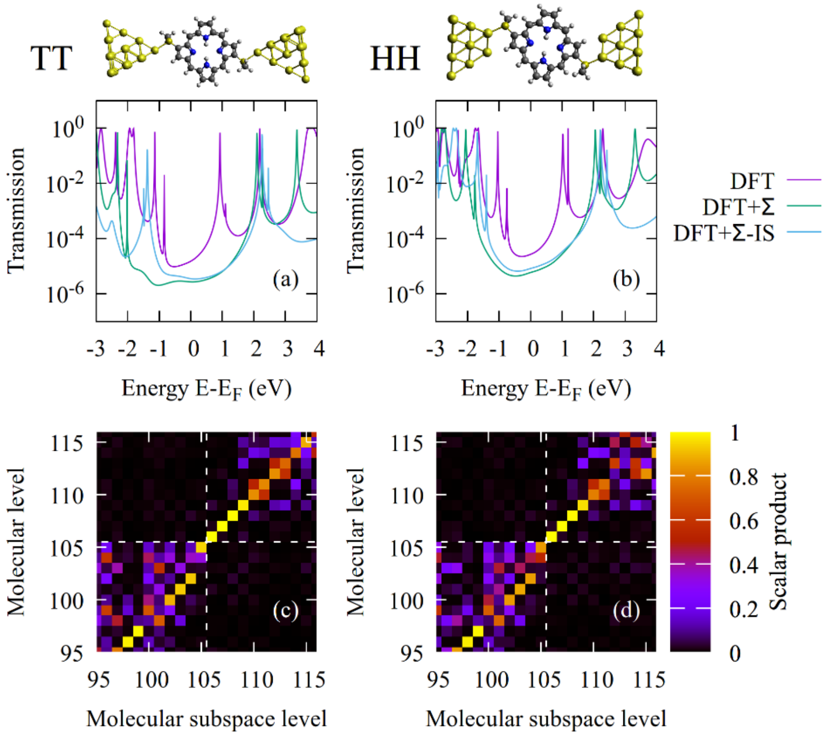

Figure S38. Same as Figure S36 but for the molecule **P2(|)**. For TT, the absolute value of the scalar product is above the matching threshold for HOMO-2 to LUMO+3. For HH, this requirement is fulfilled for LUMO to LUMO+3. Although the matching condition is not fulfilled for the HOMO level in HH, we still correct its energy with the best match to ensure the gap opening effect.

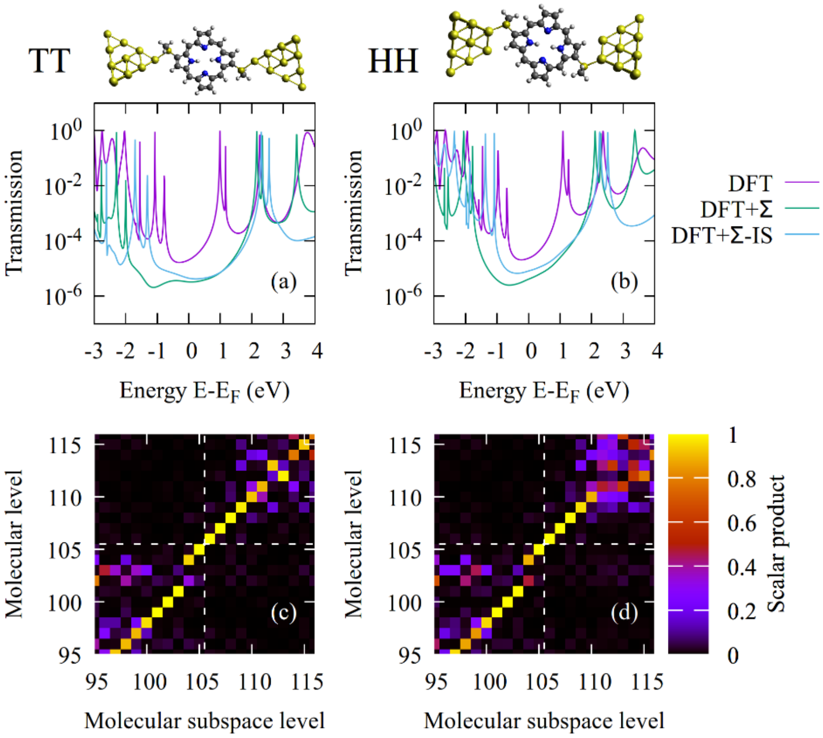

Figure S39. Same as Figure S36 but for the molecule **P2(-)**. For TT, the absolute value of the scalar product is above the matching threshold for HOMO-5 to LUMO+4. For HH, this requirement is fulfilled for HOMO to LUMO+3.

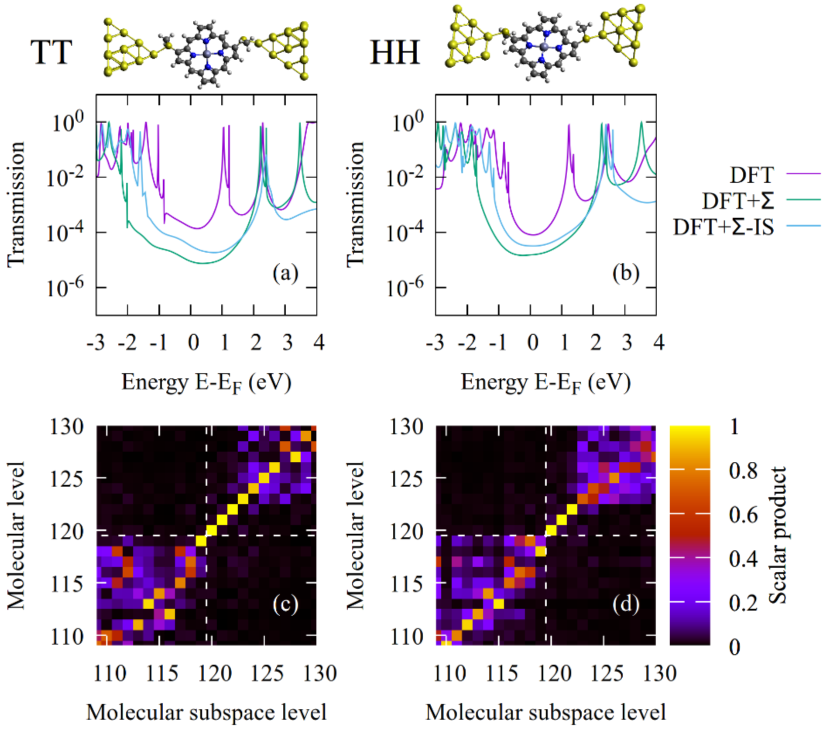

Figure S40. (a,b) Transmission curves for Au-**Zn-P1**-Au junctions with the DFT+Σ-IS method, using individual shifts that are accepted according to a mapping analysis. As a comparison the DFT and DFT+Σ transmission curves are shown as well. Junction geometries are displayed at the top of the figure. (c,d) Mapping between the eigenstates of the molecular subspace in the corresponding single-molecule junction and the molecular orbitals of the isolated molecule **Zn-P1** in TT and HH geometries around the HOMO (index 119) and LUMO (index 120), separated by the dashed white line. For TT, the absolute value of the scalar product is above the matching threshold for HOMO to LUMO+6. For HH, this requirement is fulfilled for HOMO to LUMO+2.

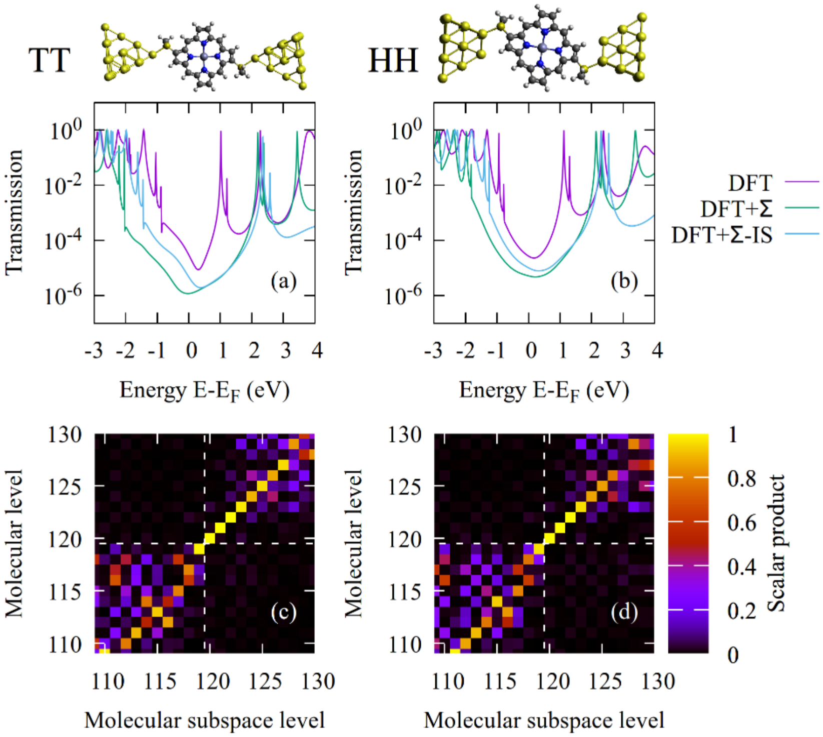

Figure S41. Same as Figure S39 but for the molecule **Zn-P2**. For TT, the absolute value of the scalar product is above the matching threshold for HOMO to LUMO+3. For HH, this requirement is fulfilled for HOMO to LUMO+3.

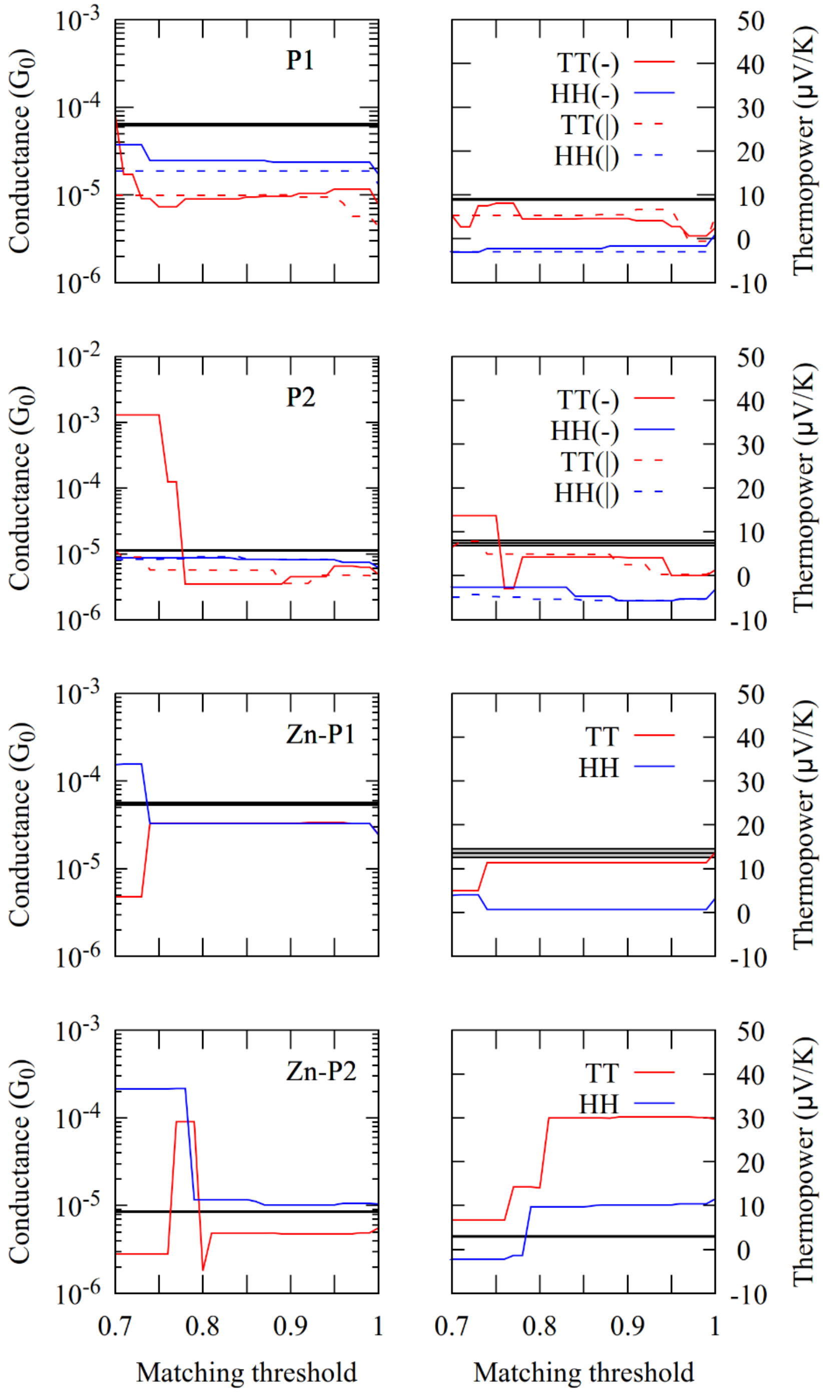

Figure S42. Dependence of electrical conductance and thermopower of porphyrin junctions on the assumed matching threshold γ for energy corrections of individual molecular orbitals in the DFT+Σ-IS method. The horizontal black line in the middle indicates the measured value, and grey boxes bounded by black lines show the experimental uncertainty.

Part 9. Quantum interference in Zn-porphyrin molecules

In Ref. S23 a two-level model was presented, where both levels are on the same side of the Fermi energy E_F. The levels had opposite parity and were coupled equally well to left (L) and right (R) electrodes, so $\Gamma_1=\Gamma_{1L}=\Gamma_{1R}$ and $\Gamma_2=\Gamma_{2L}=\Gamma_{2R}=a^2\Gamma_1$, with $a^2=\Gamma_2/\Gamma_1$ being the ratio between the couplings of the first level Γ_1 and the second level Γ_2. The transmission function of this two-level model has the form

$$\tau(E) = \Gamma_1^2 \left| \frac{1}{E - E_1 + i\,\Gamma_1} - \frac{a^2}{E - E_2 + i\,\Gamma_1} \right|^2 .$$

All energies in the expression are measured with respect to the Fermi energy $E_F=0$. The authors discuss that the described two-level model can show a destructive quantum interference behavior with a vanishing transmission at the Fermi energy, if $E_2=a^2E_1$. Furthermore, they propose a Zn-porphyrin molecule as a possible realization of their model, because the HOMO and HOMO-1 are nearly degenerate and show opposite parity on the carbon framework, if coupled as in our **Zn-P1** molecule. This work inspired us, to compare here the **P1** and **Zn-P1** molecules with **P2** and **Zn-P2**.

The isolated Zn-porphyrin molecule without any anchoring groups, called **Zn-P0** in Section 4, possesses D_{4h} point group symmetry. We performed symmetry-adapted DFT calculations with TURBOMOLE[S6], using the def-SV(P) basis set[S20], the exchange-correlation functional PBE[S8] and a convergence in total energy to better than 10^{-8} Hartree ("scfconv 8"). For the HOMO level we find the irreducible representation A_{2u} and an energy of -4.95 eV with respect to the vacuum level, for the HOMO-1 the representation is A_{1u} and the energy is -5.09 eV. The shapes of the two orbitals are shown in Figure S43 and agree with those presented in Ref. S23. For completeness, we also show the HOMO-2, a level with the representation B_{1g} at -5.49 eV, and the LUMO, which is degenerate with the representation E_g at the energy -2.90 eV. Thus, the HOMO and HOMO-1 of the isolated Zn-porphyrin molecule can be seen as a two-level model with the required parities to demonstrate the behavior discussed in Ref. S23.

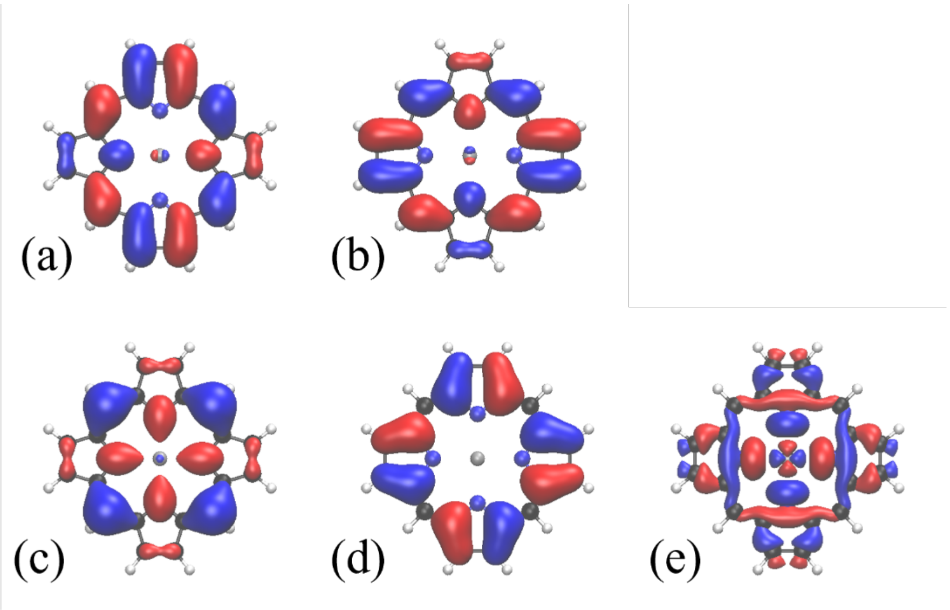

Figure S43. Molecular orbitals of the isolated Zn-porphyrin molecule, **Zn-P0**. (a,b) Degenerate LUMO states, (c) HOMO, (d) HOMO-1, and (e) HOMO-2.

To understand, why we do not see the expected high difference in the electrical conductance and in the thermopower between the molecules **Zn-P1** and **Zn-P2**, we inspect the orbitals of the junctions. In the extended central cluster (ECC), consisting of the molecule coupled to gold pyramids of 19 (HH) or 20 (TT) atoms on both sides, the presence of anchoring groups as well as of the gold electrodes might influence the appearance and energies of the relevant orbitals. We performed a Mulliken population analysis of the orbitals of the ECC to determine the weight of each orbital on the molecule, which can vary from 0 to 2[S24]. In Figure S44, this quantity is plotted as vertical lines together with the DFT transmission curves for the **Zn-P1** and **Zn-P2** molecules, both in the TT junction geometry. A peak in the transmission curve typically corresponds to an orbital with a high population on the molecule. Transport is clearly connected to the occupied frontier orbitals in the vicinity of the Fermi energy E_F. Within a range of -1.5 eV below E_F, we find for both junctions three orbitals with a population on the molecule that is bigger than 1. These orbitals are plotted in Figure S45.

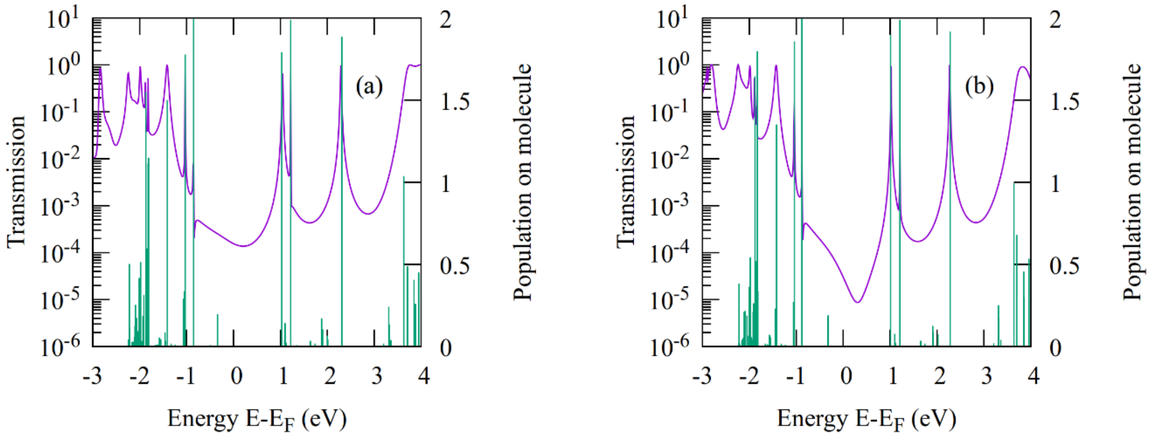

Figure S44. DFT transmission curves (violet, left axis) of (a) **Zn-P1** (TT) and (b) **Zn-P2** (TT) together with the Mulliken population of the ECC orbitals on the molecule (green, right axis).

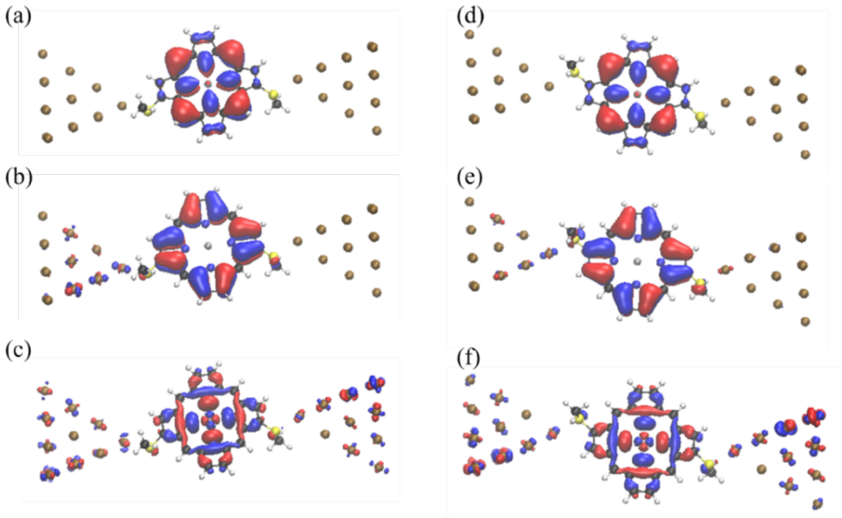

Figure S45. Three highest occupied ECC orbitals with a population on the molecule that surmounts 1 for (a)-(c) **Zn-P1** (TT) and (d)-(f) **Zn-P2** (TT).

At the energy E-E_F=-0.85 eV for **Zn-P1** and E-E_F=-0.88 eV for **Zn-P2** we find orbitals with a population of 2 on the molecule. This means that the states are fully localized on the molecules and not extended into the gold electrodes. The localized states cause Fano resonances in the corresponding transmission curves. These orbitals are shown in Figure S45a and S45d. The other two orbitals, presented in Figure S45, are located at energies E-E_F of -1.03 eV and -1.41 eV for **Zn-P1** and -1.04 eV and -1.42 eV for **Zn-P2,** respectively. With a population between 1.35 and 1.86 on the molecule, this group of orbitals is well localized on the molecule but still extends somewhat into the gold electrodes und thus leads to regular transmission resonances. The small peaks in the population plot inside the HOMO-LUMO gap that are found for both junctions, originate from orbitals that are mainly located on the gold, but still have a small population on the molecule, mostly on the SMe anchoring groups.

If we compare the shape of the orbitals of the ECC in Figure S45 with the orbitals of the isolated Zn-porphyrin molecule in Figure S43, we can identify the HOMO of the isolated molecule with the orbitals responsible for the Fano peaks. The two orbitals responsible for the regular transmission peaks are instead based on the HOMO-1 and HOMO-2 of the isolated molecule.

We conclude that the molecules **Zn-P1** and **Zn-P2**, similarly to **P1** and **P2,** turn out to be no good realization of the two-level model proposed in Ref. S23. The HOMO level of the isolated molecule turns out to be a localized state, when coupled to the electrodes in the junction geometries assumed here, contributing as a Fano resonance to the transmission. More energy levels than just the HOMO and HOMO-1 play a role for transport, such as the HOMO-2, and their contribution to the behavior of the electrical conductance and thermopower of the molecules in the junction cannot be neglected.

Part 10. Transmission eigenchannels

Within the Landauer-Büttiker formalism we express the transmission through a molecular junction as

$$\tau(E) = \mathrm{Tr}[\Gamma_\mathrm{L}(E) G_\mathrm{C}^\mathrm{r}(E) \Gamma_\mathrm{R}(E) G_\mathrm{C}^\mathrm{a}(E)] = \sum_i \tau_i(E).$$

The total transmission τ can be decomposed into contributions τ_i from individual transmission eigenchannels. The wavefunctions of the transmission eigenchannels are determined under the boundary condition of a wave that enters e.g. from the left electrode, as discussed in Refs. S25, S26.

In Figure S46 the total transmission of the molecule **P1(-)** in the HH geometry is plotted together with the transmissions of the three most transparent eigenchannels. We compare the three methods DFT, DFT+Σ and DFT+Σ-IS in the different panels and observe that the transmission is determined in all cases by a single eigenchannel. Importantly, the shape of this eigenchannel, evaluated at the Fermi energy, is basically not affected by the method that is used to compute the transmission.

In Figure S47, using DFT, a similar comparison between the transmission eigenchannels of the molecules **P2(-)** and **P2(|)** is shown for both the TT and HH geometries. Like for the **P1(-)** molecule in the HH geometry, the transmission at the Fermi energy is dominated by one transmission eigenchannel. The shape of the transmission eigenchannel depends on the junction geometry but only slightly on the arrangement of the inner hydrogen atoms.

We find a prevalent transmission eigenchannel for most of the junctions. In junctions containing the molecule **Zn-P1**, see Figure S48(a), the transmissions of the first and second eigenchannels can be of comparable size at some energies inside the HOMO-LUMO gap. However, at the Fermi energy, the transmission is dominated by a single eigenchannel in all contact geometries that we studied, i.e. HH and TT. Only for junctions based on the molecule **Zn-P2** we find a change of the character of the leading two transmission eigenchannels close to the Fermi energy, see Figure S48(b).

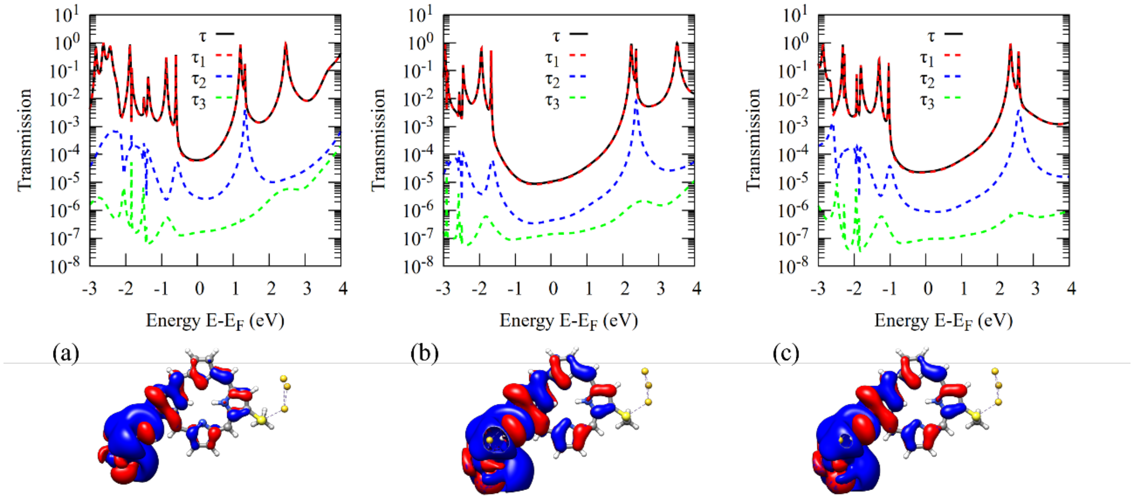

Figure S46. Total transmission τ and transmissions τ_i of the three highest contributing eigenchannels together with the wavefunction of the most transmissive eigenchannel, evaluated at E_F for a wave entering from the left electrode, for **P1(-)** in the HH junction geometry. The three panels show the results for the different methods: (a) DFT, (b) DFT+Σ and (c) DFT+Σ-IS.

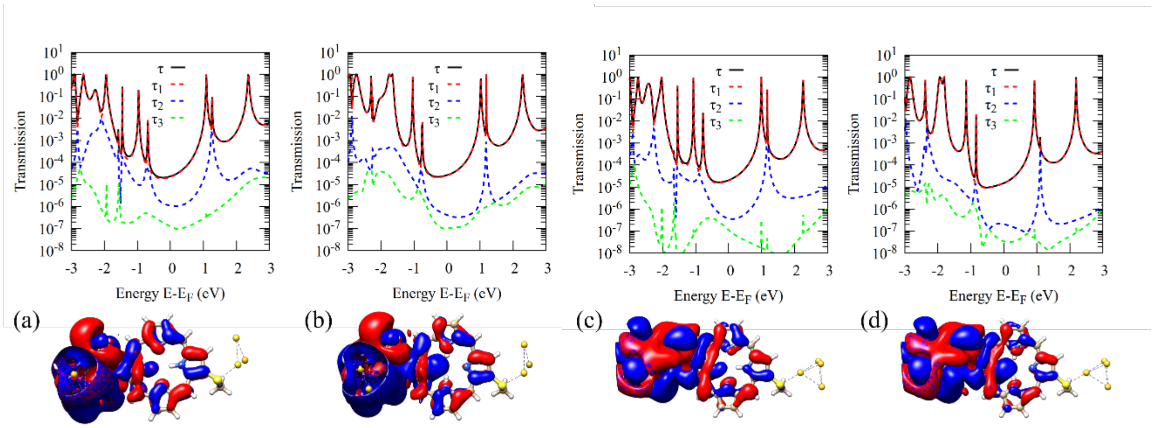

Figure S47. Same as Figure S45 but for (a) **P2(-)** in HH, (b) **P2(|)** in HH, (c) **P2(-)** in TT, (d) **P2(|)** in TT geometries within DFT. In all cases the total transmission τ inside the HOMO-LUMO gap is clearly determined by the first eigenchannel. The shape of this eigenchannel, shown at the bottom, depends on the binding geometry but only slightly on the arrangement of inner H atoms.

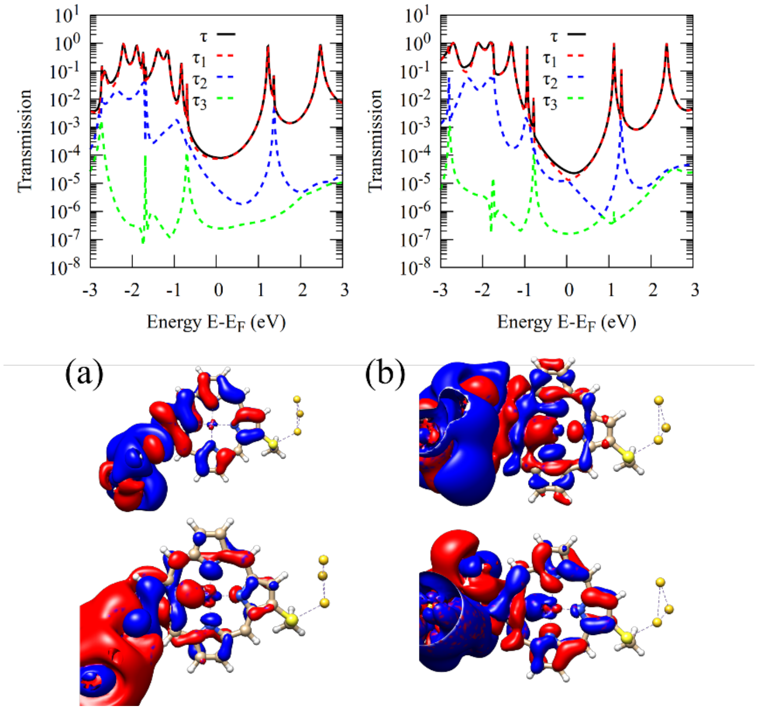

Figure S48.Total transmission τ and the transmissions τ_i of the three highest contributing eigenchannels for (a) **Zn-P1** in HH and (b) **Zn-P2** in HH junctions within DFT. The wavefunctions of the two most transmissive eigenchannels are shown at the bottom, evaluated at E_F for a wave entering from the left electrode.

References

1. Kotoku, N.; Fujioka, S.; Nakata, C.; Yamada, M.; Sumii, Y.; Kawachi, T.; Arai, M.; Kobayashi, M. *Tetrahedron* **2011**, *67*, 6673-6678.
2. Law, K. R.; McErlean, C. S. P. *Chem. Eur. J.* **2013**, *19*, 15852-15855.
3. Sugiura, K.; Ushiroda, K.; Johnson, M. T.; Miller, J. S.; Sakata, Y. *J. Mater. Chem*. **2000**, *10*, 2507-2514.
4. Muchowski, J. M.; Hess, P. *Tetrahedron Lett*. **1988**, *29*, 3215-3218.
5. Wurst, J. M.; Verano, A. L.; Tan, D. S. *Org. Lett.* **2012**, *14*, 4442-4445.
6. Balasubramani, S. G.; Chen, G.P.; Coriani, S.; Diedenhofen, M.; Frank, M. S.; Franzke, Y. J.; Furche, F.; Grotjahn, R.; Harding, M. E.; Hättig, C.; Hellweg, A.; Helmich-Paris, B.; Holzer, C.; Huniar, U.; Kaupp, M.; Marefat Khah, A.; Karbalaei Khani, S.; Müller, T.; Mack, F.; Nguyen, B. D.; Parker, S. M.; Perlt, E.; Rappoport, D.; Reiter, K.; Roy, S.; Rückert, M.; Schmitz, G.; Sierka, M.; Tapavizca, E.; Tew, D. P.; van Wüllen, C.; Voora, V. K.; Weigend, F.; Wodyński, A.; Yu, J. M. *J. Chem. Phys.* **2020**, *152*, 184107.
7. Schäfer, A.; Huber, C.; Ahlrichs, R. *J. Chem. Phys.* **1994**, *100*, 5829-5835.
8. Perdew, J. P.; Burke, K.; Ernzerhof, M. *Phys. Rev. Lett.* **1996**, *77*, 3865-3868.
9. Martin, R. Electronic Structure: Basic Theory and Practical Methods.Cambridge University Press, 2020.
10. Kaplan, F.; Harding, M. E.; Seiler, C.; Weigend, F.; Evers, F.; van Setten, M. J. *J. Chem. Theory Comput.* **2016**, *12*, 2528-2541.
11. Van Setten, M. J.; Weigend, F.; Evers, F. *J. Chem. Theory Comput.* **2013**, *9*, 232-246.
12. Hedin, L. *Phys. Rev.* **1965**, *139*, A796-A823.
13. Luo, K. *J. Vac. Sci. Technol. B Microelectron. Nanom. Struct.* **1997**, *15*, 349.
14. Shi, L.; Majumdar, A. *J. Heat Transfer*, **2002**, *124*, 329–337.
15. Acharya, H.; Mozdzierz, N. J.; Keblinski, P.; Garde, S. *Ind. Eng. Chem. Res.* **2012**, *51*, 1767–1773.
16. Kim, Y.; Lenert, A.; Meyhofer, E.; Reddy, P. *Appl. Phys. Lett.* **2016**, *109*, 10–14.
17. Miao, R.; Xu, H.; Skripnik, M.; Cui, L.; Wang, K.; Pedersen, G. L.; Leijinse, M.; Pauly, F.; Wärnmark, K.; Meyhofer, E.; Reddy, P.; Linke, H. *Nano Lett.* **2018**, *18*, 5666–5672.
18. Widawsky, J. R.; Darancet, P.; Neaton, J. B.; Venkataraman, L. *Nano Lett.* **2012**, *12*, 354-358.
19. Yee, S. K.; Malen, J. A.; Majumdar, A.; Segalman, R. A. *Nano Lett.* **2011**, *11*, 4089-4094.
20. Schäfer, A.; Horn, H.; Ahlrichs, R. *J. Chem. Phys*. **1992**, *97*, 2571-2577
21. Zotti, L. A.; Bürkle, M.; Pauly, F.; Lee, W.; Kim, K; Jeong, W.; Asai, Y.; Reddy, P.; Cuevas J. C. *New J. Phys.* **2014**, *16*, 015004.
22. Quek, S. Y.; Venkataram, L.; Choi, H. J.; Louie, S. G.; Hybertsen, M. S.; Neaton, J. B. *Nano Lett.* **2007**, *7*, 3477–3482.
23. Karlström, O.; Linke, H.; Karlström, G.; Wacker. A. *Phys. Rev. B.* **2011**, *84*, 113415.
24. Weber, H. B.; Reichert, J.; Weigend, F.; Ochs, R.; Beckmann, D.; Mayor, M.; Ahlrichs, R.; v. Löhneysen, H. *Chem. Phys.* **2002**, *281*, 113–125.
25. Paulsson, M.; Brandbyge, M. *Phys. Rev. B* **2007**, *76*, 115117.

26. Bürkle, M.; Viljas, J. K.; Vonlanthen, D.; Mishchenko, A.; Schön, G.; Mayor, M.; Wandlowski, T.; Pauly, F. *Phys. Rev. B* **2012**, *85*, 075417.
